# Folliculin Contributes to VHL Tumor Suppressing Activity in Renal Cancer through Regulation of Autophagy

**DOI:** 10.1371/journal.pone.0070030

**Published:** 2013-07-29

**Authors:** Prabhat Bastola, Yiwen Stratton, Emily Kellner, Olga Mikhaylova, Ying Yi, Maureen A. Sartor, Mario Medvedovic, Jacek Biesiada, Jarek Meller, Maria F. Czyzyk-Krzeska

**Affiliations:** 1 Department of Cancer Biology, University of Cincinnati College of Medicine, Cincinnati, Ohio, United States of America; 2 VA Research Service, Department of Veterans Affairs, Cincinnati, Ohio, United States of America; 3 Department of Environmental Health, University of Cincinnati College of Medicine, Cincinnati, Ohio, United States of America; 4 Division of Biomedical Informatics, Cincinnati Children’s Hospital Medical Center, Cincinnati, Ohio, United States of America; 5 Department of Informatics, Nicolas Copernicus University, Torun, Poland; UCSF/VA Medical Center, United States of America

## Abstract

Von Hippel-Lindau tumor suppressor (VHL) is lost in the majority of clear cell renal cell carcinomas (ccRCC). Folliculin (FLCN) is a tumor suppressor whose function is lost in Birt-Hogg-Dubé syndrome (BHD), a disorder characterized by renal cancer of multiple histological types including clear cell carcinoma, cutaneous fibrofolliculoma, and pneumothorax. Here we explored whether there is connection between VHL and FLCN in clear cell renal carcinoma cell lines and tumors. We demonstrate that VHL regulates expression of FLCN at the mRNA and protein levels in RCC cell lines, and that FLCN protein expression is decreased in human ccRCC tumors with *VHL* loss, as compared with matched normal kidney tissue. Knockdown of FLCN results in increased formation of tumors by RCC cells with wild-type VHL in orthotopic xenografts in nude mice, an indication that FLCN plays a role in the tumor-suppressing activity of VHL. Interestingly, FLCN, similarly to VHL, is necessary for the activity of LC3C-mediated autophagic program that we have previously characterized as contributing to the tumor suppressing activity of VHL. The results show the existence of functional crosstalk between two major tumor suppressors in renal cancer, VHL and FLCN, converging on regulation of autophagy.

## Introduction

Renal cancer accounts for 2% to 3% of all adult malignancies in the US. In 2011 more than 64,770 new cases and 13,000 deaths were reported, and the incidence is steadily increasing by 2.5% per year. ccRCC represents the majority (85% to 90%) of adult kidney cancers and is the most malignant form. This cancer is characterized by an early loss of VHL tumor suppressor on the short arm of chromosome 3 in the majority of tumors (80%). It is well established that loss of VHL results in increased accumulation and activity of the hypoxia-inducible transcription factor (HIF), which in turns activates expression of genes that promote tumor growth. Among these are angiogenic factors, which lead to increased blood flow through the tumors, thus enhancing the delivery of nutrients and oxygen to cancer cells. Genes that stimulate anaerobic glycolysis are also activated, and this supports adaptation to reduced nutrient availability and shifts cancer cells towards metabolic pathways that promote tumor growth. Recently, we discovered that another tumor-suppressing pathway is regulated by VHL. We found that VHL is a major regulator of the process of macroautophagy (autophagy) [1]. We determined that VHL, by inducing microRNA-204, inhibited LC3B-mediated autophagy which is necessary for ccRCC tumor growth. Moreover, VHL, by inhibiting HIF, induced expression and activity of an LC3B paralog, LC3C. In contrast to LC3B, LC3C acts as a tumor suppressor [1].

In the process of searching for new VHL-induced genes that could participate in the tumor-suppressing activity of VHL, we discovered that reconstitution of wild-type VHL in RCC cells with lost *VHL* induced specific and significant enrichment for genes expressed from the Smith-Magenis locus (SM) on the short arm of chromosome 17p11.2. Smith-Magenis syndrome is a complex neurobehavioral disorder characterized by intellectual impairment, craniofacial and skeletal anomalies, and sleep disturbance [2]. Interestingly, this locus contains another kidney tumor-suppressor gene, folliculin (FLCN), whose activity is lost in the genetic autosomal-dominant disorder, Birt-Hogg-Dubé syndrome (BHD) [3]. BHD is characterized by skin fibrofolliculomas, pulmonary cysts, and spontaneous pneumothorax, as well as renal cancer of mixed histological types, including chromophobe, oncocytic, clear cell, and papillary type [3]. The purpose of this work was to determine if FLCN contributes to the tumor suppressing activity of VHL. We show that FLCN contributes to VHL-mediated suppression of tumor growth by affecting LC3B and LC3C autophagic pathways.

## Materials and Methods

### Cell Culture Methods and Treatments

Our method for generating pools of human VHL(−) and VHL(+) 786-O, A498, and Caki-1 cells were described by us before [1], [4], [5]. RCC4 cells were gift from Dr. Jamie Cairo (Jefferson University) and were described by us before [6]. Autophagic fluxes were performed as described before [1]. For protein or RNA extraction, cells were plated, 24 hr later medium was changed to that with either 10% or 0.1% serum, and cells were collected 48 hr later. Cells were then lysed in RIPA buffer with protease, phosphatase, and proteasomal inhibitors for PAGE analysis, or in TRI Reagent for RNA extraction. For RNAi experiments, pLKO.1-based shRNA constructs for FLCN (TRCN0000005969, TRCN0000005970, and TRCN0000010979; Open Biosystems) were VSV-G envelope packaged (Cincinnati Children’s Hospital Medical Center Viral Vector Core) and infected into cells, which were then treated with plasmid-appropriate selection reagents. VHL shRNA constructs were described before [5]. Transductions with lentiviral particles were performed using 2 µg/ml of polybrene.

### Human RCC Tumors

Fresh-frozen samples of ccRCC tumors (n = 128) and matched normal kidney samples (n = 114) were obtained and analyzed for *VHL* sequence and protein expression, as described before [4]. Specimens were analyzed for expression of protein and mRNA, also as described before.

### Quantitative RT-PCR

For mRNA analysis, the samples were first reverse transcribed using the TaqMan microRNA Reverse Transcription kit (Applied Biosystems), and quantified with real-time PCR on an Applied Biosystems 7900HT Fast Real-Time CRSystem. qRT-PCR was performed as described before, and using primers described in Table S4. Description of RNA microarrays is provided in Supplemental Methods.

### Western Blot Analysis

Total cellular lysates were obtained by lysing cells in RIPA buffer containing proteinase and phosphatase inhibitors. 5 to 30 µg of extracts were separated on 4% to 12% or 10% polyacrylamide gels and transferred onto PVDF membranes. Semi-quantitative immunoblotting for human RCC tumors and matched normal kidneys was performed as described previously [4]. The following polyclonal antibodies were obtained from Cell Signaling Technology (Danvar, MA): anti-FLCN, anti- S6 kinase 1 and T^389^ phosphorylation; anti- S6 and S^240/244^ phosphorylation; anti- 4EBP and T^37/46^ or S^65^ phosphorylation. Polyclonal anti-LC3B (Abcam, Cambridge, MA) and anti-LC3C (Abgent, San Diego, CA) were used. The following mouse monoclonal antibodies were used: anti-VHL (Pharmingen; San Jose, CA), anti-hemagglutinin (Boehringer Ingelheim, Austria); and anti-GAPDH (Abcam; Cambridge, MA).

### Mouse Xenograft Experiments

For the intra-kidney injections, 30,000 cells were resuspended in Matrigel to a final volume of 30 µl, and then slowly injected a few millimeters under the kidney capsula of 4- to 5-week-old athymic nude mice. After 10 to 12 weeks mice were sacrificed, tumors with kidneys were collected and fixed in 4% paraformaldehyde, paraffin embedded, and H&E stained or processed for S6/S6P staining in the Histopathology Core at in the Department of Pathology at CCHMC.

### Statistical Analysis of Quantified Data

Data normalization and analysis of RNA microarrays are described in Supplemental Methods. The data were deposited to Gene Expression Omnibus with accession number GSE47106. For descriptive statistics, data are expressed as mean ± SEM unless indicated otherwise. Analysis of differential expression was performed using t-test or one-way analysis of variance (ANOVA), followed by Tukey-Kramer multiple comparison tests. For quantification of the immunoblotting experiments the normalized values of protein abundance levels were log2 transformed to facilitate robust, outlier-insensitive analysis. Standard box-and-whisker plots were used to compare distributions of the normalized abundance measure for individual proteins. The differences between mean levels in two types of samples were assessed by using (two-sided) t-test and further validated by Wilcoxon test. Differences resulting in P<0.05 were considered to be statistically significant.

### Ethics Statement

All experiments on mice were performed in strict accordance to the protocol approved by University of Cincinnati Institutional Animal Care and Use Committee (Protocol Number: 06-07-21-01). All surgery was performed under isoflurane anesthesia and all efforts were made to minimize pain. All human samples were anonymous and exempted from University of Cincinnati Internal Review Board protocol requirements.

## Results

### Expression of FLCN is Induced by VHL

To identify the VHL-induced genes that could participate in the tumor-suppressing activity of VHL, we performed mRNA microarray experiments in which we compared gene expression in human RCC 786-O VHL(−) cells and the same cells with reconstituted, wild-type VHL (786-O VHL(+)). Statistical analysis identified 1,310 transcripts differentially expressed in the two cell types (FDR<0.1 and >50% change in expression level) (Table S1).

Interestingly, among the 588 transcripts induced by the reconstitution of VHL, we found a significant enrichment in genes located on the short arm of chromosome 17 (10.71% of all significantly induced genes), and particularly in cytoband 17p11.2, which contains the SM locus (1.7% of all significantly induced genes) (Table 1 and S2). There was also a concentration of genes induced by VHL on chromosome 14 (7.85% of all significantly induced genes) with the main cytoband being 14q22.1. VHL-mediated induction of several of the SM genes was further confirmed by quantitative RT-PCR (Fig. S1). In contrast, genes that were repressed by VHL were enriched on chromosome 7 (63 genes, 9.75% of all significantly repressed genes) and chromosome 9 (42 genes, 6.5% of all repressed genes) (Table S3).

**Table 1:** Distribution of genes on cytochromes 14 and 17 that exhibit statistically significant induction by VHL in 786-O cells.

CATEGORY	Term	Count	%	P Value	Rank	FDR
**CHROMOSOME**	14	74	7.85	2.20E-12	1	4.38E-10
**CHROMOSOME**	17	101	10.71	4.29E-09	2	4.27E-07
**CYTOBAND**	17p11.2	16	1.70	2.62E-06	3	1.74E-04
**CYTOBAND**	17p13.3	14	1.48	6.80E-06	4	3.38E-04
**CYTOBAND**	17p13.1	14	1.48	1.63E-05	5	6.47E-04
**CYTOBAND**	12p13	11	1.17	5.58E-04	6	1.85E-02
**CYTOBAND**	17p13.2	8	0.85	0.001459	7	4.15E-02
**CYTOBAND**	14q22.1	6	0.64	0.003118	8	7.76E-02
**CYTOBAND**	14q24.3	10	1.06	0.003914	9	8.65E-02

**Figure 1:**
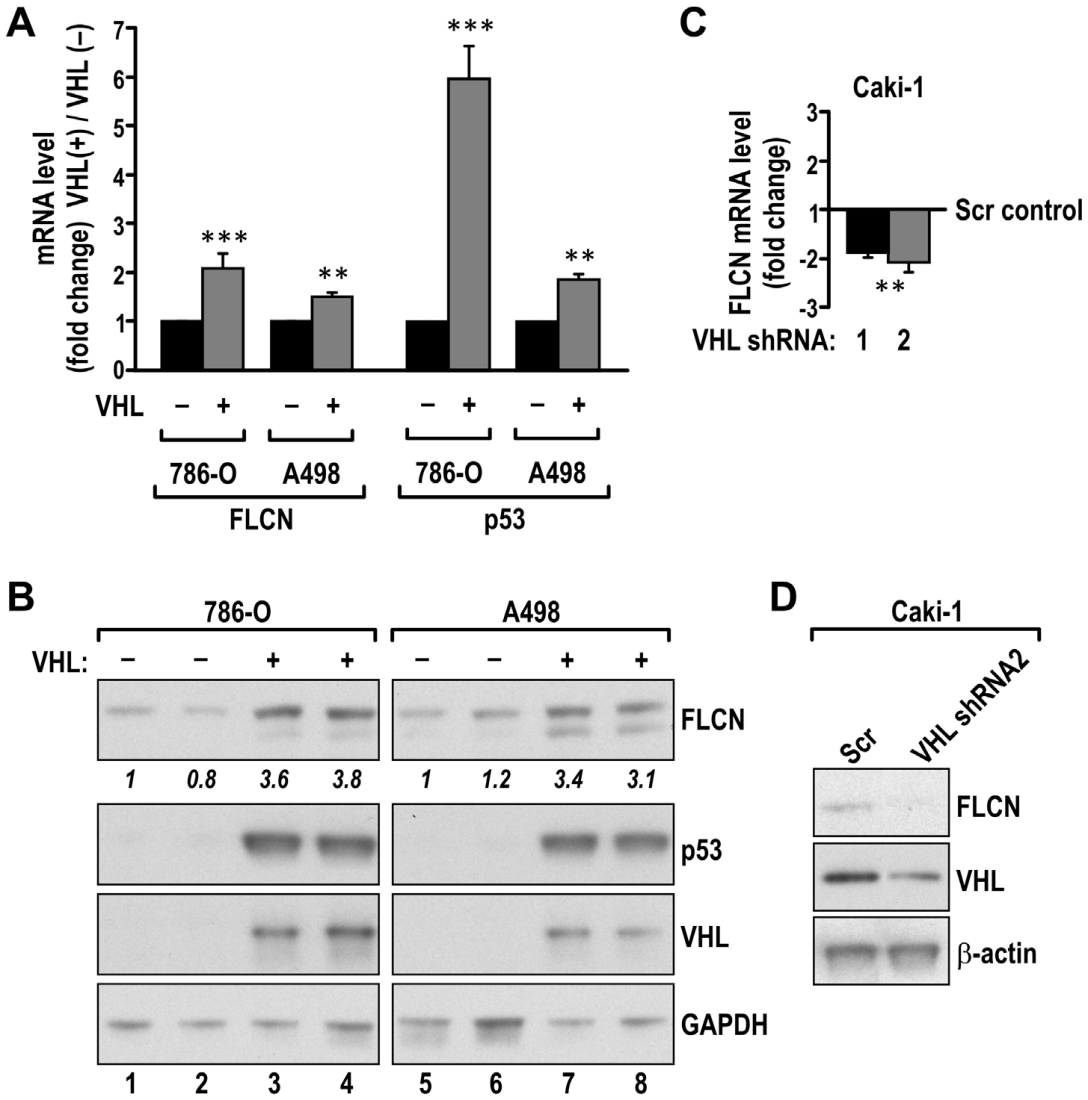
VHL induces expression of FLCN mRNA and protein. Reconstitution of VHL in 786-O and A498 cells led to induction of FLCN and p53 mRNAs (**A**) and proteins (**B**). Knockdown of VHL in Caki-1 cells resulted in decreased FLCN mRNA (**C**) and protein (**D**) levels; *** P<0.001, **P<0.01. Scr- scramble control virus.

The SM locus contains a gene encoding another renal kidney tumor suppressor, FLCN. Analysis of FLCN mRNA and protein revealed increased expression of both in 786-O and A498 cells with reconstituted VHL, as compared with parental VHL(-) cells (Fig. 1A and B). In addition, FLCN mRNA and protein expression were repressed in Caki1 renal cancer cells in which we knocked down endogenous VHL expression by using shRNA (Fig. 1C and 1D). The change in FLCN mRNA expression was in the range of 1.5 to 2 fold, similar to other SM genes (compare Fig. S1). However, induction at the level of protein was in the 3- to 4-fold range, an indication that posttranscriptional mechanisms play a role in this regulation. In these studies we also observed VHL-mediated induction of p53, another gene located on chromosome 17p13.1 in proximity of the SM locus, thus validating a finding that has previously been described by others [7].

**Figure 2:**
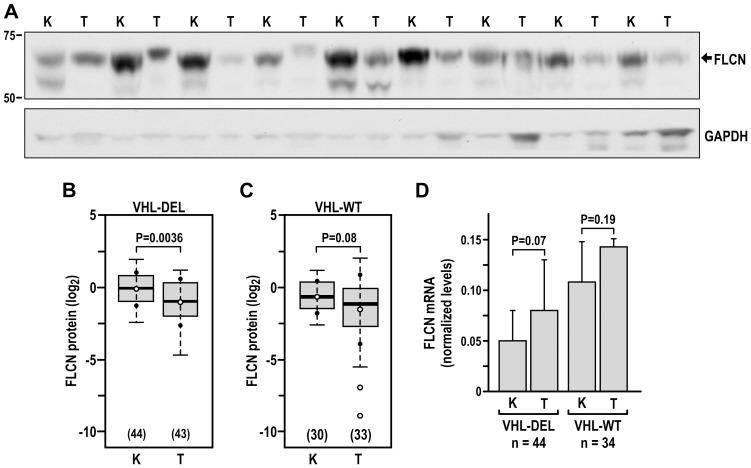
FLCN protein levels are reduced in human ccRCC with lost *VHL*. Analysis of FLCN protein (**A–C**) and mRNA (**D**) in samples of human ccRCC with known *VHL* status, as compared with adjacent kidney tissue. **A**. A representative western blot analysis of FLCN protein in pairs of kidney (K) and tumor with lost *VHL* (VHL-DEL) separated on 10% PAGE. The lower band may represent a degradation or posttranslational modification of FLCN. **B** and **C.** Quantification of FLCN protein expression in tumors that had lost *VHL* (VHL-DEL) and tumors with wild-type *VHL* (VHL-WT). The boxes represent lower and upper quartiles separated by the median (thick horizontal line) and the whiskers extend up to the minimum and maximum values, excluding points that are outside the 1.5 interquartile range from the box (marked as circles). Means ± SD of each distribution are indicated by closed dots and crosses on the whiskers, respectively. **D**. Quantitative RT-PCR measurement of FLCN mRNA levels in kidneys and ccRCC VHL-DEL and VHL-WT tumors.

Because VHL is lost in sporadic clear cell renal cell carcinoma (ccRCC), we measured the expression of FLCN in lysates from human ccRCC tumors as compared with adjacent kidney tissue in samples with known *VHL* status. We have previously described the population of human ccRCC-normal kidney pairs that we used for this analysis [4]. The FLCN protein was analyzed by semi-quantitative immunoblotting as previously described [4] and mRNA was determined by quantitative RT-PCR. Expression of FLCN protein was markedly decreased in ccRCC tumors as compared with normal kidneys in the population of ccRCC with the *VHL* deleted (Fig. 2A and 2B). In contrast, we did not see significant differences in FLCN levels when comparing ccRCC with wild-type *VHL* to the corresponding normal kidney tissue (Fig. 2C). Interestingly the levels of FLCN mRNA did not differ between tumor-kidney pairs in either group of tumors (Fig. 2D), supporting a role for posttranscriptional mechanisms in the regulation of FLCN expression.

### FLCN Contributes to VHL-mediated Tumor Suppression

**Figure 3:**
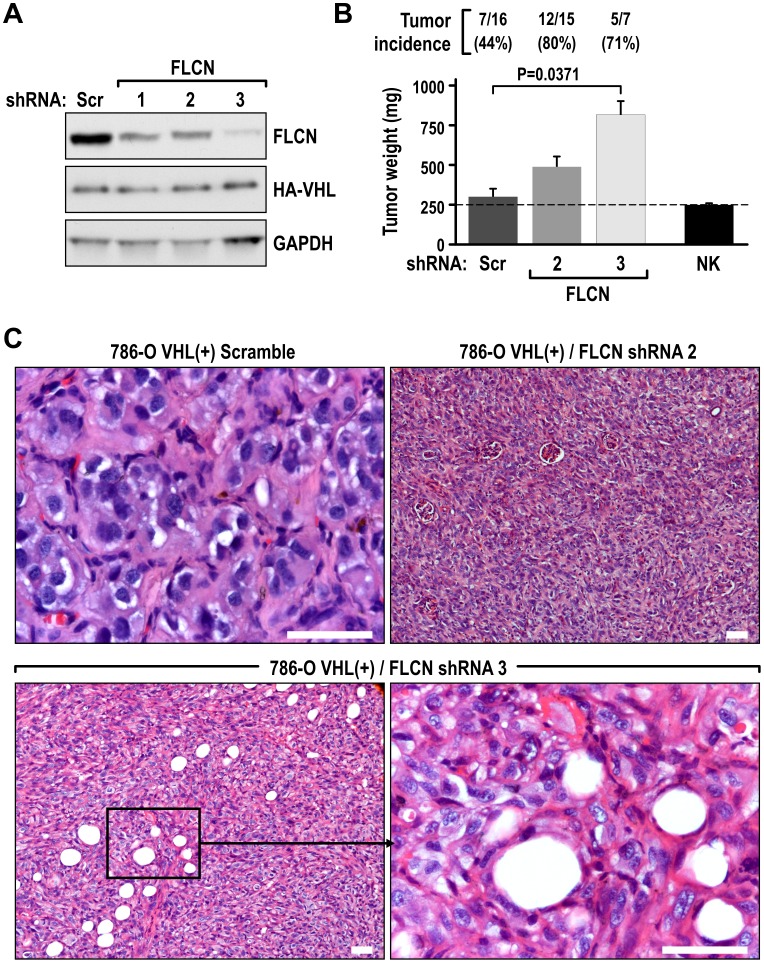
FLCN is necessary for the VHL-mediated tumor suppression. ShRNA-induced knockdown of FLCN promotes tumor formation by 786-O cells with reconstituted VHL. **A.** Western blot demonstrating a decrease in FLCN protein expression in cells stably expressing three different FLCN shRNAs as compared to cells transfected with scramble (Scr) control. **B.** Quantification of incidence and weight of tumors formed by the indicated cell lines in orthotopic xenografts. NK- average weight of normal kidneys. **C**. H&E staining of representative sections for tumors grown by VHL(+)/scramble control cells and VHL(+) cells with FLCN knockdowns. Scale bars = 50 µm.

To determine if there was a functional link between FLCN and the tumor-suppressing activity of VHL, we knocked down expression of FLCN in 786-O VHL(+) cells by using three different shRNAs (Fig. 3A). We then injected these cells under the kidney capsules of nude mice, which resulted in increased tumor incidence and larger tumor size, as compared with tumors derived from injected control cells that were transduced with lentiviruses containing scramble sequence (Fig. 3B). The tumors with FLCN knockdown had a highly malignant, dedifferentiated phenotype (Fig. 3C).

**Figure 4:**
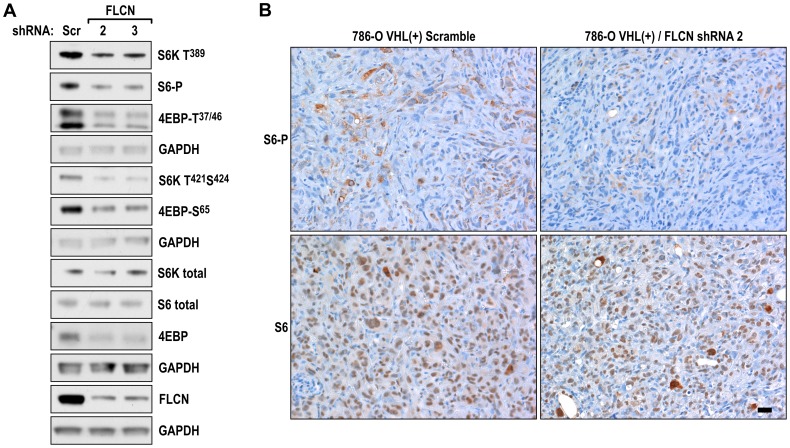
Knockdown of FLCN inhibits the mTOR pathway. **A**. Western blot analysis of mTOR downstream targets in 786-O VHL(+) cells stably expressing either scramble or two different FLCN shRNAs. The GAPDH loading control is shown for each group of immunoblots processed from the same gel. **B**. Representative sections of indicated tumors stained for S6 and S6P. Scale bar = 50 µm.

Because FLCN was shown to inhibit activity of the mTORC1 pathway in UOK257 cells and mouse models of BHD kidney cancer [8], [9], we analyzed the effects of FLCN knockdown on the activity of this pathway in the cells used for these orthotopic xenografts. Importantly, knockdown of FLCN resulted in decreased mTORC1 activity (Fig. 4A), as measured by phosphorylation of S6K, S6 and 4EBP, in cultured cells. We have also determined expression of S6 and S6-P is sections from xenografts tumors formed by VHL(+)/scramble transfected cells vs. tumors formed by these cells with FLCN knocked down. Consistently with the results obtained from cell culture experiments, sections from VHL(+)/FLCNKD tumors showed lower staining for S6-P as compared to the control tissues, while the staining for total S6 was similar (Fig. 4B). This result suggests that augmented activity of the mTORC1 pathway does not contribute to increased tumor growth in these cells.

**Figure 5:**
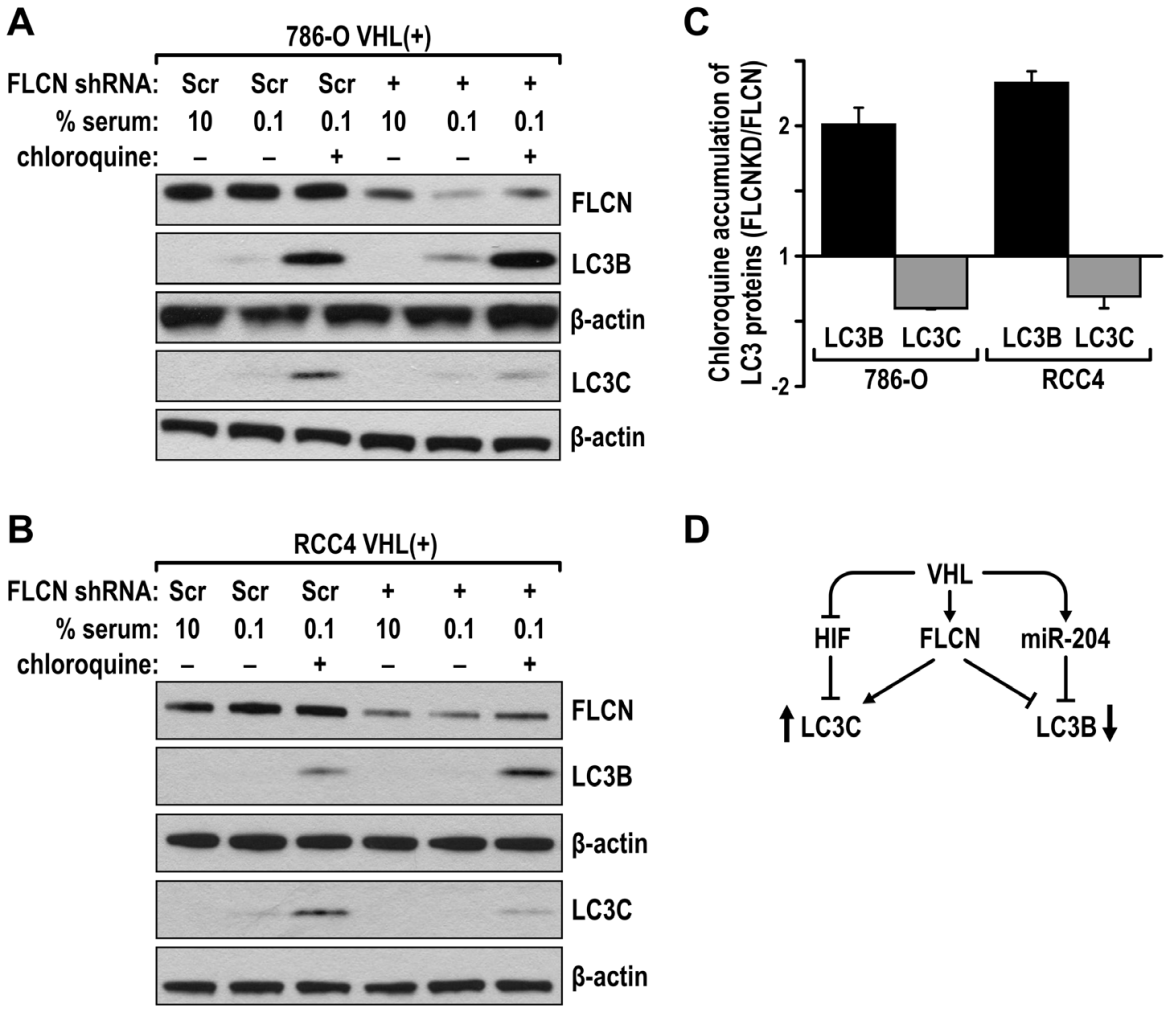
FLCN regulates autophagy. Knockdown of FLCN (shRNA 3) in 786-O (**A**) or RCC4 (**B**) VHL(+) cells reduces accumulation of tumor suppressive LC3C-II but induces expression of oncogenic LC3B-II. Western blots were probed with indicated antibodies. **C**. Quantification of 3 independent experiments as shown in panel A and B. LC3 protein levels measured by optical density were first normalized to GAPDH. Then the ratio of LC3 was calculated between LC3 levels measured in chloroquine treated samples in cells with FLCN knocked down and control cells (compare lane 6 to lane 3 in panels A and B). **D**. Model of the proposed regulation.

Recently we have described that VHL suppresses LC3B-mediated, pro-oncogenic autophagy while it stimulates a novel tumor suppressing autophagic program, mediated by LC3C [1]. LC3s are well established autophagic regulators necessary for the formation of the double membrane autophagosomes and for acquisition of the cargo. Thus we investigated if FLCN may exercise its tumor suppressing activity downstream from VHL by regulating LC3C or LC3B autophagy. LC3C is induced by starvation in VHL(+) cells, thus in these experiments, VHL(+) 786-O and RCC4 cells were starved in 0.1% serum for 48 hr and then treated for 1 hr with chloroquine, a lysosomal inhibitor in order to inhibit autophagic flow and the accumulation of the faster migrating, lipidated form (LC-II) of LC3B and LC3C was measured by western blotting. We found that knockdown of FLCN resulted in a substantial decrease in the accumulation of LC3C with an increase in LC3B both cell lines (Fig. 5). The change in LC3B in VHL(+) RCC cells was not associated with any consistent changes in miR-204 (data not shown). This result indicates that FLCN regulates autophagy similarly to VHL, and thus the effects on autophagy may be partially responsible for the contribution of FLCN to the tumor suppressing activity of VHL.

## Discussion

Here we reported that VHL regulates expression of FLCN and that, in turn, FLCN participates in the tumor suppressing activity of VHL. The observed effects of FLCN knockdown on tumor growth are consistent with the negative effect of FLCN overexpression on the formation of tumors by 786-O VHL(−) cells, as observed by others [10]. Importantly, FLCN regulates LC3B and LC3C autophagic programs in a manner similar to VHL, i.e. FLCN inhibits LC3B and stimulates LC3C autophagic activity (Fig. 5D). Because we demonstrated that both LC3B and LC3C exercise important and opposite effects on growth of ccRCC tumors [1], we propose that the contribution of FLCN to the tumor-suppressing activity of VHL results, at least partially, from its effects on autophagy (Fig. 5D). Regulation of LC3B by FLCN likely represents a mechanism additional to the previously described by us VHL-mediated inhibition of LC3B through miR-204, as we did not measure consistent effects of FLCN on miR-204. At the same time both tumor suppressors could induce expression of LC3C through repression of HIF, since LC3C is a HIF-inhibited gene. VHL is well recognized for its ability to inhibit HIF-αs protein expression and activity. Recently, FLCN has also been reported to diminish HIF activity, even though it does not affect the accumulation of HIF-αs subunits [11].

The specific role of FLCN in regulation of autophagy is further supported by the recently identified presence of DENN domain in the carboxy-terminal region of FLCN [12]. This motif is present also in folicullin associated protein-1 and 2 (FNIP1 and FNIP2 [13]), and, interestingly in another protein expressed from the SM locus, SMCR8 [13], which also we found to be induced by VHL (Fig. S1). These proteins are acting as GDP-GTP exchange factors (GEFs) for RAB GTPases, potentially necessary for autophagy associated trafficking and fusion [11], [13].

The SM locus has not been linked to ccRCC. Previously, only one gene from this locus, TNFSF13 (tumor necrosis factor superfamily member 13), had been studied in this regard. It was found to exhibit decreased mRNA levels in ccRCC as compared with normal kidney. In addition, higher levels of this receptor were shown to be correlated with better overall and disease-free survival [14]. However it is possible that further analysis will reveal additional autophagy-related functions of genes expressed from SM locus.

FLCN contributes to VHL-induced tumor suppression by a mechanism that does not appear to involve activation of the mTOR pathway. Indeed, in 786-O cells with reconstituted VHL and FLCN knocked-down, we actually showed a strong reduction in several phosphorylation events that are used as indicators of mTOR activity. Consistently with the inhibition of mTOR we measured increased accumulation of LC3B, a widely used autophagic readout, and this increased LC3B-mediated autophagy may contribute to tumor growth. The observed effects of FLCN on mTOR activity are consistent with several publications reporting a positive correlation between levels of FLCN and mTOR in U251, HEK293, HK-2, ACHN, 786-O VHL(−) cells [15]. However, the current canonical understanding of the FLCN-mTOR connection is that FLCN negatively regulates the mTOR pathway [8], [9], [16]. In that respect, the human UOK257 cell line derived from a BHD patient, that does not express VHL, shows a high level of mTOR activity, and mice with FLCN knockout demonstrate high levels of S6 phosphorylation [8], [9]. The reason for this discrepancy is currently not understood, but perhaps the data point towards a compartmentalized effect of FLCN on mTOR that is affected by the cellular context, such as activity of VHL. Nevertheless, the connection of VHL to a tumor suppressor that regulates a metabolic pathway in renal cancer may reveal a novel aspect of VHL tumor-suppressing activity that is complementary to its roles in the regulation of angiogenesis and autophagy.

Importantly, FLCN protein levels are diminished in human ccRCC with *VHL* loss, further supporting a role for this tumor suppressor in the growth of ccRCC. So far, no FLCN mutations or changes in mRNA levels have been measured in ccRCC. This convergence in the functions of VHL and FLCN is of special interest considering the fact that clear cell carcinoma can be part of the multihistological type of renal cancer that occurs in BHD syndrome.

The nature of the direct molecular mechanism by which VHL induces FLCN renal tumor suppressors remains to be determined, but is likely to involve multiple mechanisms. Because we measured an effect of VHL on FLCN mRNA steady-state levels in RCC cell lines, we expect some level of transcriptional induction or stabilization of FLCN mRNA. In that respect we did not find any effects of p53 knockdown on FLCN expression. Because we did not find changes in the FLCN mRNA levels between human kidney-tumor pairs although we found difference in the steady-state of FLCN protein, we propose that there is also an important translational component to this regulation, possibly depending on the specificity of different kidney cell populations. One explanation is that VHL may repress expression of specific microRNAs, which in turn repress FLCN translation.

In summary, with the current study, we have provided first evidence for a direct connection between the VHL and FLCN tumor-suppressing pathways converging on regulation of autophagy in renal cancer.

## Supporting Information

Figure S1 **VHL induces expression of several genes expressed from the Smith-Magenis locus in 786-0 and A498 cells.** The graph shows changes in the expression of individual mRNAs based on quantitative qRT-PCR measurements. Fold change represents the difference in expression of mRNAs in VHL(+) versus VHL(−) cells. The sequence of primers is provided in.
(PDF)

Table S1 **List of genes significantly repressed or induced by VHL in 786-O cells.** Fold change represents VHL (+)/VHL(−) ratio.
(DOCX)

Table S2 **Genes significantly induced by VHL and enriched for specific chromosome or cytoband.**
(XLS)

Table S3 **Genes significantly reduced by VHL and enriched for specific chromosome or cytoband.**
(DOCX)

Table S4 **Sequence of primers for the indicated genes used in qRT-PCR.**
(DOCX)
