# Supplementary material for: Folliculin Contributes to VHL Tumor Suppressing Activity in Renal Cancer through Regulation of Autophagy
Source: PLoS One. 2013 Jul 29;8(7):e70030. doi: 10.1371/journal.pone.0070030 (PMC3726479; doi:10.1371/journal.pone.0070030)
Supplement: Figure S1 — VHL induces expression of several genes expressed from the Smith-Magenis locus in 786-0 and A498 cells. The graph shows changes in the expression of individual mRNAs based on quantitative qRT-PCR measurements. Fold change represents the difference in expression of mRNAs in VHL(+) versus VHL(−) cells. The sequence of primers is provided in. (PDF) [file pone.0070030.s001.pdf]

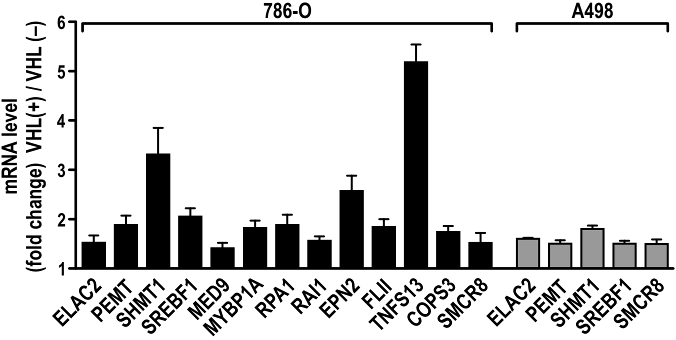

Figure S1. VHL in 786-0 and A498 cells induces expression of several genes expressed from the Smith-Magenis locus. The graph shows changes in the expression of individual mRNAs based on quantitative qRT-PCR measurements. Fold change represents the difference in expression of mRNAs in VHL(+) versus VHL(-) cells. The sequence of primers is provided in Table S3.
