# Supplementary material for: Folliculin Contributes to VHL Tumor Suppressing Activity in Renal Cancer through Regulation of Autophagy
Source: PLoS One. 2013 Jul 29;8(7):e70030. doi: 10.1371/journal.pone.0070030 (PMC3726479; doi:10.1371/journal.pone.0070030)
Supplement: Table S1 — List of genes significantly repressed or induced by VHL in 786-O cells. Fold change represents VHL (+)/VHL(−) ratio. (DOCX) [file pone.0070030.s002.docx]

**Table S1: List of All Genes Significantly Repressed or Induced by VHL. Fold Change represents VHL (+)/VHL(-) Ratio**

| **clone** | **Name** | **UnigeneID** | **cytoband** | **symbol** | **avg intensity** | **DF** | **Fold Change** | **p-value** | **FDR** |
| --- | --- | --- | --- | --- | --- | --- | --- | --- | --- |
| NM_001305 | claudin 4 | Hs.5372 | 7q11.23 | CLDN4 | 699 | 16 | -12.37 | 1.15E-07 | 9.90E-05 |
| BC001521 | UDP glycosyltransferase 1 family, polypeptide A10 | Hs.516772 | 2q37 | UGT1A10 | 122 | 10 | -10.72 | 2.59E-05 | 3.98E-03 |
| AF200348 | Melanoma associated gene | Hs.118893 | 2pter-p25.1 | D2S448 | 197 | 14 | -7.59 | 1.53E-06 | 6.24E-04 |
| NM_003081 | synaptosomal-associated protein, 25kDa | Hs.221974 | 20p12-p11.2 | SNAP25 | 92 | 8 | -7.46 | 1.07E-06 | 5.21E-04 |
| NM_001072 | UDP glycosyltransferase 1 family, polypeptide A10 | Hs.278896 | 2q37 | UGT1A10 | 117 | 16 | -6.91 | 6.94E-08 | 8.29E-05 |
| NM_002285 | lymphoid nuclear protein related to AF4 | Hs.38070 | 2q11.2-q12 | LAF4 | 163 | 5 | -6.60 | 1.03E-04 | 8.97E-03 |
| AK022198 | UDP-N-acetyl-alpha-D-galactosamine:polypeptide N-acetylgalactosaminyltransferase 5 (GalNAc-T5) | Hs.443716 | 2q24.2 | GALNT5 | 106 | 5 | -6.52 | 3.50E-04 | 1.83E-02 |
| NM_002889 | retinoic acid receptor responder (tazarotene induced) 2 | Hs.37682 | 7q36.1 | RARRES2 | 262 | 7 | -6.26 | 1.85E-04 | 1.25E-02 |
| NM_002185 | interleukin 7 receptor | Hs.362807 | 5p13 | IL7R | 113 | 8 | -5.90 | 1.26E-05 | 2.50E-03 |
| NM_005557 | keratin 16 (focal non-epidermolytic palmoplantar keratoderma) | Hs.432448 | 17q12-q21 | KRT16 | 91 | 7 | -5.60 | 8.03E-03 | 9.61E-02 |
| AK022092 | Homo sapiens cDNA FLJ12030 fis, clone HEMBB1001868. | Hs.160572 | 4q24 | NFKB1 | 102 | 6 | -5.54 | 3.19E-03 | 6.18E-02 |
| NM_025067 | hypothetical protein FLJ14106 | Hs.287872 | 2p24.1 | FLJ14106 | 275 | 7 | -5.45 | 2.91E-04 | 1.63E-02 |
| BC011535 | DKFZP566K1924 protein | Hs.371496 | 2p13.3 | DKFZP566K1924 | 496 | 13 | -5.33 | 6.16E-08 | 8.70E-05 |
| AK022619 | Homo sapiens cDNA FLJ12557 fis, clone NT2RM4000783. | Hs.296724 | 7q11.23 | AUTS2 | 111 | 5 | -5.28 | 5.73E-04 | 2.42E-02 |
| AK054643 | hypothetical protein MGC45873 | Hs.405987 | 1p36.33 | MGC45873 | 388 | 16 | -4.95 | 1.98E-08 | 7.70E-05 |
| U67784 | G protein-coupled receptor | Hs.231853 | 2q37.3 | RDC1 | 118 | 12 | -4.90 | 1.71E-06 | 6.65E-04 |
| NM_002994 | chemokine (C-X-C motif) ligand 5 | Hs.89714 | 4q12-q13 | CXCL5 | 139 | 7 | -4.90 | 3.48E-04 | 1.83E-02 |
| AB023204 | erythrocyte membrane protein band 4.1-like 3 | Hs.103839 | 18p11.32 | EPB41L3 | 152 | 11 | -4.89 | 1.57E-06 | 6.27E-04 |
| NM_057091 | artemin | Hs.194689 | 1p33-p32 | ARTN | 135 | 11 | -4.81 | 1.05E-06 | 5.25E-04 |
| NM_000389 | cyclin-dependent kinase inhibitor 1A (p21, Cip1) | Hs.370771 | 6p21.2 | CDKN1A | 511 | 16 | -4.78 | 4.89E-08 | 1.09E-04 |
| NM_000238 | potassium voltage-gated channel, subfamily H (eag-related), member 2 | Hs.188021 | 7q35-q36 | KCNH2 | 128 | 10 | -4.70 | 5.82E-05 | 6.75E-03 |
| NM_000596 | insulin-like growth factor binding protein 1 | Hs.401316 | 7p13-p12 | IGFBP1 | 201 | 15 | -4.69 | 8.33E-07 | 5.17E-04 |
| M30474 | gamma-glutamyltransferase 2 | Hs.289098 | 22q11.23 | GGT2 | 230 | 10 | -4.62 | 1.27E-04 | 1.03E-02 |
| NM_013421 | gamma-glutamyltransferase 1 | Hs.352119 | 22q11.23 | GGT1 | 272 | 16 | -4.56 | 3.60E-04 | 1.84E-02 |
| NM_007150 | zinc finger protein 185 (LIM domain) | Hs.16622 | Xq28 | ZNF185 | 98 | 7 | -4.56 | 4.27E-04 | 2.05E-02 |
| NM_012395 | PFTAIRE protein kinase 1 | Hs.57856 | 7q21-q22 | PFTK1 | 157 | 14 | -4.30 | 3.26E-05 | 4.60E-03 |
| AF064839 | Homo sapiens map 3p21; 3.15 cR from WI-9324 repeat region, complete sequence | Hs.518147 | 3p14.1 | na | 257 | 14 | -4.29 | 1.10E-05 | 2.31E-03 |
| NM_032928 | hypothetical protein MGC14141 | Hs.356744 | 9q34.3 | MGC14141 | 1555 | 14 | -4.21 | 2.35E-09 | 1.22E-05 |
| NM_006517 | solute carrier family 16 (monocarboxylic acid transporters), member 2 (putative transporter) | Hs.75317 | Xq13.2 | SLC16A2 | 79 | 8 | -4.21 | 4.08E-03 | 6.84E-02 |
| AB002438 | sema domain, transmembrane domain (TM), and cytoplasmic domain, (semaphorin) 6A | Hs.443012 | 5q23.1 | SEMA6A | 142 | 10 | -4.16 | 1.64E-05 | 2.87E-03 |
| NM_002133 | heme oxygenase (decycling) 1 | Hs.202833 | 22q12 | HMOX1 | 472 | 16 | -4.12 | 6.09E-05 | 6.85E-03 |
| NM_002276 | keratin 19 | Hs.309517 | 17q21.2 | KRT19 | 2187 | 16 | -4.11 | 1.12E-07 | 1.03E-04 |
| NM_003810 | tumor necrosis factor (ligand) superfamily, member 10 | Hs.387871 | 3q26 | TNFSF10 | 602 | 15 | -4.09 | 3.80E-05 | 5.13E-03 |
| NM_005023 | protein geranylgeranyltransferase type I, beta subunit | Hs.254006 | 5q23.1 | PGGT1B | 126 | 7 | -4.05 | 1.00E-04 | 8.89E-03 |
| AK022236 | Homo sapiens cDNA FLJ12174 fis, clone MAMMA1000707 | Hs.221941 | 14q24.2 | na | 70 | 7 | -4.04 | 4.67E-03 | 7.33E-02 |
| AK056184 | zinc finger protein 337 | Hs.139240 | 20p11.21 | ZNF337 | 122 | 5 | -4.02 | 3.06E-03 | 6.02E-02 |
| NM_021023 | complement factor H related 3 | Hs.2637 | 1q32 | FHR-3 | 168 | 7 | -3.98 | 9.25E-04 | 3.24E-02 |
| AL512761 | hypothetical protein LOC284361 | Hs.250465 | 19q13.33-q13.41 | LOC284361 | 534 | 16 | -3.98 | 1.56E-03 | 4.28E-02 |
| AK001110 | Homo sapiens cDNA FLJ10248 fis, clone HEMBB1000706. | Hs.479989 | 1p35.2 | FLJ20045 | 197 | 5 | -3.95 | 3.59E-04 | 1.84E-02 |
| BC007116 | similar to DNA-binding protein; zinc finger protein 253 | Hs.334568 | 19p13.11 | LOC199777 | 130 | 5 | -3.93 | 8.44E-05 | 7.94E-03 |
| NM_003571 | beaded filament structural protein 2, phakinin | Hs.435710 | 3q21-q25 | BFSP2 | 168 | 6 | -3.87 | 4.69E-03 | 7.33E-02 |
| AK022274 | protein tyrosine phosphatase, receptor type, A | Hs.306676 | 20p13 | PTPRA | 126 | 5 | -3.76 | 1.39E-03 | 4.08E-02 |
| NM_004438 | EphA4 | Hs.73964 | 2q36.1 | EPHA4 | 185 | 14 | -3.76 | 1.04E-05 | 2.20E-03 |
| NM_024554 | piggyBac transposable element derived 5 | Hs.12247 | 1q42.2 | PGBD5 | 221 | 15 | -3.69 | 1.94E-06 | 7.35E-04 |
| NM_001914 | cytochrome b-5 | Hs.83834 | 18q23 | CYB5 | 207 | 16 | -3.61 | 2.14E-05 | 3.46E-03 |
| NM_014399 | transmembrane 4 superfamily member 13 | Hs.364544 | 7p21.2 | TM4SF13 | 678 | 13 | -3.52 | 7.72E-04 | 2.95E-02 |
| NM_001630 | annexin A8 | Hs.87268 | 10q11.2 | ANXA8 | 117 | 14 | -3.50 | 2.50E-05 | 3.88E-03 |
| NM_002698 | POU domain, class 2, transcription factor 2 | Hs.1101 | 19q13.31 | POU2F2 | 108 | 7 | -3.49 | 4.40E-03 | 7.11E-02 |
| NM_020633 | vomeronasal 1 receptor 1 | Hs.388810 | 19q13.4 | VN1R1 | 160 | 5 | -3.48 | 1.83E-05 | 3.09E-03 |
| AL137602 | zinc finger protein 44 (KOX 7) | Hs.501604 | 19p13.2 | ZNF44 | 87 | 13 | -3.42 | 9.68E-04 | 3.32E-02 |
| NM_004681 | eukaryotic translation initiation factor 1A, Y-linked | Hs.461178 | Yq11.222 | EIF1AY | 347 | 14 | -3.40 | 1.98E-05 | 3.23E-03 |
| U80764 | Human EST clone 122887 mariner transposon Hsmar1 sequence | Hs.522272 | 9q22.1 | FLJ35866 | 82 | 6 | -3.40 | 4.23E-03 | 7.01E-02 |
| AL049280 | chromosome Y open reading frame 15B | Hs.145010 | Yq11.222 | CYorf15B | 133 | 5 | -3.40 | 1.06E-03 | 3.50E-02 |
| M96843 | striated muscle contraction regulatory protein | Hs.476554 | 3p21.1 | ID2B | 699 | 16 | -3.39 | 2.08E-06 | 7.71E-04 |
| AK056229 | Homo sapiens cDNA FLJ31667 fis, clone NT2RI2004840. | Hs.419123 | 1pter-q31.3 | KIAA0478 | 81 | 8 | -3.37 | 6.74E-03 | 8.81E-02 |
| NM_000747 | cholinergic receptor, nicotinic, beta polypeptide 1 (muscle) | Hs.330386 | 17p13.1 | CHRNB1 | 172 | 11 | -3.36 | 2.09E-03 | 4.97E-02 |
| NM_001505 | G protein-coupled receptor 30 | Hs.113207 | 7p22 | GPR30 | 83 | 12 | -3.35 | 1.97E-03 | 4.81E-02 |
| AF055019 | homeodomain interacting protein kinase 2 | Hs.397465 | 7q32-q34 | HIPK2 | 547 | 16 | -3.35 | 4.86E-05 | 6.08E-03 |
| NM_032798 | Hypothetical protein FLJ14503 |  |  |  | 132 | 14 | -3.34 | 1.09E-04 | 9.18E-03 |
| NM_002005 | feline sarcoma oncogene | Hs.7636 | 15q26.1 | FES | 137 | 7 | -3.32 | 7.76E-04 | 2.96E-02 |
| NM_033334 | nuclear receptor subfamily 6, group A, member 1 | Hs.195161 | 9q33-q34.1 | NR6A1 | 101 | 6 | -3.32 | 8.14E-03 | 9.65E-02 |
| AL137296 | M8 protein | Hs.126516 | 20p13 | LOC149830 | 84 | 6 | -3.31 | 2.14E-03 | 5.02E-02 |
| NM_002864 | pregnancy-zone protein | Hs.480143 | 12p13-p12.2 | PZP | 97 | 8 | -3.31 | 2.67E-03 | 5.63E-02 |
| NM_032756 | hypothetical protein MGC15668 | Hs.162717 | 1p34.1 | MGC15668 | 152 | 5 | -3.29 | 4.94E-03 | 7.46E-02 |
| AK055922 | Homo sapiens cDNA FLJ20653 fis, clone KAT01739 | Hs.388761 | 9p13.1 | FLJ22611 | 2030 | 15 | -3.28 | 1.09E-06 | 5.13E-04 |
| NM_022454 | SRY (sex determining region Y)-box 17 | Hs.98367 | 8q11.23 | SOX17 | 853 | 16 | -3.27 | 2.84E-03 | 5.82E-02 |
| AK027161 | hypothetical protein FLJ20535 | Hs.288772 | 11q23.2 | FLJ20535 | 137 | 14 | -3.27 | 1.41E-03 | 4.11E-02 |
| AF323540 | apolipoprotein L, 1 | Hs.114309 | 22q13.1 | APOL1 | 1489 | 16 | -3.24 | 1.00E-06 | 5.38E-04 |
| AK023413 | hypothetical protein FLJ20559 | Hs.232459 | 9q21.31 | FLJ20559 | 155 | 14 | -3.22 | 1.81E-04 | 1.24E-02 |
| AK056500 | Homo sapiens cDNA FLJ31938 fis, clone NT2RP7006539 |  |  |  | 87 | 7 | -3.22 | 7.08E-03 | 9.03E-02 |
| AL049227 | Homo sapiens mRNA; cDNA DKFZp564N1116 (from clone DKFZp564N1116) | Hs.124776 | 4q24 | DHRS6 | 186 | 8 | -3.21 | 2.41E-03 | 5.33E-02 |
| AK021793 | hypothetical protein LOC286025 | Hs.292453 | 7q34 | LOC286025 | 190 | 5 | -3.21 | 1.64E-03 | 4.41E-02 |
| AK057785 | hypothetical protein FLJ25056 | Hs.253773 | 12q14.3 | FLJ25056 | 82 | 5 | -3.19 | 4.42E-03 | 7.14E-02 |
| NM_001008 | ribosomal protein S4, Y-linked | Hs.180911 | Yp11.3 | RPS4Y | 223 | 12 | -3.19 | 8.50E-03 | 9.80E-02 |
| NM_017718 | Hypothetical protein FLJ20220 |  |  |  | 172 | 12 | -3.18 | 4.49E-05 | 5.72E-03 |
| AK055887 | Homo sapiens LOC352165 (LOC352165), mRNA | Hs.192711 | 22q11.21 | FLJ32575 | 114 | 7 | -3.16 | 2.45E-04 | 1.45E-02 |
| NM_025051 | hypothetical protein FLJ23022 | Hs.287717 | 17p11.2 | FLJ23022 | 96 | 5 | -3.15 | 3.23E-03 | 6.21E-02 |
| AF070674 | baculoviral IAP repeat-containing 3 | Hs.127799 | 11q22 | BIRC3 | 687 | 12 | -3.13 | 3.26E-04 | 1.76E-02 |
| NM_018534 | neuropilin 2 | Hs.368746 | 2q33.3 | NRP2 | 380 | 13 | -3.13 | 1.47E-04 | 1.11E-02 |
| NM_001701 | bile acid Coenzyme A: amino acid N-acyltransferase (glycine N-choloyltransferase) | Hs.284712 | 9q22.3 | BAAT | 206 | 7 | -3.12 | 1.57E-03 | 4.29E-02 |
| NM_001585 | chromosome 22 open reading frame 1 | Hs.159538 | 22q13.31 | C22orf1 | 177 | 15 | -3.12 | 4.45E-04 | 2.08E-02 |
| NM_017416 | interleukin 1 receptor accessory protein-like 2 | Hs.272354 | Xq22.2-q22.3 | IL1RAPL2 | 68 | 10 | -3.11 | 6.12E-03 | 8.36E-02 |
| AK055111 | Homo sapiens cDNA FLJ42700 fis, clone BRAMY3004900 | Hs.398039 | 22q12 | APOL2 | 148 | 10 | -3.07 | 7.25E-03 | 9.15E-02 |
| AK057226 | Homo sapiens cDNA FLJ32664 fis, clone TESTI1000088. | Hs.123393 | 11q22.3 | MMP20 | 114 | 8 | -3.06 | 1.99E-03 | 4.84E-02 |
| AB033070 | KIAA1244 protein | Hs.194408 | 6q23.3 | KIAA1244 | 78 | 8 | -3.06 | 1.07E-04 | 9.16E-03 |
| AK055357 | squalene epoxidase | Hs.71465 | 8q24.1 | SQLE | 147 | 15 | -3.03 | 2.41E-03 | 5.33E-02 |
| AK023690 | Homo sapiens cDNA FLJ13628 fis, clone PLACE1011054. | Hs.380519 | 1p36 | MMEL2 | 105 | 7 | -3.03 | 1.83E-03 | 4.66E-02 |
| BC016588 | hypothetical protein MGC27044 | Hs.351233 | 1q31.3 | MGC27044 | 205 | 13 | -3.02 | 7.06E-03 | 9.01E-02 |
| AK000119 | Homo sapiens cDNA FLJ20112 fis, clone COL05405 | Hs.482250 | 5q12.1 | na | 79 | 5 | -3.00 | 7.52E-04 | 2.90E-02 |
| AK021569 | Homo sapiens cDNA FLJ11507 fis, clone HEMBA1002160. | Hs.434491 | 4p15.33 | FLJ90013 | 163 | 6 | -2.99 | 3.79E-03 | 6.63E-02 |
| BC005233 | pancreatic lipase-related protein 1 | Hs.73923 | 10q26.12 | PNLIPRP1 | 217 | 16 | -2.98 | 1.51E-03 | 4.24E-02 |
| NM_025042 | GTF2I repeat domain containing 1 | Hs.430854 | 7q11.23 | GTF2IRD1 | 212 | 5 | -2.98 | 5.49E-03 | 7.87E-02 |
| AL080135 | Homo sapiens LOC347571 (LOC347571), mRNA | Hs.433656 | 6pter-p21.31 | MDC1 | 221 | 15 | -2.95 | 6.01E-05 | 6.81E-03 |
| NM_052969 | ribosomal protein L39-like | Hs.132748 | 3q27 | RPL39L | 111 | 7 | -2.94 | 2.43E-03 | 5.34E-02 |
| BC014245 | collagen triple helix repeat containing 1 | Hs.283713 | 8q22.3 | CTHRC1 | 108 | 8 | -2.92 | 2.29E-04 | 1.39E-02 |
| NM_000959 | prostaglandin F receptor (FP) | Hs.89418 | 1p31.1 | PTGFR | 130 | 11 | -2.92 | 9.14E-05 | 8.40E-03 |
| AK022416 | hypothetical protein FLJ12287 similar to semaphorins | Hs.408846 | 1q22 | FLJ12287 | 106 | 11 | -2.91 | 3.85E-03 | 6.69E-02 |
| NM_014226 | renal tumor antigen | Hs.104119 | 14q32 | RAGE | 499 | 14 | -2.91 | 3.60E-03 | 6.47E-02 |
| M29873 | cytochrome P450, family 2, subfamily B, polypeptide 7 pseudogene | Hs.415794 | 19q13.2 | CYP2B7 | 123 | 16 | -2.88 | 3.26E-03 | 6.23E-02 |
| AL137660 | hypothetical protein FLJ10300 | Hs.445204 | 7q36.3 | FLJ10300 | 113 | 5 | -2.88 | 3.72E-03 | 6.58E-02 |
| NM_004843 | class I cytokine receptor | Hs.132781 | 19p13.11 | WSX1 | 156 | 14 | -2.88 | 2.04E-03 | 4.90E-02 |
| NM_003918 | glycogenin 2 | Hs.380757 | Xp22.3 | GYG2 | 112 | 6 | -2.87 | 2.26E-03 | 5.19E-02 |
| AK023481 | osteoblast specific factor 2 (fasciclin I-like) | Hs.136348 | 13q13.3 | OSF-2 | 77 | 6 | -2.86 | 7.95E-03 | 9.57E-02 |
| NM_032827 | basic helix-loop-helix transcription factor 6 | Hs.135569 | 2p11.2 | HATH6 | 220 | 14 | -2.82 | 6.21E-05 | 6.89E-03 |
| AK000455 | hypothetical gene MGC16733 similar to CG12113 | Hs.141245 | 11q13.4 | MGC16733 | 349 | 16 | -2.82 | 4.41E-05 | 5.65E-03 |
| AK026384 | potassium inwardly-rectifying channel, subfamily J, member 3 | Hs.199776 | 2q24.1 | KCNJ3 | 109 | 5 | -2.82 | 6.31E-04 | 2.57E-02 |
| NM_025206 | Fer-1-like 4 (C. elegans) |  |  |  | 187 | 13 | -2.81 | 2.80E-06 | 9.67E-04 |
| AK057539 | chromosome 21 open reading frame 108 | Hs.443257 | 21q22.11 | C21orf108 | 155 | 16 | -2.81 | 2.51E-04 | 1.47E-02 |
| NM_032136 | hypothetical protein DKFZp434L1717 | Hs.303923 | 4q32.2 | DKFZP434L1717 | 86 | 14 | -2.81 | 2.11E-03 | 4.98E-02 |
| AL034403 | Human DNA sequence from clone 31B8 on chromosome Xq22.2-23. Contains a GAPD (glyceraldehyde-3-phosph |  |  |  | 438 | 15 | -2.81 | 1.90E-04 | 1.27E-02 |
| AK024674 | Homo sapiens cDNA: FLJ21021 fis, clone CAE06069 |  |  |  | 250 | 8 | -2.81 | 3.26E-03 | 6.22E-02 |
| AL133656 | Homo sapiens, clone IMAGE:5266136, mRNA | Hs.127379 | 5q15 | SPATA9 | 79 | 10 | -2.80 | 2.95E-03 | 5.90E-02 |
| AK057144 | ribonuclease/angiogenin inhibitor | Hs.130958 | 11p15.5 | RNH | 509 | 16 | -2.79 | 1.19E-03 | 3.75E-02 |
| BC012155 | Homo sapiens, clone IMAGE:4561787, mRNA | Hs.344091 | 2q36.2 | DGAT2L1 | 244 | 16 | -2.79 | 5.41E-04 | 2.34E-02 |
| NM_014029 | HSPC022 protein |  |  |  | 1311 | 16 | -2.78 | 1.11E-07 | 1.08E-04 |
| NM_052944 | sodium/myo-inositol cotransporter 2 | Hs.164118 | 16pter-p11 | KST1 | 111 | 8 | -2.78 | 5.47E-03 | 7.87E-02 |
| NM_054030 | G protein-coupled receptor MRGX2 | Hs.350566 | 11p15.1 | MRGX2 | 193 | 9 | -2.78 | 4.30E-03 | 7.05E-02 |
| NM_018613 | Hypothetical protein PRO2007 |  |  |  | 83 | 5 | -2.78 | 2.57E-03 | 5.49E-02 |
| NM_001481 | chromosome 16 open reading frame 3 | Hs.431792 | 16q24.3 | C16orf3 | 1046 | 12 | -2.77 | 3.82E-05 | 5.12E-03 |
| NM_004864 | prostate differentiation factor | Hs.296638 | 19p13.1-13.2 | PLAB | 223 | 15 | -2.74 | 5.11E-04 | 2.27E-02 |
| NM_001259 | cyclin-dependent kinase 6 | Hs.38481 | 7q21-q22 | CDK6 | 267 | 8 | -2.74 | 1.60E-05 | 2.82E-03 |
| BC008442 | transmembrane 4 superfamily member 1 | Hs.351316 | 3q21-q25 | TM4SF1 | 420 | 13 | -2.74 | 1.27E-03 | 3.87E-02 |
| AL137669 | LYST-interacting protein LIP8 | Hs.348012 | 17p13.2 | LIP8 | 98 | 5 | -2.73 | 9.24E-04 | 3.25E-02 |
| AF332224 | chromosome Y open reading frame 15A | Hs.171857 | Yq11.222 | CYorf15A | 182 | 10 | -2.73 | 3.64E-03 | 6.53E-02 |
| NM_032289 | hypothetical protein DKFZp761B0514 | Hs.21963 | 5q31.3 | DKFZp761B0514 | 79 | 6 | -2.72 | 8.66E-03 | 9.88E-02 |
| AK057227 | Homo sapiens cDNA FLJ32665 fis, clone TESTI1000093 |  |  |  | 681 | 13 | -2.70 | 9.31E-04 | 3.26E-02 |
| NM_006637 | olfactory receptor, family 5, subfamily I, member 1 | Hs.159903 | 11q11 | OR5I1 | 244 | 14 | -2.70 | 2.28E-03 | 5.18E-02 |
| NM_004049 | BCL2-related protein A1 | Hs.227817 | 15q24.3 | BCL2A1 | 101 | 15 | -2.69 | 2.72E-03 | 5.66E-02 |
| NM_001078 | vascular cell adhesion molecule 1 | Hs.109225 | 1p32-p31 | VCAM1 | 120 | 12 | -2.68 | 4.85E-04 | 2.19E-02 |
| AF161341 | hypothetical protein LOC284367 | Hs.132045 | 19q13.41 | LOC284367 | 72 | 7 | -2.68 | 2.04E-03 | 4.90E-02 |
| NM_033035 | thymic stromal lymphopoietin | Hs.389874 | 5q22.2 | TSLP | 94 | 9 | -2.68 | 3.51E-04 | 1.83E-02 |
| NM_017954 | Ca2+-dependent activator protein for secretion 2 | Hs.489847 | 7q31.32 | CADPS2 | 78 | 6 | -2.66 | 4.37E-04 | 2.07E-02 |
| NM_016651 | dapper homolog 1, antagonist of beta-catenin (xenopus) | Hs.48950 | 14q23.1 | DACT1 | 89 | 8 | -2.66 | 2.85E-03 | 5.80E-02 |
| AK026784 | Homo sapiens cDNA: FLJ23131 fis, clone LNG08502 | Hs.301296 | 2q37.3 | FARP2 | 209 | 11 | -2.66 | 2.39E-04 | 1.43E-02 |
| NM_030576 | hypothetical protein MGC10986 | Hs.50601 | 17q24.2 | MGC10986 | 136 | 14 | -2.66 | 1.51E-04 | 1.14E-02 |
| NM_002910 | renin binding protein | Hs.158331 | Xq28 | RENBP | 140 | 8 | -2.66 | 8.11E-03 | 9.64E-02 |
| NM_024922 | esterase 31 | Hs.268700 | 16q22.1 | FLJ21736 | 179 | 11 | -2.64 | 1.44E-04 | 1.10E-02 |
| NM_031305 | hypothetical protein DKFZp564B1162 | Hs.442801 | 4q22.1 | DKFZP564B1162 | 99 | 15 | -2.64 | 3.13E-03 | 6.12E-02 |
| NM_000539 | rhodopsin (opsin 2, rod pigment) (retinitis pigmentosa 4, autosomal dominant) | Hs.247565 | 3q21-q24 | RHO | 152 | 7 | -2.64 | 5.57E-03 | 7.94E-02 |
| NM_002424 | matrix metalloproteinase 8 (neutrophil collagenase) | Hs.390002 | 11q22.3 | MMP8 | 393 | 16 | -2.64 | 3.04E-05 | 4.36E-03 |
| AK021839 | Homo sapiens cDNA FLJ11777 fis, clone HEMBA1005909. | Hs.147492 | 17q25 | DNAI2 | 153 | 12 | -2.63 | 1.22E-05 | 2.46E-03 |
| AK056626 | KIAA1244 protein | Hs.194408 | 6q23.3 | KIAA1244 | 72 | 7 | -2.63 | 1.24E-03 | 3.80E-02 |
| BC011762 | cytoplasmic FMR1 interacting protein 2 | Hs.211201 | 5q34 | CYFIP2 | 191 | 13 | -2.61 | 2.64E-03 | 5.56E-02 |
| AK024467 | hypothetical gene FLJ00060 | Hs.288520 | 19q13.42 | FLJ00060 | 102 | 5 | -2.61 | 1.98E-03 | 4.82E-02 |
| NM_012451 | synaptogyrin 4 | Hs.408333 | 19q13.3 | SYNGR4 | 113 | 8 | -2.61 | 4.12E-03 | 6.91E-02 |
| BC000737 | regulator of G-protein signalling 4 | Hs.386726 | 1q23.2 | RGS4 | 130 | 10 | -2.58 | 7.59E-05 | 7.56E-03 |
| NM_030751 | transcription factor 8 (represses interleukin 2 expression) | Hs.232068 | 10p11.2 | TCF8 | 141 | 12 | -2.57 | 4.93E-05 | 6.08E-03 |
| NM_022112 | p53-regulated apoptosis-inducing protein 1 | Hs.160953 | 11q24 | P53AIP1 | 134 | 13 | -2.57 | 4.29E-03 | 7.05E-02 |
| NM_000588 | interleukin 3 (colony-stimulating factor, multiple) | Hs.694 | 5q31.1 | IL3 | 348 | 13 | -2.56 | 8.36E-04 | 3.07E-02 |
| NM_001038 | sodium channel, nonvoltage-gated 1 alpha | Hs.446415 | 12p13 | SCNN1A | 115 | 7 | -2.56 | 3.24E-03 | 6.21E-02 |
| AB002338 | regulating synaptic membrane exocytosis 1 | Hs.302136 | 6q12-q13 | RIMS1 | 105 | 13 | -2.56 | 1.87E-03 | 4.68E-02 |
| AK027865 | Homo sapiens similar to zinc finger protein 433 (LOC339371), mRNA | Hs.127473 | 15q15.3 | MDS009 | 111 | 6 | -2.56 | 1.53E-04 | 1.13E-02 |
| NM_004613 | transglutaminase 2 (C polypeptide, protein-glutamine-gamma-glutamyltransferase) | Hs.512708 | 20q12 | TGM2 | 1914 | 16 | -2.55 | 2.28E-04 | 1.39E-02 |
| AK023647 | Homo sapiens cDNA FLJ13585 fis, clone PLACE1009150. | Hs.43047 | Xq22.1-q22.3 | RAB9B | 308 | 14 | -2.55 | 9.39E-04 | 3.25E-02 |
| NM_024536 | chondroitin polymerizing factor | Hs.458374 | 2q36.1 | CHPF | 1517 | 16 | -2.54 | 2.39E-05 | 3.79E-03 |
| AB047362 | neurexophilin 1 | Hs.343660 | 7p22 | NXPH1 | 334 | 16 | -2.53 | 2.58E-04 | 1.48E-02 |
| NM_001744 | calcium/calmodulin-dependent protein kinase IV | Hs.440638 | 5q21.3 | CAMK4 | 144 | 14 | -2.53 | 1.33E-04 | 1.07E-02 |
| AK056101 | Homo sapiens cDNA FLJ31539 fis, clone NT2RI2000738. | Hs.521260 | 8q24.12 | na | 321 | 16 | -2.53 | 5.78E-04 | 2.42E-02 |
| NM_014945 | KIAA0843 protein | Hs.282566 | 5q33.1 | KIAA0843 | 323 | 9 | -2.52 | 5.69E-04 | 2.43E-02 |
| BC008688 | zinc finger protein 85 (HPF4, HTF1) | Hs.37138 | 19p13.1-p12 | ZNF85 | 408 | 15 | -2.52 | 9.92E-04 | 3.38E-02 |
| NM_012452 | tumor necrosis factor receptor superfamily, member 13B | Hs.158341 | 17p11.2 | TNFRSF13B | 590 | 14 | -2.52 | 6.34E-03 | 8.47E-02 |
| NM_025266 | Hypothetical protein MGC2780 |  |  |  | 143 | 16 | -2.51 | 1.73E-03 | 4.54E-02 |
| J04178 | hexosaminidase A (alpha polypeptide) | Hs.411157 | 15q23-q24 | HEXA | 143 | 10 | -2.50 | 8.36E-03 | 9.72E-02 |
| NM_005284 | G protein-coupled receptor 6 | Hs.46332 | 6q21 | GPR6 | 333 | 15 | -2.50 | 7.19E-04 | 2.81E-02 |
| NM_004121 | gamma-glutamyltransferase-like activity 1 | Hs.437156 | 22q11.23 | GGTLA1 | 176 | 8 | -2.50 | 9.80E-04 | 3.35E-02 |
| NM_016293 | bridging integrator 2 | Hs.14770 | 12q13 | BIN2 | 136 | 5 | -2.49 | 6.06E-04 | 2.50E-02 |
| NM_003543 | histone 1, H4h | Hs.421737 | 6p21.3 | HIST1H4H | 351 | 16 | -2.49 | 1.33E-04 | 1.07E-02 |
| AK024569 | hypothetical protein FLJ21034 | Hs.282466 | 8p21.2 | FLJ21034 | 306 | 15 | -2.48 | 3.81E-04 | 1.88E-02 |
| NM_020217 | spectrin, beta, non-erythrocytic 1 | Hs.205401 | 2p21 | SPTBN1 | 94 | 10 | -2.48 | 7.35E-03 | 9.23E-02 |
| AK026743 | Homo sapiens cDNA: FLJ23090 fis, clone LNG07119 | Hs.306875 | 11q23.2 | KIAA0781 | 95 | 12 | -2.45 | 2.27E-03 | 5.18E-02 |
| BC017978 | Homo sapiens mRNA; cDNA DKFZp779A2267 (from clone DKFZp779A2267) | Hs.351798 | 15q22.31 | NOX5 | 146 | 14 | -2.45 | 2.77E-03 | 5.72E-02 |
| NM_021730 | Hypothetical protein PP1044 |  |  |  | 168 | 9 | -2.44 | 3.71E-03 | 6.58E-02 |
| NM_018173 | hypothetical protein FLJ10665 | Hs.163953 | 12p13.32 | FLJ10665 | 192 | 10 | -2.43 | 3.52E-03 | 6.44E-02 |
| NM_003880 | WNT1 inducible signaling pathway protein 3 | Hs.194678 | 6q22-q23 | WISP3 | 139 | 16 | -2.43 | 2.10E-03 | 4.98E-02 |
| NM_022119 | protease, serine, 22 | Hs.125532 | 16p13.3 | PRSS22 | 190 | 12 | -2.42 | 8.03E-03 | 9.61E-02 |
| AL137659 | apical early endosomal glycoprotein precursor | Hs.376780 | 9q34.3 | AEGP | 609 | 11 | -2.42 | 1.02E-04 | 8.90E-03 |
| NM_004598 | sparc/osteonectin, cwcv and kazal-like domains proteoglycan (testican) | Hs.93029 | 5q31 | SPOCK | 619 | 15 | -2.40 | 5.37E-04 | 2.33E-02 |
| NM_000214 | jagged 1 (Alagille syndrome) | Hs.409202 | 20p12.1-p11.23 | JAG1 | 460 | 16 | -2.40 | 3.99E-05 | 5.21E-03 |
| AL117382 | R3H domain (binds single-stranded nucleic acids) containing-like |  |  |  | 75 | 11 | -2.40 | 3.29E-03 | 6.22E-02 |
| NM_001524 | hypocretin (orexin) neuropeptide precursor | Hs.158348 | 17q21 | HCRT | 313 | 14 | -2.40 | 1.03E-03 | 3.46E-02 |
| NM_024871 | hypothetical protein FLJ12748 | Hs.203013 | 3q27.3 | FLJ12748 | 104 | 11 | -2.40 | 1.70E-03 | 4.50E-02 |
| D43968 | runt-related transcription factor 1 (acute myeloid leukemia 1; aml1 oncogene) | Hs.410774 | 21q22.3 | RUNX1 | 350 | 16 | -2.40 | 1.38E-05 | 2.68E-03 |
| AK056624 | NEDD8 ultimate buster-1 | Hs.182454 | 7q36 | NYREN18 | 178 | 16 | -2.39 | 8.24E-03 | 9.68E-02 |
| NM_031456 | chromosome 17 open reading frame 1A | Hs.158313 | 17p12 | C17orf1A | 2389 | 14 | -2.39 | 6.49E-05 | 7.10E-03 |
| AK026757 | erbb2 interacting protein | Hs.8117 | 5q13.1 | ERBB2IP | 156 | 15 | -2.38 | 2.54E-04 | 1.48E-02 |
| NM_006006 | zinc finger protein 145 (Kruppel-like, expressed in promyelocytic leukemia) | Hs.37096 | 11q23.1 | ZNF145 | 244 | 14 | -2.38 | 3.00E-03 | 5.95E-02 |
| NM_004479 | fucosyltransferase 7 (alpha (1,3) fucosyltransferase) | Hs.457 | 9q34.3 | FUT7 | 140 | 9 | -2.38 | 6.09E-03 | 8.33E-02 |
| BC001665 | KIAA0843 protein | Hs.282566 | 5q33.1 | KIAA0843 | 67 | 8 | -2.38 | 1.24E-03 | 3.80E-02 |
| D13628 | angiopoietin 1 | Hs.2463 | 8q22.3-q23 | ANGPT1 | 118 | 5 | -2.37 | 2.06E-04 | 1.30E-02 |
| NM_000396 | cathepsin K (pycnodysostosis) | Hs.83942 | 1q21 | CTSK | 551 | 16 | -2.37 | 6.64E-06 | 1.69E-03 |
| NM_002707 | protein phosphatase 1G (formerly 2C), magnesium-dependent, gamma isoform | Hs.17883 | 2p23.3 | PPM1G | 101 | 6 | -2.36 | 4.55E-03 | 7.22E-02 |
| AK057127 | RALBP1 associated Eps domain containing 2 | Hs.334168 | Xp22.22 | REPS2 | 132 | 16 | -2.36 | 1.19E-03 | 3.75E-02 |
| BC017085 | tumor differentially expressed protein 2 | Hs.443695 | 1p35.1 | TDE2 | 828 | 15 | -2.35 | 1.53E-04 | 1.13E-02 |
| BC016981 | Homo sapiens, Similar to nuclear localization signals binding protein 1, clone MGC:21810 IMAGE:4183576, mRNA, complete cds | Hs.244624 | 3p25.2 | KIAA0763 | 129 | 13 | -2.35 | 5.11E-03 | 7.58E-02 |
| NM_001945 | diphtheria toxin receptor (heparin-binding epidermal growth factor-like growth factor) | Hs.799 | 5q23 | DTR | 211 | 8 | -2.35 | 2.35E-03 | 5.28E-02 |
| NM_003784 | serine (or cysteine) proteinase inhibitor, clade B (ovalbumin), member 7 | Hs.138202 | 18q22.1 | SERPINB7 | 104 | 11 | -2.35 | 5.92E-03 | 8.22E-02 |
| NM_012344 | neurotensin receptor 2 | Hs.131138 | 2p25.1 | NTSR2 | 114 | 8 | -2.34 | 3.56E-03 | 6.44E-02 |
| NM_024695 | Hypothetical protein FLJ13993 |  |  |  | 204 | 16 | -2.34 | 1.74E-03 | 4.56E-02 |
| AB036063 | ribonucleotide reductase M2 B (TP53 inducible) | Hs.512592 | 8q23.1 | RRM2B | 400 | 16 | -2.34 | 3.22E-05 | 4.58E-03 |
| NM_013389 | NPC1 (Niemann-Pick disease, type C1, gene)-like 1 | Hs.512147 | 7p13 | NPC1L1 | 125 | 11 | -2.33 | 3.28E-03 | 6.23E-02 |
| AK001604 | Homo sapiens cDNA FLJ10742 fis, clone NT2RP3001629. | Hs.480800 | 12q13 | RPS26 | 245 | 13 | -2.33 | 2.79E-04 | 1.59E-02 |
| BC010942 | acetyl-Coenzyme A acetyltransferase 1 (acetoacetyl Coenzyme A thiolase) | Hs.37 | 11q22.3-q23.1 | ACAT1 | 249 | 15 | -2.33 | 1.84E-03 | 4.66E-02 |
| NM_001993 | coagulation factor III (thromboplastin, tissue factor) | Hs.62192 | 1p22-p21 | F3 | 170 | 15 | -2.32 | 6.92E-05 | 7.36E-03 |
| AF113009 | hypothetical gene CG018 | Hs.277888 | 13q12-q13 | CG018 | 502 | 14 | -2.32 | 3.35E-03 | 6.28E-02 |
| NM_002191 | inhibin, alpha | Hs.407506 | 2q33-q36 | INHA | 136 | 9 | -2.32 | 5.46E-03 | 7.87E-02 |
| NM_001742 | calcitonin receptor | Hs.640 | 7q21.3 | CALCR | 165 | 14 | -2.32 | 1.00E-03 | 3.39E-02 |
| NM_018004 | hypothetical protein FLJ10134 | Hs.104800 | 3q12.3 | FLJ10134 | 640 | 14 | -2.32 | 8.45E-05 | 7.91E-03 |
| NM_001612 | acrosomal vesicle protein 1 | Hs.169222 | 11p12-q13 | ACRV1 | 354 | 16 | -2.30 | 3.78E-03 | 6.63E-02 |
| NM_032764 | hypothetical protein MGC16153 | Hs.279627 | 9p24.2 | MGC16153 | 144 | 8 | -2.29 | 2.23E-04 | 1.38E-02 |
| NM_003494 | dysferlin, limb girdle muscular dystrophy 2B (autosomal recessive) | Hs.1432 | 2p13.3-p13.1 | DYSF | 164 | 13 | -2.28 | 3.71E-04 | 1.85E-02 |
| NM_031289 | germ cell associated 1 | Hs.132337 | 12p13.2 | GSG1 | 73 | 12 | -2.28 | 1.69E-03 | 4.48E-02 |
| AK022074 | rap2 interacting protein x | Hs.7972 | 4q21.1 | RIPX | 299 | 16 | -2.28 | 1.12E-03 | 3.60E-02 |
| NM_003236 | transforming growth factor, alpha | Hs.170009 | 2p13 | TGFA | 792 | 16 | -2.28 | 2.90E-03 | 5.83E-02 |
| AK026112 | hypothetical protein MGC4606 | Hs.330418 | 16p12.1 | MGC4606 | 347 | 15 | -2.28 | 8.09E-04 | 3.01E-02 |
| AB051432 | Guanine nucleotide binding protein (G protein), beta polypeptide 1-like |  |  |  | 388 | 15 | -2.26 | 2.48E-03 | 5.41E-02 |
| AK022045 | Homo sapiens cDNA FLJ11983 fis, clone HEMBB1001337. | Hs.435032 | 2q37.3 | ANKMY1 | 151 | 15 | -2.26 | 1.23E-03 | 3.79E-02 |
| NM_005267 | gap junction protein, alpha 8, 50kDa (connexin 50) | Hs.157433 | 1q21.1 | GJA8 | 110 | 13 | -2.26 | 6.84E-03 | 8.86E-02 |
| AK024973 | Homo sapiens cDNA: FLJ21320 fis, clone COL02324 | Hs.306737 | 12p13.3 | ELKS | 88 | 16 | -2.26 | 2.16E-03 | 5.05E-02 |
| AY013295 | myozenin 2 | Hs.381047 | 4q26-q27 | MYOZ2 | 134 | 12 | -2.26 | 1.59E-03 | 4.33E-02 |
| AL133404 | Human DNA sequence from clone RP1-238O23 on chromosome 6. Contains part of the gene for a novel prot |  |  |  | 206 | 10 | -2.26 | 1.46E-03 | 4.18E-02 |
| AB067491 | KIAA1904 protein | Hs.250726 | 22q13.1 | KIAA1904 | 680 | 16 | -2.25 | 4.19E-04 | 2.03E-02 |
| BC014941 | inhibitor of DNA binding 4, dominant negative helix-loop-helix protein | Hs.391392 | 6p22-p21 | ID4 | 355 | 15 | -2.25 | 8.01E-04 | 2.99E-02 |
| NM_024979 | MCF.2 cell line derived transforming sequence-like | Hs.436905 | 13q34 | MCF2L | 96 | 10 | -2.25 | 2.91E-03 | 5.84E-02 |
| AK024108 | Homo sapiens cDNA FLJ14046 fis, clone HEMBA1006461. | Hs.444689 | 19q13.33 | na | 134 | 6 | -2.24 | 2.15E-03 | 5.03E-02 |
| BC001607 | Homo sapiens similar to Nonhistone chromosomal protein HMG-17 (High-mobility group nucleosome binding domain 2) (LOC350435), mRNA | Hs.322444 | 17p13.1 | NEUROD2 | 470 | 11 | -2.24 | 1.55E-03 | 4.28E-02 |
| NM_005319 | histone 1, H1c | Hs.7644 | 6p21.3 | HIST1H1C | 888 | 10 | -2.24 | 8.97E-03 | 9.99E-02 |
| AL049385 | kelch-like 5 (Drosophila) | Hs.272251 | 4p14 | KLHL5 | 170 | 7 | -2.23 | 5.65E-03 | 8.00E-02 |
| NM_020639 | ankyrin repeat domain 3 | Hs.55565 | 21q22.3 | ANKRD3 | 1220 | 16 | -2.23 | 5.35E-06 | 1.48E-03 |
| AF022375 | vascular endothelial growth factor | Hs.73793 | 6p12 | VEGF | 1163 | 16 | -2.23 | 1.53E-03 | 4.27E-02 |
| M37435 | Colony stimulating factor 1 (macrophage) |  |  |  | 534 | 16 | -2.23 | 1.43E-03 | 4.14E-02 |
| NM_025216 | wingless-type MMTV integration site family, member 10A | Hs.121540 | 2q35 | WNT10A | 78 | 8 | -2.23 | 7.37E-03 | 9.23E-02 |
| AK056665 | plasticity related gene 1 | Hs.13245 | 1p21.3 | PRG1 | 493 | 15 | -2.22 | 1.93E-04 | 1.27E-02 |
| AF155103 | ankyrin repeat domain 13 | Hs.122764 | 12q24.12 | ANKRD13 | 1273 | 14 | -2.22 | 2.99E-05 | 4.38E-03 |
| AL117598 | Homo sapiens, clone IMAGE:5312754, mRNA | Hs.137206 | 15q15-q21 | TLN2 | 146 | 9 | -2.22 | 1.35E-04 | 1.07E-02 |
| NM_004817 | tight junction protein 2 (zona occludens 2) | Hs.75608 | 9q13-q21 | TJP2 | 1047 | 16 | -2.22 | 3.64E-04 | 1.85E-02 |
| AK056150 | hypothetical protein LOC149478 | Hs.421430 | 1p34.1 | LOC149478 | 123 | 14 | -2.22 | 1.00E-03 | 3.40E-02 |
| NM_000203 | iduronidase, alpha-L- | Hs.89560 | 4p16.3 | IDUA | 98 | 10 | -2.22 | 1.77E-03 | 4.61E-02 |
| NM_000558 | hemoglobin, alpha 1 | Hs.449630 | 16p13.3 | HBA1 | 157 | 9 | -2.22 | 2.98E-03 | 5.93E-02 |
| AB037848 | synaptotagmin XIII | Hs.12365 | 11p12-p11 | SYT13 | 320 | 16 | -2.21 | 7.46E-05 | 7.53E-03 |
| NM_000039 | apolipoprotein A-I | Hs.93194 | 11q23-q24 | APOA1 | 118 | 11 | -2.21 | 4.95E-03 | 7.45E-02 |
| AK056521 | hypothetical protein MGC23427 | Hs.244847 | 9q34.3 | MGC23427 | 1447 | 16 | -2.21 | 5.46E-05 | 6.43E-03 |
| NM_022740 | homeodomain interacting protein kinase 2 | Hs.397465 | 7q32-q34 | HIPK2 | 237 | 10 | -2.20 | 3.39E-03 | 6.31E-02 |
| AK055539 | layilin | Hs.317614 | 11q23.2 | LOC143903 | 323 | 8 | -2.20 | 1.28E-03 | 3.88E-02 |
| NM_018017 | CTCL tumor antigen L14-2 | Hs.159066 | 10q26.11 | FLJ10188 | 170 | 16 | -2.20 | 1.32E-04 | 1.06E-02 |
| BC011575 | kidney predominant protein NCU-G1 | Hs.202522 | 1q23.1 | MGC31963 | 164 | 15 | -2.20 | 8.72E-03 | 9.89E-02 |
| NM_025023 | chromosome 17 open reading frame 31 | Hs.448342 | 17p13.3 | C17orf31 | 77 | 16 | -2.20 | 2.58E-03 | 5.50E-02 |
| NM_018488 | T-box 4 | Hs.143907 | 17q21-q22 | TBX4 | 118 | 14 | -2.20 | 1.06E-03 | 3.51E-02 |
| AB058704 | hypothetical protein FLJ12303 | Hs.402200 | 12q21 | FLJ12303 | 112 | 14 | -2.20 | 1.82E-03 | 4.67E-02 |
| NM_018295 | hypothetical protein FLJ11000 | Hs.407831 | 7q33 | FLJ11000 | 68 | 14 | -2.19 | 6.13E-03 | 8.35E-02 |
| AK023818 | Homo sapiens cDNA FLJ13756 fis, clone PLACE3000365. | Hs.447459 | 10q24.32 | C10orf6 | 495 | 11 | -2.19 | 7.05E-05 | 7.44E-03 |
| NM_001381 | docking protein 1, 62kDa (downstream of tyrosine kinase 1) | Hs.103854 | 2p13 | DOK1 | 314 | 16 | -2.19 | 4.40E-04 | 2.06E-02 |
| AK023187 | G-rich RNA sequence binding factor 1 | Hs.309763 | 4q13 | GRSF1 | 319 | 16 | -2.19 | 1.95E-03 | 4.80E-02 |
| NM_004995 | matrix metalloproteinase 14 (membrane-inserted) | Hs.2399 | 14q11-q12 | MMP14 | 357 | 10 | -2.19 | 1.05E-03 | 3.49E-02 |
| NM_001233 | caveolin 2 | Hs.139851 | 7q31.1 | CAV2 | 1972 | 16 | -2.19 | 7.02E-06 | 1.76E-03 |
| BF026507 | RPEL repeat containing 1 | Hs.191979 | 6p23 | RPEL1 | 278 | 14 | -2.19 | 1.08E-04 | 9.14E-03 |
| AK057158 | Homo sapiens cDNA FLJ32596 fis, clone SPLEN2000515. | Hs.492554 | 8q13.3 | na | 115 | 14 | -2.18 | 2.29E-03 | 5.18E-02 |
| NM_003596 | tyrosylprotein sulfotransferase 1 | Hs.421194 | 7q11.21 | TPST1 | 1273 | 16 | -2.18 | 1.41E-05 | 2.70E-03 |
| D87942 | Homo sapiens cDNA clone MGC:2062 IMAGE:3534501, complete cds | Hs.421280 | 8q13.3 | RPL7 | 276 | 16 | -2.18 | 6.73E-03 | 8.81E-02 |
| NM_020482 | activator of cAMP-responsive element modulator (CREM) in testis | Hs.283689 | 6q16.1-q16.3 | ACT | 125 | 15 | -2.18 | 2.38E-03 | 5.30E-02 |
| NM_003648 | diacylglycerol kinase, delta 130kDa | Hs.115907 | 2q37.1 | DGKD | 1259 | 16 | -2.17 | 4.28E-06 | 1.30E-03 |
| NM_000353 | tyrosine aminotransferase | Hs.161640 | 16q22.1 | TAT | 181 | 16 | -2.17 | 6.45E-03 | 8.58E-02 |
| AJ223366 | myeloma overexpressed gene (in a subset of t(11;14) positive multiple myelomas) | Hs.436000 | 11q13 | MYEOV | 167 | 8 | -2.17 | 1.69E-04 | 1.19E-02 |
| NM_019058 | HIF-1 responsive RTP801 | Hs.111244 | 10pter-q26.12 | RTP801 | 254 | 14 | -2.17 | 1.91E-03 | 4.74E-02 |
| AK023632 | Homo sapiens cDNA FLJ13570 fis, clone PLACE1008392. | Hs.468742 | 1p36 | T1A-2 | 160 | 16 | -2.17 | 3.29E-04 | 1.76E-02 |
| AF116655 | Homo sapiens PRO1082 mRNA, complete cds |  |  |  | 184 | 14 | -2.17 | 3.91E-04 | 1.91E-02 |
| AF444779 | spectrin repeat containing, nuclear envelope 1 | Hs.282117 | 6q25 | SYNE1 | 264 | 13 | -2.16 | 4.33E-04 | 2.06E-02 |
| BC006428 | hypothetical protein HSPC195 | Hs.356509 | 5q31.3 | HSPC195 | 2097 | 12 | -2.16 | 2.43E-04 | 1.44E-02 |
| NM_012324 | mitogen-activated protein kinase 8 interacting protein 2 | Hs.356523 | 22q13.33 | MAPK8IP2 | 191 | 13 | -2.16 | 6.31E-03 | 8.45E-02 |
| AB015349 | hypothetical protein LOC150946 | Hs.187912 | 2p24.1 | LOC150946 | 314 | 15 | -2.16 | 4.32E-03 | 7.06E-02 |
| AL049296 | likely ortholog of mouse nervous system polycomb 1 | Hs.316750 | 2p13.1 | NSPC1 | 138 | 16 | -2.16 | 3.55E-03 | 6.44E-02 |
| NM_012447 | stromal antigen 3 | Hs.323634 | 7q22.1 | STAG3 | 196 | 11 | -2.16 | 8.65E-03 | 9.86E-02 |
| NM_032553 | putative purinergic receptor | Hs.326713 | Xq13.3 | FKSG79 | 170 | 16 | -2.16 | 1.18E-03 | 3.73E-02 |
| NM_000914 | opioid receptor, mu 1 | Hs.2353 | 6q24-q25 | OPRM1 | 131 | 10 | -2.16 | 3.63E-03 | 6.51E-02 |
| NM_033641 | collagen, type IV, alpha 6 | Hs.408 | Xq22 | COL4A6 | 542 | 16 | -2.15 | 2.33E-04 | 1.40E-02 |
| NM_000681 | adrenergic, alpha-2A-, receptor | Hs.249159 | 10q24-q26 | ADRA2A | 376 | 15 | -2.15 | 1.92E-03 | 4.74E-02 |
| NM_007021 | decidual protein induced by progesterone | Hs.93675 | 10q11.21 | DEPP | 329 | 15 | -2.15 | 2.49E-05 | 3.91E-03 |
| AL359567 | Homo sapiens mRNA; cDNA DKFZp547D023 (from clone DKFZp547D023) | Hs.161962 | 11q24 | MMP27 | 187 | 16 | -2.15 | 1.47E-03 | 4.18E-02 |
| AF305616 | transmembrane, prostate androgen induced RNA | Hs.83883 | 20q13.31-q13.33 | TMEPAI | 2229 | 16 | -2.15 | 2.82E-05 | 4.21E-03 |
| NM_007233 | TP53 activated protein 1 | Hs.274329 | 7q21.1 | TP53AP1 | 266 | 14 | -2.15 | 4.73E-04 | 2.17E-02 |
| NM_024898 | hypothetical protein FLJ22757 | Hs.236449 | 19p13.3 | FLJ22757 | 180 | 11 | -2.14 | 2.40E-03 | 5.32E-02 |
| NM_018898 | protocadherin alpha 3 | Hs.247734 | 5q31 | PCDHA3 | 96 | 7 | -2.14 | 1.87E-03 | 4.68E-02 |
| NM_003436 | zinc finger protein 135 (clone pHZ-17) | Hs.85863 | 19q13.4 | ZNF135 | 136 | 16 | -2.14 | 2.39E-03 | 5.31E-02 |
| AK057995 | hypothetical protein FLJ12847 | Hs.212991 | 8p23.1 | FLJ12847 | 105 | 12 | -2.14 | 2.26E-04 | 1.39E-02 |
| NM_018203 | hypothetical protein FLJ10748 | Hs.10414 | 1q32.1 | FLJ10748 | 250 | 14 | -2.14 | 8.42E-03 | 9.73E-02 |
| NM_024980 | hypothetical protein FLJ12132 | Hs.287490 | 1p36.22 | FLJ12132 | 728 | 13 | -2.13 | 4.66E-03 | 7.32E-02 |
| AF038190 | hypothetical protein DKFZp761N09121 | Hs.6421 | 7 | DKFZP761N09121 | 160 | 7 | -2.13 | 2.85E-03 | 5.80E-02 |
| AF220217 | SEC15-like 1 (S. cerevisiae) | Hs.272374 | 10q23.33 | SEC15L1 | 107 | 9 | -2.13 | 2.37E-03 | 5.29E-02 |
| X66436 | guanine nucleotide binding protein-like 1 | Hs.83147 | 6p21.3 | GNL1 | 67 | 7 | -2.13 | 8.83E-03 | 9.95E-02 |
| AK001132 | ankyrin 3, node of Ranvier (ankyrin G) | Hs.440478 | 10q21 | ANK3 | 320 | 16 | -2.13 | 4.27E-03 | 7.03E-02 |
| NM_032561 | chromosome 22 open reading frame 23 | Hs.111726 | 22q13.1 | C22orf23 | 184 | 10 | -2.13 | 2.03E-03 | 4.90E-02 |
| NM_002148 | homeo box D10 | Hs.123070 | 2q31.1 | HOXD10 | 135 | 13 | -2.12 | 4.99E-03 | 7.47E-02 |
| AL137541 | Homo sapiens mRNA; cDNA DKFZp434O0919 (from clone DKFZp434O0919) | Hs.306471 | 22q11.21 | DGCR14 | 227 | 16 | -2.12 | 3.54E-03 | 6.44E-02 |
| AK021468 | Homo sapiens cDNA FLJ11406 fis, clone HEMBA1000773. | Hs.287412 | 10q26.3 | FLJ11370 | 90 | 16 | -2.12 | 3.19E-03 | 6.18E-02 |
| NM_018696 | elaC homolog 1 (E. coli) | Hs.47572 | 18q21 | ELAC1 | 247 | 15 | -2.12 | 3.18E-03 | 6.16E-02 |
| NM_001506 | G protein-coupled receptor 32 | Hs.248125 | 19q13.3 | GPR32 | 272 | 16 | -2.11 | 4.94E-03 | 7.47E-02 |
| NM_025043 | Hypothetical protein FLJ22404 |  |  |  | 142 | 9 | -2.11 | 4.60E-03 | 7.27E-02 |
| NM_006174 | neuropeptide Y receptor Y5 | Hs.158330 | 4q31-q32 | NPY5R | 169 | 15 | -2.11 | 5.25E-03 | 7.67E-02 |
| AF230412 | tripartite motif-containing 34 | Hs.125300 | 11p15 | TRIM34 | 227 | 15 | -2.11 | 2.11E-03 | 4.98E-02 |
| NM_017795 | Hypothetical protein FLJ20378 |  |  |  | 188 | 16 | -2.11 | 3.34E-03 | 6.26E-02 |
| NM_000710 | bradykinin receptor B1 | Hs.46348 | 14q32.1-q32.2 | BDKRB1 | 278 | 16 | -2.09 | 1.21E-03 | 3.77E-02 |
| NM_014391 | cardiac ankyrin repeat protein | Hs.448589 | 10q23.33 | CARP | 377 | 16 | -2.09 | 2.40E-03 | 5.33E-02 |
| NM_033547 | hypothetical gene MGC16733 similar to CG12113 | Hs.141245 | 11q13.4 | MGC16733 | 113 | 5 | -2.09 | 8.26E-03 | 9.68E-02 |
| AK025225 | Homo sapiens cDNA: FLJ21572 fis, clone COL06651 | Hs.159316 | 8p11.2-p11.1 | DKK4 | 1150 | 16 | -2.09 | 1.18E-03 | 3.73E-02 |
| AB037794 | associated molecule with the SH3 domain of STAM (AMSH) like protein | Hs.16229 | 10q23.32 | AMSH-LP | 200 | 9 | -2.09 | 4.11E-03 | 6.89E-02 |
| AY026052 | cat eye syndrome chromosome region, candidate 7 | Hs.125761 | 12q22 | CECR7 | 133 | 16 | -2.08 | 1.17E-03 | 3.73E-02 |
| NM_018654 | G protein-coupled receptor, family C, group 5, member D | Hs.283073 | 12p13.3 | GPRC5D | 239 | 14 | -2.08 | 2.32E-03 | 5.22E-02 |
| NM_025261 | lymphocyte antigen 6 complex, locus G6C | Hs.241586 | 6p21.31 | LY6G6C | 153 | 16 | -2.08 | 4.71E-03 | 7.34E-02 |
| AK021480 | Homo sapiens cDNA FLJ11418 fis, clone HEMBA1000972. | Hs.296647 | 2q24.3 | KCNH7 | 107 | 16 | -2.08 | 1.04E-03 | 3.48E-02 |
| AK056067 | Homo sapiens cDNA FLJ31505 fis, clone NT2NE2005821. | Hs.517910 | 3p21.31 | MGC39725 | 269 | 14 | -2.07 | 3.48E-03 | 6.38E-02 |
| NM_018265 | hypothetical protein FLJ10901 | Hs.73239 | 1q32.1 | FLJ10901 | 383 | 14 | -2.07 | 1.78E-03 | 4.62E-02 |
| NM_014279 | olfactomedin 1 | Hs.74376 | 9q34.3 | OLFM1 | 149 | 15 | -2.07 | 6.90E-03 | 8.91E-02 |
| AB040878 | sema domain, seven thrombospondin repeats (type 1 and type 1-like), transmembrane domain (TM) and short cytoplasmic domain, (semaphorin) 5B | Hs.61384 | 3q21.1 | SEMA5B | 161 | 16 | -2.07 | 1.78E-03 | 4.62E-02 |
| AF131821 | Homo sapiens monoglyceride lipase, mRNA (cDNA clone IMAGE:4441572), partial cds | Hs.518233 | 3q21-qter | PLXNA1 | 1511 | 16 | -2.07 | 1.01E-04 | 8.89E-03 |
| NM_032326 | hypothetical protein MGC4618 | Hs.89072 | 4p16.3 | MGC4618 | 413 | 15 | -2.07 | 9.68E-05 | 8.69E-03 |
| NM_005663 | Wolf-Hirschhorn syndrome candidate 2 | Hs.21771 | 4p16.3 | WHSC2 | 948 | 15 | -2.07 | 1.58E-03 | 4.32E-02 |
| NM_007064 | serine/threonine kinase with Dbl- and pleckstrin homology domains | Hs.162189 | 3q21.2 | TRAD | 234 | 16 | -2.07 | 2.86E-03 | 5.80E-02 |
| AF035291 | Homo sapiens clone 23571 and 23784 mRNA sequence | Hs.113744 | 15q26 | SLC21A11 | 206 | 15 | -2.07 | 6.76E-04 | 2.68E-02 |
| AF052172 | Homo sapiens clone 24617 mRNA sequence | Hs.64125 | 18q21.1 | KIAA0427 | 187 | 15 | -2.07 | 5.70E-03 | 8.03E-02 |
| BC011266 | hypothetical protein BC011266 | Hs.65918 | 3q26.31 | LOC93556 | 337 | 16 | -2.07 | 4.48E-06 | 1.34E-03 |
| NM_025025 | hypothetical protein FLJ14100 | Hs.287623 | 1p36.32 | FLJ14100 | 123 | 15 | -2.07 | 4.72E-03 | 7.35E-02 |
| NM_018555 | zinc finger protein 331 | Hs.147644 | 19q13.3-q13.4 | ZNF331 | 375 | 14 | -2.06 | 1.80E-04 | 1.24E-02 |
| NM_006681 | neuromedin U | Hs.418367 | 4q12 | NMU | 252 | 15 | -2.06 | 8.03E-03 | 9.62E-02 |
| AL137339 | hypothetical protein LOC149401 | Hs.306452 | 1q32.1 | LOC149401 | 408 | 16 | -2.06 | 4.75E-03 | 7.35E-02 |
| AL117587 | KIAA1281 protein | Hs.93738 | 5q23.2 | KIAA1281 | 1902 | 16 | -2.06 | 1.01E-05 | 2.18E-03 |
| NM_014553 | LBP protein; likely ortholog of mouse CRTR-1 | Hs.119903 | 2q14 | LBP-9 | 212 | 13 | -2.06 | 5.06E-03 | 7.54E-02 |
| AK027183 | Homo sapiens cDNA: FLJ23530 fis, clone LNG06055 | Hs.287744 | 19p13.3 | FLJ23420 | 195 | 15 | -2.06 | 5.10E-03 | 7.58E-02 |
| AK054981 | Munc13-3 | Hs.112921 | 15q21.1 | DKFZp547H074 | 93 | 7 | -2.06 | 3.36E-03 | 6.29E-02 |
| AL133017 | hypothetical protein FLJ22865 | Hs.302051 | 17q21.1 | FLJ22865 | 89 | 9 | -2.06 | 8.07E-03 | 9.63E-02 |
| NM_000689 | aldehyde dehydrogenase 1 family, member A1 | Hs.76392 | 9q21.13 | ALDH1A1 | 924 | 16 | -2.06 | 1.17E-03 | 3.73E-02 |
| AK055955 | seven transmembrane helix receptor | Hs.350816 | 11q12.1 | FLJ31393 | 271 | 16 | -2.06 | 2.05E-03 | 4.89E-02 |
| BC016964 | G protein-coupled receptor MrgF | Hs.118513 | 11q13.1 | MGC21621 | 120 | 10 | -2.05 | 7.80E-03 | 9.47E-02 |
| AK000691 | chromosome 6 open reading frame 85 | Hs.7734 | 6p25.2 | C6orf85 | 699 | 16 | -2.05 | 1.79E-03 | 4.63E-02 |
| NM_031938 | beta-carotene dioxygenase 2 | Hs.224505 | 11q22.3-q23.1 | BCDO2 | 184 | 16 | -2.05 | 2.92E-03 | 5.86E-02 |
| NM_003656 | calcium/calmodulin-dependent protein kinase I | Hs.512804 | 3p25.3 | CAMK1 | 778 | 16 | -2.05 | 2.17E-05 | 3.47E-03 |
| AK022165 | Homo sapiens cDNA FLJ12103 fis, clone HEMBB1002692. | Hs.464492 | 17q25.3 | LOC146713 | 109 | 16 | -2.05 | 5.33E-03 | 7.76E-02 |
| NM_016097 | HSPC039 protein | Hs.406542 | 18q12 | HSPC039 | 1132 | 16 | -2.05 | 4.75E-04 | 2.17E-02 |
| NM_002031 | fyn-related kinase | Hs.89426 | 6q21-q22.3 | FRK | 153 | 11 | -2.05 | 7.87E-05 | 7.78E-03 |
| NM_002760 | protein kinase, Y-linked | Hs.183165 | Yp11.2 | PRKY | 158 | 6 | -2.05 | 2.58E-03 | 5.50E-02 |
| NM_024724 | Hypothetical protein FLJ22332 |  |  |  | 207 | 13 | -2.05 | 1.64E-03 | 4.42E-02 |
| AK057511 | hypothetical protein FLJ32949 | Hs.484250 | 12q14.1 | FLJ32949 | 98 | 11 | -2.05 | 4.04E-03 | 6.85E-02 |
| BC013920 | Homo sapiens, clone IMAGE:4829271, mRNA | Hs.301711 | 9p13.2 | FLJ11560 | 1214 | 16 | -2.05 | 1.56E-04 | 1.14E-02 |
| NM_003199 | transcription factor 4 | Hs.359289 | 18q21.1 | TCF4 | 191 | 14 | -2.04 | 6.03E-03 | 8.30E-02 |
| AF052090 | nicotinamide nucleotide transhydrogenase | Hs.106620 | 5p13.1-5cen | NNT | 122 | 5 | -2.04 | 1.01E-03 | 3.41E-02 |
| NM_018404 | centaurin, alpha 2 | Hs.415471 | 17q11.2 | CENTA2 | 226 | 9 | -2.04 | 4.76E-03 | 7.34E-02 |
| AK023427 | Homo sapiens cDNA FLJ13365 fis, clone PLACE1000332. | Hs.306646 | 17q11.2 | DKFZp667M2411 | 310 | 16 | -2.04 | 2.19E-03 | 5.08E-02 |
| NM_032126 | hypothetical protein DKFZp564J047 | Hs.369418 | 1q24.3 | DKFZP564J047 | 119 | 12 | -2.04 | 4.95E-03 | 7.46E-02 |
| NM_002073 | guanine nucleotide binding protein (G protein), alpha z polypeptide | Hs.437081 | 22q11.22 | GNAZ | 250 | 12 | -2.04 | 1.91E-04 | 1.27E-02 |
| AB011145 | thioredoxin domain containing 4 (endoplasmic reticulum) | Hs.154023 | 9q31.1 | TXNDC4 | 253 | 12 | -2.04 | 7.87E-04 | 2.99E-02 |
| AK025205 | DKFZP564O0823 protein | Hs.105460 | 4q13.3-q21.3 | DKFZP564O0823 | 262 | 10 | -2.04 | 8.20E-03 | 9.67E-02 |
| AB017915 | carbohydrate (chondroitin 6) sulfotransferase 3 | Hs.158304 | 10q22.2 | CHST3 | 1032 | 16 | -2.04 | 1.52E-04 | 1.13E-02 |
| NM_006180 | neurotrophic tyrosine kinase, receptor, type 2 | Hs.439109 | 9q22.1 | NTRK2 | 155 | 16 | -2.04 | 5.58E-03 | 7.95E-02 |
| AL133568 | Homo sapiens, clone IMAGE:5268987, mRNA | Hs.511110 | 7q11-q22 | PMS2L5 | 218 | 16 | -2.04 | 8.02E-03 | 9.62E-02 |
| AK026900 | Homo sapiens cDNA: FLJ23247 fis, clone COL03425 |  |  |  | 142 | 14 | -2.03 | 2.82E-03 | 5.80E-02 |
| NM_004245 | transglutaminase 5 | Hs.129719 | 15q15.2 | TGM5 | 165 | 15 | -2.03 | 1.28E-03 | 3.87E-02 |
| AK022171 | dynactin 2 (p50) | Hs.289123 | 12q13.2-q13.3 | DCTN2 | 200 | 13 | -2.03 | 1.05E-04 | 9.06E-03 |
| AK022269 | claudin 8 | Hs.162209 | 21q22.11 | CLDN8 | 150 | 16 | -2.03 | 7.75E-03 | 9.44E-02 |
| AK024167 | keratinocytes associated protein 2 | Hs.374854 | 1q22 | KCP2 | 181 | 16 | -2.02 | 5.51E-03 | 7.88E-02 |
| AK021824 | Homo sapiens cDNA FLJ11762 fis, clone HEMBA1005670. | Hs.287453 | 17q23 | BCAS3 | 269 | 15 | -2.02 | 4.05E-03 | 6.85E-02 |
| NM_004536 | baculoviral IAP repeat-containing 1 | Hs.79019 | 5q13.1 | BIRC1 | 230 | 12 | -2.02 | 2.80E-04 | 1.59E-02 |
| NM_021784 | forkhead box A2 | Hs.155651 | 20p11 | FOXA2 | 732 | 16 | -2.02 | 1.46E-03 | 4.18E-02 |
| NM_015675 | growth arrest and DNA-damage-inducible, beta | Hs.110571 | 19p13.3 | GADD45B | 2304 | 16 | -2.02 | 7.06E-05 | 7.41E-03 |
| AK055763 | Ras association (RalGDS/AF-6) domain family 4 | Hs.512774 | 10q11.21 | RASSF4 | 332 | 15 | -2.02 | 1.88E-04 | 1.27E-02 |
| AK022346 | Homo sapiens cDNA FLJ12284 fis, clone MAMMA1001757. | Hs.45184 | 11q13.3 | ARHGEF17 | 144 | 15 | -2.02 | 4.24E-03 | 7.01E-02 |
| NM_003149 | src homology three (SH3) and cysteine rich domain | Hs.56045 | 3p22.3 | STAC | 1031 | 11 | -2.02 | 1.14E-03 | 3.65E-02 |
| NM_018329 | calcium channel, voltage-dependent, L type, alpha 1C subunit | Hs.272243 | 12p13.3 | CACNA1C | 319 | 16 | -2.01 | 4.83E-03 | 7.42E-02 |
| NM_016206 | colon carcinoma related protein | Hs.435013 | 3p12.2 | LOC51159 | 336 | 16 | -2.01 | 5.14E-03 | 7.59E-02 |
| NM_022842 | CUB domain-containing protein 1 | Hs.146170 | 3p21.32 | CDCP1 | 1200 | 14 | -2.01 | 3.07E-04 | 1.71E-02 |
| AL117519 | hypothetical protein LOC152485 | Hs.133916 | 4q31.21 | LOC152485 | 182 | 6 | -2.01 | 1.37E-03 | 4.05E-02 |
| AK001887 | protein kinase, AMP-activated, gamma 2 non-catalytic subunit | Hs.259842 | 7q35-q36 | PRKAG2 | 812 | 15 | -2.01 | 3.03E-05 | 4.40E-03 |
| NM_004126 | guanine nucleotide binding protein (G protein), gamma 11 | Hs.83381 | 7q31-q32 | GNG11 | 11481 | 15 | -2.01 | 8.31E-05 | 7.97E-03 |
| NM_001672 | agouti signaling protein, nonagouti homolog (mouse) | Hs.361642 | 20q11.2-q12 | ASIP | 139 | 13 | -2.01 | 8.84E-04 | 3.15E-02 |
| AK057064 | Homo sapiens cDNA FLJ32502 fis, clone SKNSH2000550. | Hs.510909 | 14q11.2 | na | 202 | 14 | -2.00 | 3.78E-03 | 6.63E-02 |
| AK026209 | Homo sapiens cDNA: FLJ22556 fis, clone HSI01326 | Hs.165328 | 19q13.3-q13.4 | KLK13 | 72 | 6 | -2.00 | 1.55E-03 | 4.27E-02 |
| NM_022338 | chromosome 11 open reading frame 24 | Hs.303025 | 11q13 | C11orf24 | 367 | 16 | -2.00 | 7.01E-03 | 8.99E-02 |
| NM_005737 | ADP-ribosylation factor-like 7 | Hs.111554 | 2q37.2 | ARL7 | 610 | 15 | -2.00 | 5.45E-03 | 7.87E-02 |
| M81635 | stomatin | Hs.439776 | 9q34.1 | STOM | 274 | 16 | -2.00 | 7.53E-03 | 9.34E-02 |
| NM_005543 | insulin-like 3 (Leydig cell) | Hs.37062 | 19p13.2-p12 | INSL3 | 312 | 15 | -1.99 | 6.16E-03 | 8.37E-02 |
| AL049266 | Homo sapiens mRNA; cDNA DKFZp564F093 (from clone DKFZp564F093) | Hs.350520 | 11p15.4 | TRIM6 | 71 | 10 | -1.99 | 7.60E-03 | 9.36E-02 |
| BC014604 | programmed cell death 6 | Hs.379186 | 5pter-p15.2 | PDCD6 | 259 | 16 | -1.99 | 7.65E-03 | 9.41E-02 |
| AF035306 | hypothetical protein LOC201191 | Hs.320522 | 17q21.33 | LOC201191 | 115 | 8 | -1.99 | 9.23E-04 | 3.25E-02 |
| NM_021923 | fibroblast growth factor receptor-like 1 | Hs.193326 | 4p16 | FGFRL1 | 1265 | 15 | -1.99 | 8.34E-05 | 7.94E-03 |
| AK024994 | Homo sapiens cDNA: FLJ21341 fis, clone COL02653 | Hs.306738 | 12p13.3 | ELKS | 101 | 13 | -1.99 | 6.30E-03 | 8.45E-02 |
| NM_014653 | KIAA0789 gene product |  |  |  | 274 | 13 | -1.99 | 1.05E-03 | 3.50E-02 |
| BC003517 | KIAA1218 protein | Hs.437205 | 7q22.1 | KIAA1218 | 121 | 16 | -1.99 | 6.77E-03 | 8.82E-02 |
| AK023357 | immunoglobulin superfamily, member 4 | Hs.156682 | 11q23.2 | IGSF4 | 242 | 15 | -1.98 | 4.20E-03 | 7.00E-02 |
| AL049233 | Homo sapiens mRNA; cDNA DKFZp564A023 (from clone DKFZp564A023) | Hs.432548 | 8q23-q24 | SNTB1 | 454 | 16 | -1.98 | 4.58E-03 | 7.24E-02 |
| AL049369 | Down syndrome critical region gene 1 | Hs.282326 | 21q22.1-q22.2 | DSCR1 | 408 | 14 | -1.98 | 4.29E-03 | 7.05E-02 |
| NM_024806 | hypothetical protein FLJ23554 | Hs.164705 | 11q24.1 | FLJ23554 | 127 | 16 | -1.98 | 1.41E-03 | 4.12E-02 |
| D86964 | dedicator of cyto-kinesis 2 | Hs.17211 | 5q35.1 | DOCK2 | 290 | 14 | -1.98 | 8.41E-03 | 9.74E-02 |
| AB033039 | neuron navigator 1 | Hs.6298 | 2q31.3-q32.1 | NAV1 | 249 | 16 | -1.98 | 4.90E-03 | 7.44E-02 |
| AK022170 | Homo sapiens cDNA FLJ12108 fis, clone MAMMA1000009. | Hs.432941 | 6q16.1 | RARSL | 269 | 16 | -1.98 | 4.45E-03 | 7.16E-02 |
| AK022367 | Homo sapiens cDNA FLJ12305 fis, clone MAMMA1001890. | Hs.128842 | Xq22.3 | MGC35261 | 64 | 5 | -1.98 | 7.65E-03 | 9.41E-02 |
| AK022272 | Homo sapiens cDNA FLJ12210 fis, clone MAMMA1000968. | Hs.468394 | 5q31 | BRD8 | 93 | 10 | -1.97 | 8.00E-03 | 9.61E-02 |
| BC015353 | tripartite motif-containing 43 | Hs.232026 | 2q11.2 | TRIM43 | 69 | 6 | -1.97 | 7.44E-03 | 9.28E-02 |
| AL157476 | Homo sapiens clone 23578 mRNA sequence | Hs.159388 | 19p13.3 | SAFB2 | 280 | 14 | -1.97 | 6.47E-03 | 8.60E-02 |
| AL137552 | Homo sapiens mRNA; cDNA DKFZp434E1920 (from clone DKFZp434E1920) |  |  |  | 231 | 15 | -1.97 | 8.68E-03 | 9.87E-02 |
| NM_001405 | ephrin-A2 | Hs.158306 | 19p13.3 | EFNA2 | 187 | 5 | -1.97 | 4.79E-03 | 7.37E-02 |
| AK025431 | hypothetical protein LOC253981 | Hs.283378 | 4p14 | LOC253981 | 1845 | 16 | -1.97 | 1.82E-03 | 4.66E-02 |
| AB040899 | KIAA1466 protein | Hs.147710 | 7q33 | KIAA1466 | 253 | 13 | -1.96 | 2.01E-04 | 1.30E-02 |
| AB037858 | leucine-rich repeat-containing 8 | Hs.173484 | 9q34.13 | LRRC8 | 1235 | 13 | -1.96 | 2.37E-03 | 5.30E-02 |
| NM_004178 | TAR (HIV) RNA binding protein 2 | Hs.326 | 12q12-q13 | TARBP2 | 506 | 11 | -1.96 | 3.76E-03 | 6.62E-02 |
| AK056418 | piwi-like 2 (Drosophila) | Hs.274150 | 8p21.2 | PIWIL2 | 299 | 16 | -1.96 | 7.74E-03 | 9.44E-02 |
| AK024081 | KIAA0174 gene product | Hs.287613 | 16q22.2 | KIAA0174 | 199 | 16 | -1.96 | 7.93E-03 | 9.57E-02 |
| AF247704 | NK3 transcription factor related, locus 1 (Drosophila) | Hs.55999 | 8p21 | NKX3-1 | 2117 | 16 | -1.96 | 1.78E-04 | 1.23E-02 |
| AK022190 | Homo sapiens cDNA FLJ12128 fis, clone MAMMA1000175. | Hs.287489 | 2q14 | BUB1 | 400 | 16 | -1.95 | 8.31E-03 | 9.69E-02 |
| NM_006528 | tissue factor pathway inhibitor 2 | Hs.438231 | 7q22 | TFPI2 | 412 | 16 | -1.95 | 3.94E-03 | 6.78E-02 |
| AK026687 | Homo sapiens cDNA FLJ14232 fis, clone NT2RP4000035. | Hs.101810 | 20p11.23 | C20orf23 | 444 | 16 | -1.94 | 5.71E-04 | 2.42E-02 |
| NM_015894 | stathmin-like 3 | Hs.285753 | 20q13.3 | STMN3 | 232 | 16 | -1.94 | 3.80E-03 | 6.63E-02 |
| NM_032258 | TBC1 domain family, member 3 | Hs.462906 | 17q21.1 | TBC1D3 | 445 | 12 | -1.94 | 5.26E-03 | 7.68E-02 |
| NM_018088 | hypothetical protein FLJ10408 | Hs.196086 | 12p13.31 | FLJ10408 | 259 | 16 | -1.94 | 2.46E-03 | 5.38E-02 |
| AL137712 | hypothetical protein LOC90499 | Hs.458439 | 2q11.2 | LOC90499 | 406 | 16 | -1.94 | 7.58E-04 | 2.91E-02 |
| NM_002252 | potassium voltage-gated channel, delayed-rectifier, subfamily S, member 3 | Hs.414489 | 2p24 | KCNS3 | 195 | 16 | -1.94 | 3.51E-03 | 6.42E-02 |
| NM_005204 | mitogen-activated protein kinase kinase kinase 8 | Hs.432453 | 10p12.1 | MAP3K8 | 134 | 14 | -1.94 | 2.63E-03 | 5.55E-02 |
| NM_006195 | pre-B-cell leukemia transcription factor 3 | Hs.294101 | 9q33-q34 | PBX3 | 638 | 16 | -1.94 | 1.54E-04 | 1.14E-02 |
| AL390143 | Homo sapiens mRNA; cDNA DKFZp547N074 (from clone DKFZp547N074) | Hs.284257 | 2q37 | ALPP | 148 | 14 | -1.93 | 3.19E-03 | 6.17E-02 |
| AK023793 | Homo sapiens cDNA FLJ13731 fis, clone PLACE3000142. | Hs.224012 | 19p13.1 | IL12RB1 | 111 | 12 | -1.93 | 1.86E-03 | 4.67E-02 |
| NM_033225 | CUB and Sushi multiple domains 1 | Hs.415199 | 8p23.2 | CSMD1 | 220 | 16 | -1.93 | 5.86E-03 | 8.17E-02 |
| AK000745 | LYRIC/3D3 | Hs.377155 | 8q22.1 | LYRIC | 6328 | 16 | -1.93 | 1.05E-04 | 9.02E-03 |
| AK057787 | Homo sapiens similar to LINE-1 REVERSE TRANSCRIPTASE HOMOLOG (LOC340322), mRNA | Hs.350624 | 3p14.1 | LMOD3 | 156 | 16 | -1.93 | 2.70E-03 | 5.66E-02 |
| NM_004646 | nephrosis 1, congenital, Finnish type (nephrin) | Hs.122186 | 19q13.1 | NPHS1 | 135 | 16 | -1.93 | 5.18E-03 | 7.64E-02 |
| NM_006598 | solute carrier family 12 (potassium/chloride transporters), member 7 | Hs.172613 | 5p15 | SLC12A7 | 172 | 15 | -1.93 | 1.10E-03 | 3.57E-02 |
| AK056400 | eukaryotic translation initiation factor 2B, subunit 5 epsilon, 82kDa | Hs.283551 | 3q27.3 | EIF2B5 | 200 | 6 | -1.93 | 2.59E-03 | 5.52E-02 |
| AK023111 | translocated promoter region (to activated MET oncogene) | Hs.170472 | 1q25 | TPR | 247 | 16 | -1.93 | 5.06E-03 | 7.53E-02 |
| AJ389983 | Homo sapiens partial TCR beta gene for T cell receptor beta chain variable region, germinal center 3 |  |  |  | 162 | 16 | -1.93 | 4.61E-03 | 7.27E-02 |
| NM_007115 | tumor necrosis factor, alpha-induced protein 6 | Hs.407546 | 2q24.1 | TNFAIP6 | 378 | 11 | -1.92 | 6.82E-03 | 8.84E-02 |
| AL137390 | Homo sapiens mRNA; cDNA DKFZp434P0626 (from clone DKFZp434P0626) | Hs.458893 | 15q22.32 | na | 159 | 16 | -1.92 | 5.42E-03 | 7.84E-02 |
| AK026747 | hypothetical protein LOC54103 | Hs.186649 | 7q21.11 | LOC54103 | 211 | 15 | -1.92 | 3.89E-03 | 6.73E-02 |
| AF178532 | beta-site APP-cleaving enzyme 2 | Hs.436490 | 21q22.3 | BACE2 | 646 | 15 | -1.92 | 1.78E-04 | 1.23E-02 |
| NM_003923 | forkhead box H1 | Hs.159251 | 8q24.3 | FOXH1 | 229 | 16 | -1.92 | 4.43E-03 | 7.13E-02 |
| NM_015642 | zinc finger protein 288 | Hs.436987 | 3q13.2 | ZNF288 | 949 | 16 | -1.91 | 1.83E-03 | 4.66E-02 |
| NM_017413 | apelin | Hs.303084 | Xq25-26.3 | APLN | 173 | 14 | -1.91 | 1.96E-03 | 4.80E-02 |
| AF268193 | likely ortholog of mouse IRA1 protein | Hs.438970 | 3q26.33 | IRA1 | 5485 | 16 | -1.91 | 4.78E-04 | 2.17E-02 |
| AK027156 | proprotein convertase subtilisin/kexin type 7 | Hs.443752 | 11q23-q24 | PCSK7 | 213 | 16 | -1.91 | 7.96E-03 | 9.57E-02 |
| NM_031450 | hypothetical protein p5326 | Hs.433573 | 11q13.1 | P5326 | 537 | 16 | -1.91 | 1.85E-03 | 4.67E-02 |
| AC004010 | Homo sapiens, clone IMAGE:3625286, mRNA, partial cds |  |  |  | 1880 | 16 | -1.91 | 7.00E-04 | 2.76E-02 |
| AL390157 | Homo sapiens mRNA; cDNA DKFZp434D179 (from clone DKFZp434D179) |  |  |  | 94 | 14 | -1.91 | 6.69E-03 | 8.77E-02 |
| NM_022355 | dipeptidase 2 | Hs.499331 | 16q22.1 | DPEP2 | 118 | 11 | -1.91 | 5.80E-03 | 8.11E-02 |
| BC016820 | chromosome 6 open reading frame 52 | Hs.61389 | 6p24.1 | C6orf52 | 215 | 16 | -1.91 | 3.70E-03 | 6.56E-02 |
| NM_003408 | zinc finger protein 37 homolog (mouse) | Hs.150406 | 9q32 | ZFP37 | 184 | 16 | -1.91 | 5.22E-04 | 2.30E-02 |
| NM_025114 | hypothetical protein FLJ13615 | Hs.288715 | 12q21.33 | FLJ13615 | 220 | 14 | -1.90 | 3.93E-03 | 6.77E-02 |
| NM_006106 | Yes-associated protein 1, 65kDa | Hs.170548 | 11q13 | YAP1 | 223 | 9 | -1.90 | 3.87E-03 | 6.71E-02 |
| NM_001753 | caveolin 1, caveolae protein, 22kDa | Hs.74034 | 7q31.1 | CAV1 | 4741 | 16 | -1.90 | 4.60E-04 | 2.11E-02 |
| NM_004190 | lipase, gastric | Hs.159177 | 10q23.31 | LIPF | 243 | 14 | -1.90 | 2.30E-03 | 5.20E-02 |
| NM_003489 | nuclear receptor interacting protein 1 | Hs.155017 | 21q11.2 | NRIP1 | 885 | 16 | -1.90 | 4.23E-04 | 2.04E-02 |
| NM_003679 | kynurenine 3-monooxygenase (kynurenine 3-hydroxylase) | Hs.409081 | 1q42-q44 | KMO | 280 | 16 | -1.90 | 3.57E-03 | 6.45E-02 |
| AL110283 | endo-beta-N-acetylglucosaminidase | Hs.29288 | 17q25.3 | FLJ21865 | 606 | 16 | -1.90 | 7.12E-03 | 9.07E-02 |
| AK023330 | annexin A1 | Hs.287558 | 9q12-q21.2 | ANXA1 | 379 | 15 | -1.90 | 1.43E-04 | 1.10E-02 |
| AK022157 | Homo sapiens cDNA FLJ12095 fis, clone HEMBB1002610. | Hs.301237 | 20q11.21 | KIF3B | 250 | 15 | -1.89 | 7.75E-03 | 9.44E-02 |
| AL080209 | basic transcription factor 2 | Hs.59943 | 7q34 | BBF2H7 | 1256 | 16 | -1.89 | 5.85E-03 | 8.17E-02 |
| AK057239 | Homo sapiens cDNA FLJ32677 fis, clone TESTI1000179. | Hs.524096 | 11q23.2 | LOC283140 | 189 | 16 | -1.89 | 3.21E-03 | 6.19E-02 |
| NM_015516 | hypothetical protein, estradiol-induced | Hs.8361 | 11q13.3 | E2IG4 | 475 | 16 | -1.89 | 1.33E-03 | 3.95E-02 |
| AK055365 | chromosome 14 open reading frame 146 | Hs.31522 | 14q21.1 | C14orf146 | 227 | 15 | -1.89 | 1.66E-03 | 4.43E-02 |
| NM_024956 | hypothetical protein FLJ23375 | Hs.285996 | 15q15.1 | FLJ23375 | 270 | 13 | -1.89 | 6.57E-04 | 2.64E-02 |
| BC001573 | hypothetical protein BC001573 | Hs.192586 | 5p15.31 | LOC134147 | 207 | 13 | -1.89 | 3.55E-03 | 6.43E-02 |
| NM_002438 | mannose receptor, C type 1 | Hs.75182 | 10p13 | MRC1 | 188 | 16 | -1.89 | 3.53E-03 | 6.44E-02 |
| NM_002558 | purinergic receptor P2X, ligand-gated ion channel, 1 | Hs.41735 | 17p13.3 | P2RX1 | 165 | 16 | -1.89 | 4.45E-03 | 7.16E-02 |
| AK055123 | Homo sapiens cDNA FLJ30561 fis, clone BRAWH2004580. | Hs.153618 | 8q22.3-q23.1 | RIMS2 | 89 | 11 | -1.88 | 1.41E-03 | 4.11E-02 |
| Y10183 | activated leukocyte cell adhesion molecule | Hs.10247 | 3q13.1 | ALCAM | 5490 | 16 | -1.88 | 1.97E-05 | 3.25E-03 |
| NM_005720 | actin related protein 2/3 complex, subunit 1B, 41kDa | Hs.433506 | 7q22.1 | ARPC1B | 7886 | 16 | -1.88 | 2.18E-04 | 1.36E-02 |
| AF282732 | tolloid-like 1 | Hs.129700 | 4q32-q33 | TLL1 | 74 | 11 | -1.88 | 7.71E-03 | 9.43E-02 |
| AK000939 | Homo sapiens cDNA FLJ10077 fis, clone HEMBA1001864. | Hs.494047 | 9p21 | PRO2852 | 136 | 14 | -1.88 | 2.27E-03 | 5.18E-02 |
| AL391054 | Human DNA sequence from clone RP1-228J4 on chromosome 6 Contains a pseudogene similar to UQCRFS1 (ub |  |  |  | 1144 | 16 | -1.88 | 2.60E-03 | 5.52E-02 |
| NM_002206 | integrin, alpha 7 | Hs.74369 | 12q13 | ITGA7 | 150 | 15 | -1.87 | 7.46E-03 | 9.30E-02 |
| AL133606 | hypothetical protein FLJ11142 | Hs.436628 | 3q13.2 | FLJ11142 | 164 | 13 | -1.87 | 2.88E-03 | 5.81E-02 |
| AK055460 | hypothetical protein LOC155435 | Hs.17240 | 7q36.3 | LOC155435 | 505 | 12 | -1.87 | 1.03E-03 | 3.46E-02 |
| AK022892 | zinc finger, BED domain containing 4 | Hs.13604 | 22q13.33 | ZBED4 | 135 | 16 | -1.87 | 5.39E-03 | 7.81E-02 |
| NM_014354 | chromosome 6 open reading frame 54 | Hs.225962 | 6q27 | C6orf54 | 232 | 16 | -1.87 | 8.97E-03 | 1.00E-01 |
| NM_003713 | phosphatidic acid phosphatase type 2B | Hs.432840 | 1pter-p22.1 | PPAP2B | 317 | 15 | -1.87 | 8.63E-04 | 3.12E-02 |
| NM_012083 | frequently rearranged in advanced T-cell lymphomas 2 | Hs.140720 | 10q24.1 | FRAT2 | 650 | 11 | -1.87 | 2.28E-03 | 5.19E-02 |
| NM_016352 | carboxypeptidase A4 | Hs.93764 | 7q32 | CPA4 | 366 | 16 | -1.87 | 7.26E-03 | 9.16E-02 |
| AK024471 | cytosolic nonspecific dipeptidase (EC 3.4.13.18) | Hs.149185 | 18q22.3 | CN2 | 2345 | 16 | -1.87 | 4.89E-04 | 2.20E-02 |
| NM_032318 | hypothetical protein MGC12945 | Hs.278823 | 9q22.33 | MGC12945 | 1069 | 15 | -1.86 | 1.84E-03 | 4.65E-02 |
| NM_014900 | KIAA0977 protein | Hs.443943 | 2q24.3 | KIAA0977 | 696 | 15 | -1.86 | 4.39E-03 | 7.12E-02 |
| AL117478 | activator of G-protein signaling 3 | Hs.239370 | 9q34.3 | AGS3 | 723 | 14 | -1.86 | 1.99E-04 | 1.29E-02 |
| NM_033306 | caspase 4, apoptosis-related cysteine protease | Hs.74122 | 11q22.2-q22.3 | CASP4 | 383 | 16 | -1.85 | 8.62E-03 | 9.86E-02 |
| NM_016508 | cyclin-dependent kinase-like 3 | Hs.105818 | 5q31 | CDKL3 | 266 | 11 | -1.85 | 3.22E-03 | 6.21E-02 |
| AK055963 | hypothetical protein FLJ31401 | Hs.511905 | 1q42.13 | FLJ31401 | 185 | 16 | -1.85 | 8.22E-03 | 9.68E-02 |
| NM_002229 | jun B proto-oncogene | Hs.400124 | 19p13.2 | JUNB | 329 | 16 | -1.85 | 8.77E-04 | 3.14E-02 |
| NM_001458 | filamin C, gamma (actin binding protein 280) | Hs.58414 | 7q32-q35 | FLNC | 651 | 15 | -1.85 | 1.57E-03 | 4.28E-02 |
| NM_006823 | protein kinase (cAMP-dependent, catalytic) inhibitor alpha | Hs.433700 | 8q21.11 | PKIA | 1530 | 15 | -1.85 | 4.60E-04 | 2.12E-02 |
| AF163473 | protein phosphatase 2 (formerly 2A), regulatory subunit A (PR 65), beta isoform | Hs.431156 | 11q23.2 | PPP2R1B | 216 | 16 | -1.85 | 2.18E-03 | 5.08E-02 |
| NM_016617 | hypothetical protein BM-002 | Hs.367646 | 13q13.3 | BM-002 | 908 | 13 | -1.84 | 4.38E-04 | 2.07E-02 |
| NM_006238 | peroxisome proliferative activated receptor, delta | Hs.106415 | 6p21.2-p21.1 | PPARD | 271 | 13 | -1.84 | 2.42E-03 | 5.33E-02 |
| NM_002149 | hippocalcin-like 1 | Hs.3618 | 2p25.1 | HPCAL1 | 2452 | 14 | -1.84 | 2.88E-04 | 1.63E-02 |
| AK025038 | Homo sapiens cDNA: FLJ21385 fis, clone COL03356 | Hs.306750 | 3q22.1 | PIK3R4 | 134 | 16 | -1.84 | 5.62E-03 | 7.98E-02 |
| NM_015920 | ribosomal protein S27-like | Hs.108957 | 15q22.1 | RPS27L | 3213 | 16 | -1.84 | 8.73E-05 | 8.07E-03 |
| NM_002066 | GPI anchored molecule like protein | Hs.86161 | 8q24.3 | GML | 156 | 15 | -1.84 | 7.42E-03 | 9.28E-02 |
| BC010549 | NEDD8-conjugating enzyme | Hs.157804 | 2q37.3 | NCE2 | 640 | 15 | -1.84 | 3.41E-05 | 4.73E-03 |
| NM_003545 | histone 1, H4e | Hs.240135 | 6p21.3 | HIST1H4E | 311 | 15 | -1.84 | 1.70E-03 | 4.49E-02 |
| NM_030956 | toll-like receptor 10 | Hs.120551 | 4p14 | TLR10 | 156 | 15 | -1.84 | 8.62E-03 | 9.87E-02 |
| AK057119 | hypothetical protein LOC63929 | Hs.20797 | 22q13.31-q13.33 | LOC63929 | 132 | 11 | -1.84 | 4.86E-03 | 7.42E-02 |
| NM_006229 | pancreatic lipase-related protein 1 | Hs.73923 | 10q26.12 | PNLIPRP1 | 192 | 16 | -1.84 | 3.46E-03 | 6.38E-02 |
| NM_030916 | Ig superfamily receptor LNIR | Hs.61460 | 1q22-q23.2 | LNIR | 111 | 16 | -1.84 | 5.37E-03 | 7.79E-02 |
| NM_001848 | collagen, type VI, alpha 1 | Hs.415997 | 21q22.3 | COL6A1 | 167 | 15 | -1.84 | 8.64E-03 | 9.87E-02 |
| AK025140 | hypothetical protein FLJ32421 | Hs.6236 | 1q42.12 | FLJ32421 | 3432 | 11 | -1.83 | 3.47E-03 | 6.38E-02 |
| BE379813 | hypothetical protein DKFZp564C0469 | Hs.285055 | 5q15 | DKFZp564C0469 | 464 | 16 | -1.83 | 7.71E-03 | 9.42E-02 |
| NM_002741 | protein kinase C-like 1 | Hs.2499 | 19p13.1-p12 | PRKCL1 | 631 | 14 | -1.83 | 8.88E-03 | 9.97E-02 |
| NM_015535 | DKFZP564A2416 protein | Hs.230767 | 2q33.2 | DKFZP564A2416 | 4203 | 16 | -1.83 | 2.72E-05 | 4.11E-03 |
| NM_002357 | MAX dimerization protein 1 | Hs.379930 | 2p13-p12 | MAD | 1699 | 16 | -1.83 | 2.05E-04 | 1.30E-02 |
| AK025736 | 3-hydroxy-3-methylglutaryl-Coenzyme A synthase 1 (soluble) | Hs.397729 | 5p14-p13 | HMGCS1 | 1178 | 15 | -1.83 | 6.27E-03 | 8.41E-02 |
| U11058 | potassium large conductance calcium-activated channel, subfamily M, alpha member 1 | Hs.354740 | 10q22-q23 | KCNMA1 | 343 | 16 | -1.83 | 3.01E-03 | 5.95E-02 |
| NM_005715 | uronyl-2-sulfotransferase | Hs.131514 | 6q24.3 | UST | 216 | 13 | -1.83 | 8.97E-03 | 9.99E-02 |
| NM_014917 | netrin G1f | Hs.111224 | 1p13.3 | KIAA0976 | 172 | 10 | -1.82 | 8.71E-03 | 9.88E-02 |
| AB040927 | SH3 domain containing ring finger | Hs.301804 | 4q32.3 | SH3RF | 879 | 15 | -1.82 | 1.76E-04 | 1.23E-02 |
| AB058779 | euchromatic histone methyltransferase 1 | Hs.416692 | 9q34.3 | Eu-HMTase1 | 106 | 15 | -1.82 | 6.78E-03 | 8.82E-02 |
| NM_002414 | CD99 antigen | Hs.283477 | Xp22.32 | CD99 | 2453 | 16 | -1.82 | 7.43E-03 | 9.28E-02 |
| AL157417 | Homo sapiens mRNA; cDNA DKFZp761E10121 (from clone DKFZp761E10121); partial cds |  |  |  | 73 | 8 | -1.82 | 5.50E-03 | 7.88E-02 |
| NM_015379 | brain protein I3 | Hs.523441 | 7q22.1 | BRI3 | 2568 | 16 | -1.82 | 5.66E-04 | 2.43E-02 |
| NM_003704 | chromosome 4 open reading frame 8 | Hs.325987 | 4p16.3 | C4orf8 | 352 | 14 | -1.82 | 5.24E-03 | 7.68E-02 |
| NM_002665 | plasminogen-like | Hs.406278 | 2p11-q11 | PLGL | 146 | 14 | -1.82 | 2.03E-03 | 4.91E-02 |
| AL049218 | hypothetical protein FLJ11712 | Hs.306291 | 13q14.13 | FLJ11712 | 165 | 15 | -1.82 | 6.98E-03 | 8.98E-02 |
| NM_001189 | bagpipe homeobox homolog 1 (Drosophila) | Hs.105941 | 4p16.1 | BAPX1 | 375 | 16 | -1.82 | 7.47E-03 | 9.30E-02 |
| NM_002280 | keratin, hair, acidic, 5 | Hs.73082 | 17q12-q21 | KRTHA5 | 107 | 16 | -1.82 | 8.64E-03 | 9.88E-02 |
| NM_006383 | DNA-dependent protein kinase catalytic subunit-interacting protein 2 | Hs.129867 | 15q24 | KIP2 | 121 | 11 | -1.82 | 8.79E-03 | 9.94E-02 |
| NM_024509 | hypothetical protein MGC2656 | Hs.143792 | 19q13.13 | MGC2656 | 193 | 13 | -1.81 | 3.31E-03 | 6.23E-02 |
| AL136785 | HSPC065 protein | Hs.11614 | 16q13 | HSPC065 | 2524 | 15 | -1.81 | 3.67E-04 | 1.84E-02 |
| NM_032564 | diacylglycerol O-acyltransferase homolog 2 (mouse) | Hs.334305 | 11q13.3 | DGAT2 | 232 | 15 | -1.81 | 3.43E-03 | 6.35E-02 |
| AY007149 | centrosome-associated protein 350 | Hs.413045 | 1p36.13-q41 | CAP350 | 492 | 16 | -1.81 | 1.21E-03 | 3.78E-02 |
| AK056708 | rhomboid family 1 (Drosophila) | Hs.57988 | 16p13.3 | RHBDF1 | 351 | 12 | -1.81 | 1.22E-03 | 3.79E-02 |
| NM_001792 | cadherin 2, type 1, N-cadherin (neuronal) | Hs.334131 | 18q11.2 | CDH2 | 5851 | 15 | -1.81 | 2.26E-03 | 5.20E-02 |
| NM_006193 | paired box gene 4 | Hs.129706 | 7q32 | PAX4 | 131 | 15 | -1.81 | 8.82E-03 | 9.96E-02 |
| AL137501 | Homo sapiens mRNA; cDNA DKFZp761G241 (from clone DKFZp761G241) | Hs.306470 | 22q11.21 | DGCR14 | 144 | 16 | -1.81 | 4.57E-03 | 7.24E-02 |
| AB014514 | KIAA0614 protein |  |  |  | 909 | 13 | -1.81 | 3.78E-03 | 6.62E-02 |
| AK000144 | Homo sapiens cDNA FLJ20137 fis, clone COL07137 | Hs.274449 | 17q23.2 | FLJ11029 | 100 | 13 | -1.81 | 3.37E-03 | 6.29E-02 |
| NM_021179 | hypothetical protein LOC57821 | Hs.130746 | 1q24 | LOC57821 | 75 | 10 | -1.80 | 8.35E-03 | 9.72E-02 |
| NM_006808 | protein translocation complex beta | Hs.191887 | 9q22.32-q31.3 | SEC61B | 7940 | 16 | -1.80 | 4.88E-03 | 7.43E-02 |
| NM_005904 | MAD, mothers against decapentaplegic homolog 7 (Drosophila) | Hs.370849 | 18q21.1 | MADH7 | 257 | 14 | -1.80 | 2.91E-03 | 5.84E-02 |
| NM_001850 | collagen, type VIII, alpha 1 | Hs.114599 | 3q12.3 | COL8A1 | 534 | 13 | -1.80 | 8.36E-03 | 9.71E-02 |
| AK024375 | abhydrolase domain containing 5 | Hs.19385 | 3p21 | ABHD5 | 200 | 15 | -1.80 | 7.69E-03 | 9.43E-02 |
| AL390128 | KIAA1530 protein | Hs.380475 | 4p16.3 | KIAA1530 | 312 | 15 | -1.80 | 8.58E-04 | 3.11E-02 |
| AK057427 | cytochrome c oxidase subunit VIb, testes-specific | Hs.329540 | 19q13.42 | COXVIB2 | 250 | 14 | -1.80 | 2.05E-04 | 1.31E-02 |
| AF104032 | solute carrier family 7 (cationic amino acid transporter, y+ system), member 5 | Hs.184601 | 16q24.3 | SLC7A5 | 8711 | 16 | -1.80 | 1.39E-04 | 1.08E-02 |
| NM_033296 | T-cell activation protein | Hs.406590 | 4p16.1 | PGR1 | 2339 | 15 | -1.80 | 2.71E-03 | 5.65E-02 |
| NM_032623 | ovary-specific acidic protein | Hs.154140 | 4q31.1 | OSAP | 148 | 13 | -1.80 | 2.77E-03 | 5.73E-02 |
| AB051532 | sema domain, immunoglobulin domain (Ig), transmembrane domain (TM) and short cytoplasmic domain, (semaphorin) 4B | Hs.416077 | 15q25 | SEMA4B | 635 | 16 | -1.79 | 4.96E-04 | 2.22E-02 |
| AB046844 | G protein-coupled receptor 107 | Hs.442329 | 9q34.2 | GPR107 | 389 | 14 | -1.79 | 7.71E-03 | 9.43E-02 |
| AK023833 | H2A histone family, member Y | Hs.75258 | 5q31.3-q32 | H2AFY | 378 | 15 | -1.79 | 7.27E-03 | 9.16E-02 |
| AK056213 | Homo sapiens cDNA FLJ31651 fis, clone NT2RI2004136. | Hs.150179 | 17q11 | SARM1 | 894 | 16 | -1.79 | 3.97E-03 | 6.79E-02 |
| NM_000868 | 5-hydroxytryptamine (serotonin) receptor 2C | Hs.46362 | Xq24 | HTR2C | 252 | 16 | -1.79 | 6.06E-03 | 8.32E-02 |
| NM_018327 | chromosome 20 open reading frame 38 | Hs.272242 | 20p12.1 | C20orf38 | 105 | 16 | -1.79 | 4.26E-03 | 7.02E-02 |
| NM_005430 | wingless-type MMTV integration site family, member 1 | Hs.248164 | 12q13 | WNT1 | 128 | 16 | -1.79 | 2.77E-03 | 5.73E-02 |
| AL136883 | putative homeodomain transcription factor 2 | Hs.128653 | 7q11.23-q21 | PHTF2 | 598 | 11 | -1.78 | 8.89E-03 | 9.97E-02 |
| AK025364 | KIAA1421 protein | Hs.117268 | 15q21.3 | KIAA1421 | 112 | 15 | -1.78 | 4.85E-03 | 7.43E-02 |
| AL050124 | Homo sapiens mRNA; cDNA DKFZp586E151 (from clone DKFZp586E151) |  |  |  | 225 | 15 | -1.78 | 4.70E-03 | 7.34E-02 |
| AL049545 | Human DNA sequence from clone 263J7 on chromosome 6q14.3-15. Contains an RPL7 (60S Ribosomal Protein |  |  |  | 319 | 14 | -1.78 | 6.76E-03 | 8.82E-02 |
| AB037770 | KIAA1349 protein | Hs.127656 | 17p11.2 | KIAA1349 | 239 | 15 | -1.78 | 6.61E-03 | 8.71E-02 |
| AF183185 | otoferlin | Hs.91608 | 2p23.1 | OTOF | 1294 | 13 | -1.78 | 9.83E-05 | 8.77E-03 |
| NM_004295 | TNF receptor-associated factor 4 | Hs.8375 | 17q11-q12 | TRAF4 | 1368 | 16 | -1.77 | 7.01E-04 | 2.76E-02 |
| NM_021642 | Fc fragment of IgG, low affinity IIa, receptor for (CD32) | Hs.352642 | 1q23 | FCGR2A | 1237 | 14 | -1.77 | 1.39E-03 | 4.09E-02 |
| NM_018702 | adenosine deaminase, RNA-specific, B2 (RED2 homolog rat) | Hs.192731 | 10p15.3 | ADARB2 | 88 | 7 | -1.77 | 2.53E-03 | 5.44E-02 |
| AK055508 | Rad50-interacting protein 1 | Hs.302460 | 7q22.2 | FLJ11785 | 2009 | 16 | -1.77 | 7.56E-04 | 2.91E-02 |
| NM_058165 | diacylglycerol O-acyltransferase 2 like 1 | Hs.344090 | 2q36.2 | DGAT2L1 | 217 | 13 | -1.76 | 5.46E-03 | 7.87E-02 |
| NM_017880 | hypothetical protein FLJ20558 | Hs.413123 | 2p13.3 | FLJ20558 | 182 | 10 | -1.76 | 4.71E-03 | 7.35E-02 |
| NM_032262 | hypothetical protein DKFZp434N035 | Hs.333118 | 22q11.21 | DKFZp434N035 | 247 | 13 | -1.76 | 7.98E-04 | 2.99E-02 |
| AK025773 | lectin, mannose-binding, 1 | Hs.436593 | 18q21.3-q22 | LMAN1 | 1383 | 12 | -1.76 | 3.95E-03 | 6.79E-02 |
| NM_024998 | hypothetical protein FLJ12704 | Hs.287541 | 1q32.2 | FLJ12704 | 137 | 10 | -1.76 | 4.67E-03 | 7.32E-02 |
| AF218021 | Homo sapiens clone PP552 unknown mRNA | Hs.464311 | 17q25.3 | LOC146713 | 548 | 16 | -1.75 | 7.75E-03 | 9.44E-02 |
| AK026808 | DKFZP564G092 protein | Hs.96867 | 10q22.1 | DKFZP564G092 | 190 | 13 | -1.75 | 3.02E-03 | 5.97E-02 |
| NM_014135 | hypothetical protein MGC4308 | Hs.8345 | 3q12.3 | MGC4308 | 777 | 16 | -1.75 | 1.08E-03 | 3.53E-02 |
| AF119913 | Homo sapiens PRO3077 mRNA, complete cds |  |  |  | 377 | 14 | -1.75 | 8.30E-03 | 9.70E-02 |
| NM_003772 | jerky homolog-like (mouse) | Hs.105940 | 11q21 | JRKL | 332 | 15 | -1.75 | 8.26E-03 | 9.67E-02 |
| AK000208 | hypothetical protein MGC50844 | Hs.27267 | 7q32.3 | MGC50844 | 619 | 16 | -1.75 | 8.74E-04 | 3.14E-02 |
| AK057198 | Homo sapiens cDNA FLJ32636 fis, clone SYNOV2000193. | Hs.196065 | 6q11.2 | SLM1 | 242 | 16 | -1.75 | 6.97E-03 | 8.98E-02 |
| AL137727 | chromosome 14 open reading frame 9 | Hs.7001 | 14q11.2 | C14orf9 | 438 | 16 | -1.74 | 5.95E-03 | 8.24E-02 |
| NM_004339 | pituitary tumor-transforming 1 interacting protein | Hs.369026 | 21q22.3 | PTTG1IP | 2940 | 16 | -1.74 | 6.21E-03 | 8.42E-02 |
| NM_006826 | tyrosine 3-monooxygenase/tryptophan 5-monooxygenase activation protein, theta polypeptide | Hs.74405 | 2p25.2-p25.1 | YWHAQ | 12631 | 16 | -1.74 | 8.36E-04 | 3.08E-02 |
| NM_004676 | PTPN13-like, Y-linked | Hs.158343 | Yq11.223 | PRY | 198 | 13 | -1.74 | 4.23E-03 | 7.01E-02 |
| AF216077 | collagen, type XXVII, alpha 1 | Hs.59892 | 9q33.1 | COL27A1 | 591 | 15 | -1.74 | 1.73E-03 | 4.55E-02 |
| NM_002526 | 5'-nucleotidase, ecto (CD73) | Hs.153952 | 6q14-q21 | NT5E | 343 | 13 | -1.74 | 7.76E-03 | 9.44E-02 |
| NM_024426 | Wilms tumor 1 | Hs.1145 | 11p13 | WT1 | 217 | 16 | -1.74 | 9.18E-04 | 3.25E-02 |
| BC008651 | Homo sapiens LOC342935 (LOC342935), mRNA | Hs.23133 | 16q13-q21 | KIFC3 | 856 | 16 | -1.74 | 7.52E-03 | 9.34E-02 |
| AK022301 | Homo sapiens cDNA FLJ12239 fis, clone MAMMA1001268. | Hs.486167 | 6q21 | na | 120 | 8 | -1.74 | 8.84E-03 | 9.96E-02 |
| AK024536 | Ca2+-dependent activator protein for secretion | Hs.343969 | 3p21.1 | CADPS | 5809 | 16 | -1.73 | 8.87E-03 | 9.98E-02 |
| NM_032882 | hypothetical protein MGC15827 | Hs.11849 | Xq28 | MGC15827 | 589 | 16 | -1.73 | 4.20E-03 | 6.99E-02 |
| NM_002957 | retinoid X receptor, alpha | Hs.20084 | 9q34.3 | RXRA | 1103 | 16 | -1.73 | 1.31E-03 | 3.91E-02 |
| NM_016265 | zinc finger protein 325 | Hs.102397 | 7p22.2 | ZNF325 | 535 | 15 | -1.73 | 1.39E-03 | 4.08E-02 |
| NM_005746 | pre-B-cell colony-enhancing factor | Hs.293464 | 7q22.2 | PBEF | 1086 | 15 | -1.73 | 4.63E-03 | 7.29E-02 |
| AK055538 | chromosome 6 open reading frame 199 | Hs.124370 | 6q21 | C6orf199 | 142 | 16 | -1.73 | 7.05E-03 | 9.02E-02 |
| NM_002167 | inhibitor of DNA binding 3, dominant negative helix-loop-helix protein | Hs.76884 | 1p36.13-p36.12 | ID3 | 7787 | 16 | -1.73 | 1.91E-03 | 4.74E-02 |
| AK057207 | uveal autoantigen with coiled-coil domains and ankyrin repeats | Hs.49753 | 15q22-q24 | UACA | 968 | 16 | -1.72 | 1.74E-03 | 4.56E-02 |
| NM_024915 | hypothetical protein FLJ13782 | Hs.389571 | 8q22.3 | FLJ13782 | 289 | 16 | -1.72 | 5.71E-04 | 2.42E-02 |
| NM_000362 | tissue inhibitor of metalloproteinase 3 (Sorsby fundus dystrophy, pseudoinflammatory) | Hs.245188 | 22q12.1-q13.2 | TIMP3 | 1900 | 16 | -1.72 | 2.55E-03 | 5.48E-02 |
| NM_002309 | leukemia inhibitory factor (cholinergic differentiation factor) | Hs.2250 | 22q12.2 | LIF | 915 | 15 | -1.72 | 1.47E-03 | 4.19E-02 |
| AK056188 | NS1-associated protein 1 | Hs.436376 | 6q14-q15 | NSAP1 | 700 | 16 | -1.72 | 4.16E-03 | 6.97E-02 |
| NM_018083 | zinc finger protein 358 | Hs.133475 | 10q23.3-q24 | ZNF358 | 535 | 14 | -1.72 | 3.97E-04 | 1.93E-02 |
| NM_032551 | G protein-coupled receptor 54 | Hs.208229 | 19p13.3 | GPR54 | 141 | 12 | -1.72 | 5.13E-03 | 7.58E-02 |
| NM_021925 | hypothetical protein FLJ21820 | Hs.63300 | 2p24.2 | FLJ21820 | 346 | 16 | -1.72 | 2.76E-03 | 5.72E-02 |
| NM_007098 | clathrin, heavy polypeptide-like 1 | Hs.184916 | 22q11.2 | CLTCL1 | 241 | 10 | -1.72 | 7.40E-03 | 9.26E-02 |
| AK025582 | guanine nucleotide binding protein (G protein) alpha 12 | Hs.182874 | 7p22-p21 | GNA12 | 1012 | 13 | -1.71 | 8.15E-03 | 9.65E-02 |
| AK057162 | WD repeat domain 10 | Hs.117920 | 3q21 | WDR10 | 224 | 16 | -1.71 | 4.34E-03 | 7.07E-02 |
| AK026667 | Homo sapiens cDNA: FLJ23014 fis, clone LNG00806 | Hs.512477 | 14 | bA327L3.4 | 147 | 14 | -1.71 | 3.29E-03 | 6.23E-02 |
| NM_002337 | low density lipoprotein receptor-related protein associated protein 1 | Hs.75140 | 4p16.3 | LRPAP1 | 2736 | 15 | -1.71 | 2.04E-03 | 4.89E-02 |
| BC010012 | WW domain binding protein 1 | Hs.7709 | 2p12 | WBP1 | 988 | 9 | -1.70 | 2.68E-03 | 5.63E-02 |
| NM_001872 | carboxypeptidase B2 (plasma, carboxypeptidase U) | Hs.221926 | 13q14.11 | CPB2 | 578 | 13 | -1.70 | 8.07E-03 | 9.63E-02 |
| NM_024341 | hypothetical protein MGC4054 | Hs.250821 | 19p13.3 | MGC4054 | 235 | 16 | -1.70 | 5.10E-03 | 7.57E-02 |
| AK057798 | Homo sapiens cDNA FLJ25069 fis, clone CBL05145 |  |  |  | 314 | 16 | -1.70 | 8.82E-03 | 9.96E-02 |
| AF131762 | carbohydrate (chondroitin 4) sulfotransferase 11 | Hs.433059 | 12q | CHST11 | 302 | 15 | -1.70 | 8.89E-03 | 9.98E-02 |
| AL162070 | coronin, actin binding protein, 1C | Hs.17377 | 12q24.1 | CORO1C | 1646 | 15 | -1.70 | 2.62E-03 | 5.55E-02 |
| NM_015907 | leucine aminopeptidase 3 | Hs.182579 | 4p15.33 | LAP3 | 2721 | 16 | -1.70 | 4.46E-03 | 7.16E-02 |
| NM_014489 | FGF receptor activating protein 1 | Hs.133968 | 11p15.5 | FRAG1 | 210 | 13 | -1.69 | 5.04E-03 | 7.52E-02 |
| NM_002938 | ring finger protein 4 | Hs.66394 | 4p16.3 | RNF4 | 396 | 9 | -1.69 | 2.47E-03 | 5.39E-02 |
| AK054637 | hypothetical protein LOC149773 | Hs.473192 | 20q13.32 | LOC149773 | 245 | 16 | -1.69 | 8.22E-03 | 9.68E-02 |
| NM_022736 | hypothetical protein FLJ14153 | Hs.7503 | 3q25.32 | FLJ14153 | 964 | 12 | -1.69 | 4.74E-03 | 7.34E-02 |
| NM_004300 | acid phosphatase 1, soluble | Hs.130873 | 2p25 | ACP1 | 604 | 15 | -1.69 | 5.32E-03 | 7.74E-02 |
| AF339789 | Homo sapiens clone IMAGE:212461, mRNA sequence | Hs.369317 | 2q37.3 | na | 379 | 11 | -1.69 | 7.77E-03 | 9.44E-02 |
| AB051502 | Lunapark | Hs.209561 | 2q31 | LNP | 184 | 14 | -1.69 | 2.03E-03 | 4.91E-02 |
| AB014599 | coiled-coil protein BICD2 | Hs.436939 | 9q22.32 | BICD2 | 308 | 16 | -1.68 | 6.27E-04 | 2.56E-02 |
| AK025746 | likely ortholog of mouse IRA1 protein | Hs.438970 | 3q26.33 | IRA1 | 1690 | 16 | -1.68 | 1.18E-03 | 3.73E-02 |
| BC008882 | hypothetical protein FLJ13909 | Hs.288672 | 16p13.3 | FLJ13909 | 131 | 12 | -1.68 | 1.92E-03 | 4.74E-02 |
| AF070643 | intraflagellar transport protein IFT20 | Hs.4187 | 17q11.2 | LOC90410 | 1298 | 16 | -1.68 | 7.87E-04 | 2.98E-02 |
| NM_017957 | epsin 3 | Hs.165904 | 17q21.33 | EPN3 | 94 | 11 | -1.68 | 4.25E-03 | 7.02E-02 |
| AF361746 | similar to endothelial cell-selective adhesion molecule | Hs.173840 | 11q24.2 | ESAM | 149 | 7 | -1.68 | 3.37E-03 | 6.29E-02 |
| AK026195 | Homo sapiens cDNA: FLJ22542 fis, clone HSI00196 | Hs.146747 | 11q12 | P2RX3 | 319 | 16 | -1.68 | 4.75E-03 | 7.36E-02 |
| Y16961 | tumor protein p73-like | Hs.137569 | 3q27-q29 | TP73L | 116 | 12 | -1.68 | 8.46E-03 | 9.75E-02 |
| NM_004669 | chloride intracellular channel 3 | Hs.64746 | 9q34.3 | CLIC3 | 329 | 15 | -1.67 | 7.93E-03 | 9.57E-02 |
| AK057033 | CGI-147 protein | Hs.12677 | 17q23.2 | CGI-147 | 727 | 11 | -1.67 | 3.22E-03 | 6.22E-02 |
| NM_013316 | CCR4-NOT transcription complex, subunit 4 | Hs.20423 | 7q22-qter | CNOT4 | 615 | 15 | -1.67 | 6.25E-03 | 8.42E-02 |
| NM_004935 | cyclin-dependent kinase 5 | Hs.166071 | 7q36 | CDK5 | 765 | 14 | -1.67 | 4.51E-04 | 2.09E-02 |
| BC004968 | Homo sapiens, clone IMAGE:3543963, mRNA | Hs.121180 | 6p21.3 | RNF39 | 394 | 12 | -1.67 | 6.45E-03 | 8.58E-02 |
| NM_031946 | centaurin, gamma 3 | Hs.249728 | 7q36.1 | CENTG3 | 1467 | 15 | -1.66 | 3.69E-03 | 6.56E-02 |
| AF402776 | Homo sapiens BIC noncoding mRNA, complete sequence | Hs.517226 | 1q21-q22 | FY | 435 | 16 | -1.66 | 1.78E-04 | 1.24E-02 |
| NM_018641 | carbohydrate (chondroitin 4) sulfotransferase 12 | Hs.25204 | 7p22 | CHST12 | 947 | 12 | -1.66 | 7.35E-03 | 9.22E-02 |
| AK027380 | calumenin | Hs.7753 | 7q32 | CALU | 594 | 13 | -1.66 | 7.98E-04 | 2.99E-02 |
| NM_021807 | secretory protein SEC8 | Hs.446140 | 7q31 | SEC8 | 2003 | 15 | -1.66 | 7.65E-04 | 2.93E-02 |
| AK002019 | hypothetical protein BC009518 | Hs.387385 | 17q24.2 | LOC90799 | 556 | 16 | -1.66 | 5.24E-03 | 7.67E-02 |
| AL137349 | hypothetical protein DKFZP434A0225 | Hs.83293 | 7q21.3 | DKFZP434A0225 | 1487 | 16 | -1.65 | 3.29E-03 | 6.23E-02 |
| NM_015149 | RalGDS-like gene | Hs.79219 | 1q25.2 | RGL | 320 | 15 | -1.65 | 2.51E-03 | 5.43E-02 |
| AK023110 | HSPC182 protein | Hs.30026 | 1p36.33 | HSPC182 | 524 | 16 | -1.65 | 5.28E-04 | 2.31E-02 |
| NM_031458 | B aggressive lymphoma gene | Hs.131315 | 3q13-q21 | BAL | 276 | 16 | -1.65 | 5.23E-03 | 7.67E-02 |
| AJ251595 | CD44 antigen (homing function and Indian blood group system) | Hs.306278 | 11p13 | CD44 | 1950 | 16 | -1.65 | 3.33E-03 | 6.24E-02 |
| NM_032799 | zinc finger, DHHC domain containing 12 | Hs.133122 | 9q34.13 | ZDHHC12 | 1214 | 15 | -1.64 | 1.37E-03 | 4.06E-02 |
| AK022838 | GRIP coiled-coil protein GCC185 | Hs.278671 | 2q12.3 | GCC185 | 464 | 16 | -1.64 | 2.37E-03 | 5.30E-02 |
| AL133050 | KIAA1713 protein | Hs.307003 | 18q11 | KIAA1713 | 144 | 12 | -1.64 | 5.30E-03 | 7.73E-02 |
| NM_005645 | TAF13 RNA polymerase II, TATA box binding protein (TBP)-associated factor, 18kDa | Hs.502508 | 1p13.1 | TAF13 | 1501 | 14 | -1.64 | 5.40E-04 | 2.34E-02 |
| NM_015369 | TP53TG3 protein | Hs.513543 | 16p13 | TP53TG3 | 1247 | 13 | -1.64 | 6.63E-03 | 8.71E-02 |
| NM_004059 | cysteine conjugate-beta lyase; cytoplasmic (glutamine transaminase K, kyneurenine aminotransferase) | Hs.382311 | 9q34.13 | CCBL1 | 373 | 16 | -1.64 | 6.81E-03 | 8.84E-02 |
| AK025557 | trans-golgi network protein 2 | Hs.14894 | 2p11.2 | TGOLN2 | 1794 | 16 | -1.64 | 8.09E-03 | 9.62E-02 |
| NM_032503 | G protein-coupled receptor 145 | Hs.333173 | 6q16 | GPR145 | 153 | 12 | -1.64 | 7.52E-03 | 9.33E-02 |
| AL133576 | neighbor of A-kinase anchoring protein 95 | Hs.96200 | 19p13.13-p13.12 | NAKAP95 | 158 | 13 | -1.64 | 6.65E-03 | 8.73E-02 |
| NM_007100 | ATP synthase, H+ transporting, mitochondrial F0 complex, subunit e | Hs.85539 | 4p16.3 | ATP5I | 3927 | 16 | -1.64 | 9.35E-04 | 3.25E-02 |
| NM_003914 | cyclin A1 | Hs.417050 | 13q12.3-q13 | CCNA1 | 431 | 16 | -1.64 | 9.65E-04 | 3.31E-02 |
| AB058744 | KIAA1841 protein | Hs.223206 | 2q14 | KIAA1841 | 248 | 11 | -1.64 | 5.60E-03 | 7.98E-02 |
| NM_032842 | hypothetical protein FLJ14803 | Hs.267245 | 7q32.3 | FLJ14803 | 739 | 15 | -1.63 | 2.18E-03 | 5.08E-02 |
| AK057103 | zinc finger protein 318 | Hs.147868 | 6pter-p12.1 | ZNF318 | 144 | 9 | -1.63 | 8.42E-03 | 9.74E-02 |
| NM_012396 | pleckstrin homology-like domain, family A, member 3 | Hs.268557 | 1q31 | PHLDA3 | 948 | 15 | -1.63 | 8.55E-04 | 3.10E-02 |
| AB051510 | deleted in liver cancer 1 | Hs.8700 | 8p22 | DLC1 | 488 | 15 | -1.63 | 2.48E-03 | 5.40E-02 |
| Y16645 | chemokine (C-C motif) ligand 8 | Hs.271387 | 17q11.2 | CCL8 | 146 | 16 | -1.63 | 7.70E-03 | 9.43E-02 |
| AK055660 | myotrophin | Hs.21321 | 7q33 | MTPN | 5221 | 15 | -1.63 | 5.86E-03 | 8.17E-02 |
| NM_032831 | chromosome 7 open reading frame 19 | Hs.289053 | 7q22.1 | C7orf19 | 383 | 16 | -1.63 | 1.67E-03 | 4.44E-02 |
| NM_002829 | protein tyrosine phosphatase, non-receptor type 3 | Hs.405666 | 9q31 | PTPN3 | 956 | 16 | -1.63 | 4.80E-03 | 7.38E-02 |
| BC015135 | Homo sapiens, clone IMAGE:3935677, mRNA | Hs.348631 | 2q11.2 | DKFZp434N062 | 646 | 15 | -1.63 | 5.71E-03 | 8.04E-02 |
| AK054606 | zinc finger protein 79 (pT7) | Hs.512719 | 9q34 | ZNF79 | 138 | 12 | -1.63 | 4.99E-03 | 7.48E-02 |
| NM_014622 | loss of heterozygosity, 11, chromosomal region 2, gene A | Hs.152944 | 11q23 | LOH11CR2A | 101 | 12 | -1.63 | 3.04E-03 | 6.00E-02 |
| NM_004710 | synaptogyrin 2 | Hs.433753 | 17q25.3 | SYNGR2 | 1372 | 16 | -1.62 | 4.46E-04 | 2.08E-02 |
| AK026829 | laminin, alpha 2 (merosin, congenital muscular dystrophy) | Hs.445120 | 6q22-q23 | LAMA2 | 210 | 14 | -1.62 | 4.34E-03 | 7.07E-02 |
| D26488 | KIAA0007 protein | Hs.90315 | 2p23.3 | KIAA0007 | 2070 | 16 | -1.62 | 9.39E-04 | 3.24E-02 |
| NM_032158 | Williams Beuren syndrome chromosome region 20C | Hs.436034 | 7q11.23 | WBSCR20C | 5322 | 16 | -1.62 | 4.03E-03 | 6.85E-02 |
| NM_018357 | acheron | Hs.416755 | 15q22.32 | FLJ11196 | 551 | 16 | -1.61 | 1.21E-03 | 3.78E-02 |
| BC009033 | hypothetical protein LOC253982 | Hs.323815 | 16p12.1 | LOC253982 | 739 | 16 | -1.61 | 6.54E-04 | 2.65E-02 |
| AB002308 | KIAA0310 gene product | Hs.396443 | 9q34.3 | KIAA0310 | 1460 | 14 | -1.60 | 1.48E-03 | 4.20E-02 |
| AL157424 | synaptojanin 2 | Hs.434494 | 6q25.3 | SYNJ2 | 587 | 16 | -1.60 | 2.28E-03 | 5.19E-02 |
| BC016962 | Homo sapiens mRNA; cDNA DKFZp586B211 (from clone DKFZp586B211) | Hs.16193 | 19p13.12 | MDS032 | 377 | 12 | -1.60 | 4.72E-03 | 7.34E-02 |
| AB007931 | retinoblastoma-associated factor 600 | Hs.287616 | 1p36.13 | RBAF600 | 1332 | 16 | -1.60 | 6.32E-03 | 8.45E-02 |
| BC011406 | hypothetical protein MGC3036 | Hs.284135 | 7q31-q35 | MGC3036 | 364 | 16 | -1.60 | 4.93E-03 | 7.47E-02 |
| NM_002298 | lymphocyte cytosolic protein 1 (L-plastin) | Hs.381099 | 13q14.3 | LCP1 | 201 | 13 | -1.59 | 6.48E-03 | 8.60E-02 |
| NM_005025 | serine (or cysteine) proteinase inhibitor, clade I (neuroserpin), member 1 | Hs.78589 | 3q26.2 | SERPINI1 | 281 | 16 | -1.59 | 4.85E-03 | 7.43E-02 |
| NM_030908 | olfactory receptor, family 2, subfamily A, member 4 | Hs.444988 | 6q23 | OR2A4 | 98 | 12 | -1.59 | 6.92E-03 | 8.93E-02 |
| AK022204 | Homo sapiens, clone IMAGE:5314189, mRNA | Hs.288719 | 8p12 | FLJ23263 | 144 | 10 | -1.59 | 8.28E-03 | 9.69E-02 |
| AL136939 | ELOVL family member 5, elongation of long chain fatty acids (FEN1/Elo2, SUR4/Elo3-like, yeast) | Hs.343667 | 6p21.1-p12.1 | ELOVL5 | 1259 | 14 | -1.59 | 1.81E-03 | 4.64E-02 |
| AK021942 | Homo sapiens cDNA FLJ11880 fis, clone HEMBA1007129. | Hs.278564 | 17p13 | CLDN7 | 111 | 13 | -1.59 | 5.18E-03 | 7.62E-02 |
| NM_014505 | potassium large conductance calcium-activated channel, subfamily M, beta member 4 | Hs.348361 | 12q | KCNMB4 | 107 | 15 | -1.59 | 7.72E-03 | 9.43E-02 |
| NM_002826 | quiescin Q6 | Hs.77266 | 1q24 | QSCN6 | 2411 | 15 | -1.58 | 1.70E-03 | 4.49E-02 |
| AK058167 | hypothetical protein FLJ32940 | Hs.408184 | 1q24.3 | FLJ32940 | 712 | 12 | -1.58 | 6.51E-04 | 2.65E-02 |
| NM_001218 | carbonic anhydrase XII | Hs.279916 | 15q22 | CA12 | 267 | 16 | -1.58 | 7.82E-03 | 9.48E-02 |
| AK055431 | protein kinase C, alpha | Hs.349611 | 17q22-q23.2 | PRKCA | 1328 | 11 | -1.58 | 4.90E-03 | 7.43E-02 |
| BC015649 | hypothetical protein MGC23427 | Hs.244847 | 9q34.3 | MGC23427 | 337 | 15 | -1.58 | 5.11E-03 | 7.57E-02 |
| AK057729 | hypothetical protein FLJ33167 | Hs.351470 | 4q35.1 | FLJ33167 | 266 | 12 | -1.58 | 6.98E-03 | 8.98E-02 |
| NM_014718 | calsyntenin 3 | Hs.212151 | 12p13.31 | CLSTN3 | 687 | 16 | -1.58 | 1.03E-03 | 3.46E-02 |
| BC011620 | hypothetical protein MGC2668 | Hs.406421 | 9q34.13 | MGC2668 | 1019 | 15 | -1.58 | 2.84E-03 | 5.80E-02 |
| AB032988 | hypothetical protein DJ971N18.2 | Hs.169358 | 20p12 | DJ971N18.2 | 390 | 14 | -1.57 | 7.56E-03 | 9.34E-02 |
| NM_006526 | zinc finger protein 217 | Hs.155040 | 20q13.2 | ZNF217 | 1625 | 14 | -1.57 | 4.02E-03 | 6.84E-02 |
| NM_024653 | PRKR interacting protein 1 (IL11 inducible) | Hs.406395 | 7q22.1 | PRKRIP1 | 850 | 15 | -1.57 | 5.18E-03 | 7.62E-02 |
| NM_019048 | HCV NS3-transactivated protein 1 | Hs.101364 | 2p24.3-q21.3 | NS3TP1 | 745 | 14 | -1.57 | 3.95E-03 | 6.78E-02 |
| AK023065 | hypothetical protein LOC283075 | Hs.301533 | 10p12.31 | LOC283075 | 356 | 16 | -1.57 | 4.51E-03 | 7.19E-02 |
| AB018274 | likely ortholog of mouse la related protein | Hs.6214 | 5q33.2 | LARP | 2301 | 12 | -1.57 | 8.33E-03 | 9.70E-02 |
| AB046845 | E3 ubiquitin ligase SMURF1 | Hs.436249 | 7q21.1-q31.1 | SMURF1 | 749 | 16 | -1.57 | 1.05E-03 | 3.50E-02 |
| NM_032319 | chromosome 2 open reading frame 7 | Hs.61268 | 2p13.2 | C2orf7 | 741 | 15 | -1.57 | 3.66E-03 | 6.53E-02 |
| NM_017617 | Notch homolog 1, translocation-associated (Drosophila) | Hs.311559 | 9q34.3 | NOTCH1 | 1251 | 16 | -1.56 | 9.32E-04 | 3.25E-02 |
| NM_001120 | tetracycline transporter-like protein | Hs.157145 | 4p16.3 | TETRAN | 555 | 16 | -1.56 | 8.87E-03 | 9.98E-02 |
| AF170307 | Homo sapiens LOC347256 (LOC347256), mRNA | Hs.274173 | 13q13.1 | OIP2 | 307 | 15 | -1.56 | 4.40E-03 | 7.11E-02 |
| NM_015001 | SMART/HDAC1 associated repressor protein | Hs.184245 | 1p36.33-p36.11 | SHARP | 794 | 15 | -1.56 | 5.56E-03 | 7.94E-02 |
| AB033076 | likely homolog of rat kinase D-interacting substance of 220 kDa | Hs.9873 | 2p24 | KIDINS220 | 928 | 11 | -1.56 | 8.01E-03 | 9.61E-02 |
| AK055437 | KIAA0258 gene product | Hs.47313 | 9pter-p22.1 | KIAA0258 | 158 | 16 | -1.56 | 4.05E-03 | 6.84E-02 |
| AK001439 | hypothetical protein LOC257152 | Hs.208425 | 8q22.3 | LOC257152 | 565 | 16 | -1.56 | 5.75E-03 | 8.07E-02 |
| NM_005385 | natural killer-tumor recognition sequence | Hs.369815 | 3p23-p21 | NKTR | 1333 | 16 | -1.56 | 2.89E-03 | 5.82E-02 |
| BC016658 | hypothetical protein BC016658 | Hs.416375 | 12q21.1 | LOC144455 | 519 | 16 | -1.56 | 6.27E-04 | 2.57E-02 |
| AB033043 | hypothetical protein DKFZp761L0424 | Hs.189422 | 10p12.31 | DKFZP761L0424 | 300 | 16 | -1.55 | 3.08E-03 | 6.03E-02 |
| NM_024635 | Hypothetical protein FLJ22643 |  |  |  | 564 | 15 | -1.55 | 3.08E-03 | 6.03E-02 |
| BC014652 | hypothetical protein BC002942 | Hs.150540 | 22q13.33 | BC002942 | 1084 | 11 | -1.55 | 8.42E-03 | 9.74E-02 |
| NM_014755 | transcriptional regulator interacting with the PHS-bromodomain 2 | Hs.77293 | 2p15 | TRIP-Br2 | 4161 | 16 | -1.55 | 4.18E-03 | 6.99E-02 |
| AK026349 | hypothetical protein MGC45400 | Hs.389734 | Xq22.2 | MGC45400 | 626 | 14 | -1.55 | 4.97E-03 | 7.47E-02 |
| NM_006345 | solute carrier family 30 (zinc transporter), member 9 | Hs.364615 | 4p13-p12 | SLC30A9 | 2540 | 16 | -1.55 | 5.75E-03 | 8.06E-02 |
| AB037784 | KIAA1363 protein | Hs.22941 | 3q26.31-q26.32 | KIAA1363 | 2114 | 16 | -1.54 | 8.43E-03 | 9.73E-02 |
| NM_006754 | synaptophysin-like protein | Hs.80919 | 7q22.2 | SYPL | 2062 | 16 | -1.54 | 5.61E-03 | 7.98E-02 |
| AB037823 | KIAA1402 protein | Hs.86392 | 7q36.1 | CSGlcA-T | 2686 | 16 | -1.54 | 3.66E-03 | 6.54E-02 |
| NM_000034 | aldolase A, fructose-bisphosphate | Hs.273415 | 16q22-q24 | ALDOA | 13695 | 16 | -1.54 | 1.29E-03 | 3.89E-02 |
| AL161960 | chromosome 21 open reading frame 97 | Hs.458335 | 21q22.3 | C21orf97 | 1854 | 15 | -1.54 | 4.72E-03 | 7.35E-02 |
| AL133663 | Homo sapiens mRNA; cDNA DKFZp434O1521 (from clone DKFZp434O1521) |  |  |  | 298 | 16 | -1.54 | 5.68E-03 | 8.04E-02 |
| NM_001282 | adaptor-related protein complex 2, beta 1 subunit | Hs.370123 | 17q11.2-q12 | AP2B1 | 589 | 16 | -1.53 | 3.43E-03 | 6.36E-02 |
| NM_007195 | polymerase (DNA directed) iota | Hs.438533 | 18q21.1 | POLI | 121 | 13 | -1.53 | 8.31E-03 | 9.70E-02 |
| NM_005273 | guanine nucleotide binding protein (G protein), beta polypeptide 2 | Hs.185172 | 7q21.3-q22.1 | GNB2 | 4740 | 15 | -1.53 | 4.86E-03 | 7.43E-02 |
| AB051438 | Homo sapiens mRNA for KIAA1651 protein, partial cds. | Hs.469222 | 7q33 | FLJ20420 | 15726 | 15 | -1.53 | 8.26E-03 | 9.67E-02 |
| NM_032012 | chromosome 9 open reading frame 5 | Hs.418097 | 9q31 | C9orf5 | 1804 | 11 | -1.53 | 8.61E-03 | 9.87E-02 |
| AJ412032 | Homo sapiens mRNA for B-cell neoplasia associated transcript, (BCMS gene), splice variant J, non coding transcript | Hs.492218 | 8q13.3 | na | 447 | 16 | -1.53 | 5.47E-03 | 7.87E-02 |
| NM_017853 | hypothetical protein FLJ20511 | Hs.134406 | 16q22.2 | FLJ20511 | 1951 | 16 | -1.52 | 3.83E-03 | 6.67E-02 |
| NM_016294 | Protein phosphatase 6, catalytic subunit |  |  |  | 1219 | 15 | -1.52 | 1.46E-03 | 4.19E-02 |
| NM_017528 | Williams Beuren syndrome chromosome region 22 | Hs.413036 | 3q21 | WBSCR22 | 312 | 14 | -1.52 | 7.55E-03 | 9.34E-02 |
| NM_003253 | T-cell lymphoma invasion and metastasis 1 | Hs.115176 | 21q22.1 | TIAM1 | 167 | 15 | -1.51 | 4.24E-03 | 7.01E-02 |
| U18550 | G protein-coupled receptor 3 |  |  |  | 285 | 15 | -1.51 | 6.13E-03 | 8.36E-02 |
| NM_016053 | CGI-116 protein | Hs.18885 | 12q23.3 | CGI-116 | 797 | 15 | -1.51 | 8.24E-03 | 9.69E-02 |
| AL137534 | hypothetical protein DKFZp434H1419 | Hs.56876 | 2q35 | DKFZp434H1419 | 418 | 15 | -1.51 | 6.89E-03 | 8.91E-02 |
| AK000637 | HSPC043 protein | Hs.429892 | 9q32 | HSPC043 | 1456 | 16 | -1.50 | 3.15E-03 | 6.14E-02 |
| NM_016605 | chromosome 5 open reading frame 6 | Hs.54056 | 5q31 | C5orf6 | 1253 | 14 | 1.50 | 8.16E-03 | 9.65E-02 |
| NM_003094 | small nuclear ribonucleoprotein polypeptide E | Hs.334612 | 1q32 | SNRPE | 8257 | 16 | 1.50 | 3.68E-03 | 6.55E-02 |
| NM_006638 | ribonuclease P1 | Hs.511756 | 6p25.1 | RNASEP1 | 2450 | 16 | 1.50 | 3.59E-03 | 6.47E-02 |
| NM_015340 | leucyl-tRNA synthetase 2, mitochondrial | Hs.438303 | 3p21.3 | LARS2 | 303 | 16 | 1.51 | 4.46E-03 | 7.16E-02 |
| NM_004804 | WD40 protein Ciao1 | Hs.12109 | 2q11.2 | CIAO1 | 911 | 16 | 1.51 | 3.88E-03 | 6.72E-02 |
| BC010698 | similar to RIKEN cDNA 2600001A11 gene | Hs.22268 | 14q23.2 | LOC112840 | 241 | 16 | 1.51 | 7.90E-03 | 9.55E-02 |
| NM_031885 | Bardet-Biedl syndrome 2 | Hs.333738 | 16q21 | BBS2 | 425 | 13 | 1.51 | 6.73E-03 | 8.82E-02 |
| NM_019852 | methyltransferase like 3 | Hs.168799 | 14q11.1 | METTL3 | 1428 | 15 | 1.52 | 8.37E-03 | 9.71E-02 |
| NM_003276 | thymopoietin | Hs.11355 | 12q22 | TMPO | 822 | 16 | 1.52 | 7.58E-03 | 9.34E-02 |
| NM_012321 | LSM4 homolog, U6 small nuclear RNA associated (S. cerevisiae) | Hs.76719 | 19p13.11 | LSM4 | 3670 | 16 | 1.52 | 8.58E-03 | 9.84E-02 |
| NM_032280 | hypothetical protein DKFZp761J139 | Hs.15536 | 5q14.1 | DKFZp761J139 | 883 | 15 | 1.52 | 6.24E-03 | 8.43E-02 |
| AK024486 | glioma tumor suppressor candidate region gene 2 | Hs.421907 | 19q13.3 | GLTSCR2 | 1326 | 16 | 1.53 | 4.29E-03 | 7.05E-02 |
| BC001425 | P53-regulated DDA3 |  |  |  | 3138 | 16 | 1.53 | 2.12E-03 | 4.99E-02 |
| NM_001605 | alanyl-tRNA synthetase | Hs.315137 | 16q22 | AARS | 2594 | 16 | 1.53 | 5.26E-03 | 7.68E-02 |
| AF130082 | Homo sapiens clone FLC1492 PRO3121 mRNA, complete cds |  |  |  | 3954 | 16 | 1.54 | 2.84E-03 | 5.80E-02 |
| AF007128 | hypothetical protein MGC40195 | Hs.367956 | 12p13.33 | MGC40195 | 494 | 16 | 1.54 | 6.16E-03 | 8.38E-02 |
| NM_012155 | echinoderm microtubule associated protein like 2 | Hs.24178 | 19q13.32 | EML2 | 206 | 14 | 1.54 | 8.60E-03 | 9.86E-02 |
| NM_003860 | barrier to autointegration factor 1 | Hs.433759 | 11q13.1 | BANF1 | 2448 | 16 | 1.54 | 5.48E-03 | 7.87E-02 |
| NM_021727 | fatty acid desaturase 3 | Hs.21765 | 11q12-q13.1 | FADS3 | 1136 | 16 | 1.54 | 2.26E-03 | 5.17E-02 |
| NM_002845 | protein tyrosine phosphatase, receptor type, M | Hs.154151 | 18p11.2 | PTPRM | 412 | 11 | 1.54 | 7.31E-03 | 9.19E-02 |
| NM_000521 | hexosaminidase B (beta polypeptide) | Hs.69293 | 5q13 | HEXB | 1884 | 15 | 1.55 | 6.81E-03 | 8.84E-02 |
| AK026551 | gamma tubulin ring complex protein (76p gene) | Hs.497941 | 15q15 | 76P | 577 | 16 | 1.55 | 2.51E-03 | 5.42E-02 |
| NM_003920 | timeless homolog (Drosophila) | Hs.118631 | 12q12-q13 | TIMELESS | 991 | 16 | 1.55 | 5.71E-03 | 8.04E-02 |
| NM_013241 | formin homology 2 domain containing 1 | Hs.95231 | 16q22 | FHOD1 | 707 | 14 | 1.55 | 3.98E-03 | 6.79E-02 |
| NM_003580 | neutral sphingomyelinase (N-SMase) activation associated factor | Hs.372000 | 8q12-q13 | NSMAF | 1097 | 16 | 1.55 | 2.72E-03 | 5.66E-02 |
| AK025019 | decapping enzyme hDcp2 | Hs.442039 | 5q22.3 | DCP2 | 1204 | 15 | 1.56 | 2.86E-03 | 5.80E-02 |
| NM_018060 | hypothetical protein FLJ10326 | Hs.262823 | 1q42.11 | FLJ10326 | 1660 | 16 | 1.56 | 8.11E-03 | 9.63E-02 |
| AB046809 | zinc finger, FYVE domain containing 1 | Hs.368384 | 14q22-q24 | ZFYVE1 | 287 | 15 | 1.57 | 6.77E-03 | 8.82E-02 |
| NM_003748 | aldehyde dehydrogenase 4 family, member A1 | Hs.77448 | 1p36 | ALDH4A1 | 549 | 16 | 1.57 | 5.96E-03 | 8.22E-02 |
| AL137663 | LL5 beta | Hs.7378 | 3q13.13 | LL5beta | 1119 | 13 | 1.57 | 4.69E-03 | 7.34E-02 |
| NM_006548 | IGF-II mRNA-binding protein 2 | Hs.30299 | 3q28 | IMP-2 | 652 | 16 | 1.57 | 5.19E-03 | 7.62E-02 |
| NM_024660 | hypothetical protein FLJ22573 | Hs.352548 | 19q13.13 | FLJ22573 | 123 | 14 | 1.57 | 5.62E-03 | 7.99E-02 |
| AF241785 | KIAA1128 protein | Hs.81897 | 10q23.2 | KIAA1128 | 1590 | 14 | 1.57 | 5.43E-03 | 7.85E-02 |
| NM_017916 | hypothetical protein FLJ20643 | Hs.5245 | 19q13.33 | FLJ20643 | 1996 | 16 | 1.57 | 2.21E-03 | 5.10E-02 |
| BC015582 | hypothetical protein MGC23280 | Hs.5163 | 17q11.2 | MGC23280 | 550 | 15 | 1.57 | 7.94E-03 | 9.56E-02 |
| NM_032834 | alpha2-glucosyltransferase | Hs.102971 | 12p11.21 | ALG10 | 142 | 11 | 1.57 | 3.55E-03 | 6.44E-02 |
| NM_006023 | chromosome 10 open reading frame 7 | Hs.412842 | 10p13 | C10orf7 | 8926 | 16 | 1.57 | 6.11E-03 | 8.35E-02 |
| NM_022897 | RAN binding protein 17 | Hs.410810 | 5q34 | RANBP17 | 409 | 15 | 1.58 | 2.30E-03 | 5.20E-02 |
| NM_004640 | HLA-B associated transcript 1 | Hs.254042 | 6p21.3 | BAT1 | 3262 | 16 | 1.58 | 2.10E-03 | 4.97E-02 |
| NM_006779 | CDC42 effector protein (Rho GTPase binding) 2 | Hs.12289 | 11q13 | CDC42EP2 | 1092 | 15 | 1.58 | 1.56E-03 | 4.29E-02 |
| AF136408 | Chromosome 6 open reading frame 4 |  |  |  | 179 | 15 | 1.58 | 4.97E-03 | 7.47E-02 |
| NM_001009 | ribosomal protein S5 | Hs.378103 | 19q13.4 | RPS5 | 13032 | 16 | 1.58 | 7.22E-03 | 9.12E-02 |
| NM_006441 | 5,10-methenyltetrahydrofolate synthetase (5-formyltetrahydrofolate cyclo-ligase) | Hs.118131 | 15q24.3 | MTHFS | 2351 | 16 | 1.58 | 4.51E-03 | 7.20E-02 |
| NM_032485 | MCM8 minichromosome maintenance deficient 8 (S. cerevisiae) | Hs.134278 | 20p12.3 | MCM8 | 532 | 14 | 1.59 | 7.88E-03 | 9.54E-02 |
| NM_006764 | interferon-related developmental regulator 2 | Hs.315177 | 3p21.3 | IFRD2 | 1061 | 16 | 1.59 | 3.89E-03 | 6.72E-02 |
| AL353681 | Human DNA sequence from clone RP1-158P9 on chromosome 1. Contains a putative novel gene, a laminin r |  |  |  | 2378 | 14 | 1.59 | 8.11E-03 | 9.63E-02 |
| NM_005567 | lectin, galactoside-binding, soluble, 3 binding protein | Hs.79339 | 17q25 | LGALS3BP | 3367 | 15 | 1.59 | 1.22E-03 | 3.79E-02 |
| NM_022662 | anaphase-promoting complex 1 (meiotic checkpoint regulator) | Hs.436527 | 2q12.1 | ANAPC1 | 1818 | 15 | 1.59 | 8.88E-03 | 9.98E-02 |
| NM_003447 | zinc finger protein 165 | Hs.55481 | 6p21.3 | ZNF165 | 311 | 15 | 1.59 | 6.90E-03 | 8.90E-02 |
| NM_016089 | KRAB-zinc finger protein SZF1-1 | Hs.19585 | 3p21 | SZF1 | 306 | 10 | 1.59 | 8.69E-03 | 9.86E-02 |
| AB018268 | KIAA0725 protein | Hs.434966 | 8p11.23 | KIAA0725 | 699 | 16 | 1.60 | 1.15E-04 | 9.64E-03 |
| NM_005573 | lamin B1 | Hs.89497 | 5q23.3-q31.1 | LMNB1 | 1708 | 15 | 1.60 | 4.31E-03 | 7.05E-02 |
| NM_022553 | vacuolar protein sorting 52 (yeast) | Hs.433731 | 6p21.3 | VPS52 | 746 | 15 | 1.60 | 3.66E-03 | 6.52E-02 |
| BC003376 | ELAV (embryonic lethal, abnormal vision, Drosophila)-like 1 (Hu antigen R) | Hs.184492 | 19p13.2 | ELAVL1 | 1805 | 16 | 1.60 | 6.34E-03 | 8.48E-02 |
| AF090094 | ornithine decarboxylase antizyme 1 | Hs.446427 | 19p13.3 | OAZ1 | 3043 | 13 | 1.60 | 8.68E-03 | 9.88E-02 |
| NM_000987 | ribosomal protein L26 | Hs.406682 | 17p13 | RPL26 | 12000 | 16 | 1.60 | 8.35E-03 | 9.72E-02 |
| NM_012459 | translocase of inner mitochondrial membrane 8 homolog B (yeast) | Hs.279915 | 11q23.1-q23.2 | TIMM8B | 3374 | 16 | 1.60 | 6.24E-03 | 8.42E-02 |
| NM_015684 | ATP synthase, H+ transporting, mitochondrial F0 complex, subunit s (factor B) | Hs.440456 | 14q22.1 | ATP5S | 337 | 16 | 1.60 | 2.05E-03 | 4.89E-02 |
| NM_003826 | N-ethylmaleimide-sensitive factor attachment protein, gamma | Hs.370431 | 18p11.21 | NAPG | 573 | 15 | 1.60 | 4.55E-03 | 7.22E-02 |
| NM_015510 | DKFZP566O084 protein | Hs.11411 | 17p12 | DKFZp566O084 | 1265 | 16 | 1.60 | 1.47E-03 | 4.18E-02 |
| AF339822 | fms-related tyrosine kinase 1 (vascular endothelial growth factor/vascular permeability factor receptor) | Hs.347713 | 13q12 | FLT1 | 389 | 15 | 1.61 | 1.30E-03 | 3.92E-02 |
| NM_000919 | peptidylglycine alpha-amidating monooxygenase | Hs.352733 | 5q14-q21 | PAM | 2048 | 16 | 1.61 | 1.62E-03 | 4.39E-02 |
| NM_017917 | chromosome 14 open reading frame 10 | Hs.419151 | 14q13.2 | C14orf10 | 659 | 16 | 1.61 | 1.42E-03 | 4.13E-02 |
| NM_003574 | VAMP (vesicle-associated membrane protein)-associated protein A, 33kDa | Hs.165195 | 18p11.22 | VAPA | 1341 | 16 | 1.61 | 6.77E-03 | 8.82E-02 |
| NM_032866 | hypothetical protein FLJ14957 | Hs.10119 | 15q21.2 | FLJ14957 | 2697 | 16 | 1.61 | 1.15E-03 | 3.66E-02 |
| AK055846 | hypothetical protein MGC25181 | Hs.511975 | 2q37.3 | MGC25181 | 347 | 14 | 1.61 | 4.87E-03 | 7.43E-02 |
| NM_005466 | mediator of RNA polymerase II transcription, subunit 6 homolog (yeast) | Hs.167738 | 14q24.1 | MED6 | 844 | 12 | 1.61 | 6.23E-03 | 8.42E-02 |
| AK057753 | Homo sapiens RGS8 mRNA, partial sequence | Hs.20982 | 20q12 | GDAP1L1 | 212 | 16 | 1.61 | 4.94E-03 | 7.47E-02 |
| NM_014297 | protein expressed in thyroid | Hs.7486 | 19q13.32 | YF13H12 | 1572 | 15 | 1.62 | 2.40E-03 | 5.32E-02 |
| NM_001500 | GDP-mannose 4,6-dehydratase | Hs.105435 | 6p25 | GMDS | 889 | 16 | 1.62 | 7.43E-03 | 9.27E-02 |
| NM_024297 | hypothetical protein MGC2941 | Hs.380734 | 17p13.2 | MGC2941 | 2121 | 9 | 1.62 | 4.19E-03 | 6.99E-02 |
| NM_022362 | MMS19-like (MET18 homolog, S. cerevisiae) | Hs.288891 | 10q24-q25 | MMS19L | 713 | 15 | 1.62 | 5.37E-03 | 7.79E-02 |
| NM_031210 | hypothetical protein DC50 | Hs.324521 | 14q24.3 | DC50 | 2661 | 14 | 1.62 | 4.73E-03 | 7.35E-02 |
| NM_033180 | olfactory receptor, family 51, subfamily B, member 2 | Hs.283879 | 11p15 | OR51B2 | 373 | 13 | 1.63 | 5.70E-03 | 8.03E-02 |
| AB051487 | dual specificity phosphatase 16 | Hs.20281 | 12p13 | DUSP16 | 627 | 16 | 1.63 | 2.96E-03 | 5.91E-02 |
| NM_016083 | cannabinoid receptor 1 (brain) | Hs.75110 | 6q14-q15 | CNR1 | 1619 | 15 | 1.63 | 7.06E-03 | 9.02E-02 |
| AF070587 | KIAA1509 protein | Hs.25770 | 14q32.12 | KIAA1509 | 128 | 10 | 1.63 | 6.75E-03 | 8.82E-02 |
| NM_005581 | Lutheran blood group (Auberger b antigen included) | Hs.155048 | 19q13.2 | LU | 568 | 15 | 1.63 | 8.25E-03 | 9.69E-02 |
| NM_032490 | chromosome 14 open reading frame 142 | Hs.20142 | 14q32.13 | C14orf142 | 1318 | 16 | 1.63 | 3.98E-03 | 6.80E-02 |
| BF698890 | proteasome (prosome, macropain) subunit, beta type, 6 | Hs.77060 | 17p13 | PSMB6 | 1158 | 14 | 1.63 | 4.76E-03 | 7.35E-02 |
| NM_022831 | hypothetical protein FLJ12806 | Hs.512696 | 1q42.12 | FLJ12806 | 1245 | 12 | 1.64 | 6.49E-03 | 8.61E-02 |
| AF109161 | Cbp/p300-interacting transactivator, with Glu/Asp-rich carboxy-terminal domain, 2 | Hs.82071 | 6q23.3 | CITED2 | 1911 | 16 | 1.64 | 8.68E-03 | 9.89E-02 |
| NM_004120 | guanylate binding protein 2, interferon-inducible | Hs.386567 | 1p22.2 | GBP2 | 1275 | 15 | 1.64 | 6.07E-03 | 8.32E-02 |
| NM_017941 | hypothetical protein FLJ20721 | Hs.12929 | 17q25.1 | FLJ20721 | 326 | 14 | 1.65 | 8.38E-04 | 3.07E-02 |
| NM_007271 | serine/threonine kinase 38 | Hs.367811 | 6p21 | STK38 | 799 | 14 | 1.65 | 1.82E-03 | 4.67E-02 |
| NM_005443 | 3'-phosphoadenosine 5'-phosphosulfate synthase 1 | Hs.3833 | 4q24 | PAPSS1 | 381 | 11 | 1.65 | 6.25E-03 | 8.42E-02 |
| NM_022908 | hypothetical protein FLJ12442 | Hs.84753 | 3p21.31 | FLJ12442 | 3847 | 16 | 1.65 | 3.54E-03 | 6.44E-02 |
| NM_021618 | RNA binding motif protein 8A |  |  |  | 872 | 14 | 1.65 | 2.80E-03 | 5.77E-02 |
| NM_001270 | chromodomain helicase DNA binding protein 1 | Hs.311553 | 5q15-q21 | CHD1 | 1460 | 16 | 1.65 | 1.73E-03 | 4.55E-02 |
| NM_016256 | N-acetylglucosamine-1-phosphodiester alpha-N-acetylglucosaminidase | Hs.21334 | 16p13.3 | NAGPA | 337 | 13 | 1.65 | 4.21E-03 | 7.00E-02 |
| NM_016576 | guanosine monophosphate reductase 2 | Hs.368855 | 14q11.2 | GMPR2 | 277 | 15 | 1.65 | 4.38E-03 | 7.12E-02 |
| NM_006995 | butyrophilin, subfamily 2, member A2 | Hs.91813 | 6p22.1 | BTN2A2 | 192 | 14 | 1.65 | 8.85E-03 | 9.97E-02 |
| NM_005507 | cofilin 1 (non-muscle) | Hs.170622 | 11q13 | CFL1 | 14549 | 16 | 1.65 | 1.46E-03 | 4.19E-02 |
| AK001921 | zinc finger, FYVE domain containing 21 | Hs.169575 | 14q32.33 | ZFYVE21 | 691 | 16 | 1.66 | 2.94E-03 | 5.88E-02 |
| NM_014160 | makorin, ring finger protein, 2 | Hs.279474 | 3p25 | MKRN2 | 363 | 14 | 1.66 | 5.70E-03 | 8.04E-02 |
| NM_000274 | ornithine aminotransferase (gyrate atrophy) | Hs.75485 | 10q26 | OAT | 2751 | 15 | 1.67 | 5.34E-03 | 7.76E-02 |
| BC014318 | Homo sapiens, clone IMAGE:3684608, mRNA | Hs.298058 | 12q13 | AQP5 | 87 | 7 | 1.67 | 6.58E-03 | 8.69E-02 |
| NM_024339 | hypothetical protein MGC2655 | Hs.412304 | 16p13.3 | MGC2655 | 304 | 16 | 1.67 | 4.90E-03 | 7.44E-02 |
| AK054678 | hypothetical protein DKFZp761G058 | Hs.291000 | 4q22.1 | DKFZp761G058 | 331 | 14 | 1.67 | 8.62E-03 | 9.87E-02 |
| NM_001966 | enoyl-Coenzyme A, hydratase/3-hydroxyacyl Coenzyme A dehydrogenase | Hs.432443 | 3q26.3-q28 | EHHADH | 234 | 12 | 1.67 | 5.62E-03 | 7.99E-02 |
| NM_001491 | glucosaminyl (N-acetyl) transferase 2, I-branching enzyme | Hs.934 | 6p24 | GCNT2 | 3081 | 15 | 1.67 | 2.33E-03 | 5.25E-02 |
| NM_007278 | GABA(A) receptor-associated protein | Hs.381102 | 17p13.2 | GABARAP | 2277 | 15 | 1.67 | 2.30E-04 | 1.39E-02 |
| NM_020441 | coronin, actin binding protein, 1B | Hs.6191 | 11q13.1 | CORO1B | 4704 | 16 | 1.68 | 2.96E-03 | 5.90E-02 |
| NM_001254 | CDC6 cell division cycle 6 homolog (S. cerevisiae) | Hs.405958 | 17q21.3 | CDC6 | 1860 | 15 | 1.68 | 8.25E-03 | 9.68E-02 |
| NM_017572 | MAP kinase-interacting serine/threonine kinase 2 | Hs.512094 | 19p13.3 | MKNK2 | 3868 | 16 | 1.68 | 2.18E-03 | 5.07E-02 |
| NM_006622 | serum-inducible kinase | Hs.398157 | 5q12.1-q13.2 | SNK | 9353 | 16 | 1.68 | 5.19E-04 | 2.29E-02 |
| NM_017955 | cell division cycle associated 4 | Hs.34045 | 14q32.33 | CDCA4 | 576 | 16 | 1.68 | 1.13E-03 | 3.62E-02 |
| NM_014736 | KIAA0101 gene product | Hs.81892 | 15q22.1 | KIAA0101 | 5879 | 14 | 1.68 | 6.09E-04 | 2.51E-02 |
| BC001830 | transforming growth factor beta 1 induced transcript 1 | Hs.25511 | 16p11.2 | TGFB1I1 | 188 | 10 | 1.68 | 1.84E-03 | 4.66E-02 |
| NM_003384 | vaccinia related kinase 1 | Hs.422662 | 14q32 | VRK1 | 515 | 15 | 1.68 | 5.78E-03 | 8.09E-02 |
| NM_019117 | kelch-like 4 (Drosophila) | Hs.49075 | Xq21.3 | KLHL4 | 575 | 16 | 1.68 | 3.72E-03 | 6.58E-02 |
| NM_018019 | hypothetical protein FLJ10193 | Hs.235195 | 17p11.2 | FLJ10193 | 165 | 14 | 1.69 | 5.74E-03 | 8.06E-02 |
| NM_006471 | myosin regulatory light chain MRCL3 | Hs.233936 | 18p11.31 | MRCL3 | 4851 | 14 | 1.69 | 9.89E-04 | 3.38E-02 |
| NM_003876 | chromosome 17 open reading frame 35 | Hs.15196 | 17p11.2 | C17orf35 | 2572 | 16 | 1.69 | 2.34E-03 | 5.26E-02 |
| NM_021188 | likely ortholog of mouse another partner for ARF 1 | Hs.405945 | 14q24.3 | APA1 | 1387 | 12 | 1.69 | 3.69E-04 | 1.85E-02 |
| NM_003413 | Zic family member 3 heterotaxy 1 (odd-paired homolog, Drosophila) | Hs.111227 | Xq26.2 | ZIC3 | 532 | 15 | 1.69 | 3.17E-04 | 1.75E-02 |
| AL049962 | hypothetical protein LOC286148 | Hs.165539 | 8q22.1 | LOC286148 | 1116 | 14 | 1.69 | 4.31E-03 | 7.05E-02 |
| NM_006903 | inorganic pyrophosphatase 2 | Hs.421825 | 4q25 | PPA2 | 2251 | 16 | 1.69 | 4.48E-04 | 2.08E-02 |
| NM_024576 | hypothetical protein FLJ21079 | Hs.16512 | 6q13 | FLJ21079 | 2765 | 16 | 1.69 | 1.54E-03 | 4.28E-02 |
| NM_002796 | proteasome (prosome, macropain) subunit, beta type, 4 | Hs.89545 | 1q21 | PSMB4 | 2167 | 16 | 1.69 | 8.28E-03 | 9.68E-02 |
| NM_032525 | tubulin beta-5 | Hs.274398 | 18p11.21 | TUBB-5 | 4110 | 15 | 1.69 | 2.34E-04 | 1.40E-02 |
| NM_016355 | DEAD (Asp-Glu-Ala-Asp) box polypeptide 47 | Hs.284288 | 12p13.2 | DDX47 | 509 | 16 | 1.69 | 8.53E-03 | 9.82E-02 |
| NM_002828 | protein tyrosine phosphatase, non-receptor type 2 | Hs.446126 | 18p11.3-p11.2 | PTPN2 | 970 | 13 | 1.69 | 4.59E-03 | 7.25E-02 |
| NM_016048 | CGI-111 protein | Hs.11085 | 5q22.1-q33.3 | CGI-111 | 1589 | 16 | 1.70 | 5.96E-04 | 2.47E-02 |
| NM_005037 | peroxisome proliferative activated receptor, gamma | Hs.387667 | 3p25 | PPARG | 643 | 16 | 1.70 | 4.65E-03 | 7.31E-02 |
| NM_004824 | chromodomain protein, Y-like | Hs.16081 | 6p25.1 | CDYL | 837 | 15 | 1.70 | 3.30E-03 | 6.23E-02 |
| BC013181 | hypothetical protein LOC201292 | Hs.189823 | 17q25.3 | LOC201292 | 157 | 15 | 1.70 | 2.49E-03 | 5.41E-02 |
| NM_000365 | triosephosphate isomerase 1 | Hs.512711 | 12p13 | TPI1 | 7823 | 15 | 1.70 | 1.96E-03 | 4.81E-02 |
| AB014564 | KIAA0664 protein | Hs.22616 | 17p13.3 | KIAA0664 | 4521 | 16 | 1.70 | 4.33E-04 | 2.06E-02 |
| NM_003453 | zinc finger protein 198 | Hs.315241 | 13q11-q12 | ZNF198 | 424 | 12 | 1.70 | 1.63E-03 | 4.40E-02 |
| AK057232 | hypothetical protein FLJ32670 | Hs.345877 | 18q21.1 | FLJ32670 | 426 | 13 | 1.70 | 1.30E-03 | 3.90E-02 |
| NM_004886 | amyloid beta (A4) precursor protein-binding, family A, member 3 (X11-like 2) | Hs.17528 | 19p13.3 | APBA3 | 161 | 12 | 1.71 | 4.05E-03 | 6.84E-02 |
| AB033092 | likely ortholog of mouse metastasis associated 3 | Hs.435413 | 2p22.1 | MTA3 | 323 | 16 | 1.71 | 2.03E-03 | 4.90E-02 |
| BC015422 | ankyrin repeat domain 9 | Hs.432945 | 14q32.33 | ANKRD9 | 423 | 14 | 1.71 | 7.69E-03 | 9.43E-02 |
| AK021799 | RNA binding protein S1, serine-rich domain | Hs.355643 | 16p13.3 | RNPS1 | 1023 | 15 | 1.71 | 3.53E-03 | 6.44E-02 |
| BC008335 | KIAA1395 protein | Hs.8982 | 19p13.2 | KIAA1395 | 189 | 10 | 1.71 | 7.27E-03 | 9.16E-02 |
| NM_015462 | DKFZP586L0724 protein | Hs.26761 | 17q24.3 | DKFZP586L0724 | 570 | 15 | 1.71 | 6.82E-04 | 2.69E-02 |
| NM_005151 | ubiquitin specific protease 14 (tRNA-guanine transglycosylase) | Hs.75981 | 18p11.32 | USP14 | 6228 | 13 | 1.71 | 4.88E-03 | 7.43E-02 |
| NM_006470 | tripartite motif-containing 16 | Hs.241305 | 17p11.2 | TRIM16 | 2567 | 16 | 1.71 | 3.23E-03 | 6.20E-02 |
| NM_031902 | mitochondrial ribosomal protein S5 | Hs.387988 | 2p11.2-q11.2 | MRPS5 | 1452 | 16 | 1.71 | 1.72E-03 | 4.53E-02 |
| AB058750 | Hypothetical protein FLJ14972 |  |  |  | 3155 | 14 | 1.72 | 1.35E-03 | 4.01E-02 |
| NM_016622 | mitochondrial ribosomal protein L35 | Hs.433439 | 2p11.2 | MRPL35 | 1849 | 15 | 1.72 | 3.58E-03 | 6.46E-02 |
| NM_012261 | chromosome 20 open reading frame 103 | Hs.22920 | 20p12 | C20orf103 | 1805 | 15 | 1.72 | 5.31E-04 | 2.32E-02 |
| AK024136 | nuclear receptor co-repressor 1 | Hs.144904 | 17p11.2 | NCOR1 | 784 | 14 | 1.72 | 7.13E-03 | 9.06E-02 |
| NM_003244 | TGFB-induced factor (TALE family homeobox) | Hs.161999 | 18p11.3 | TGIF | 1477 | 16 | 1.72 | 5.02E-03 | 7.50E-02 |
| NM_002767 | phosphoribosyl pyrophosphate synthetase-associated protein 2 | Hs.13339 | 17p11.2-p12 | PRPSAP2 | 1174 | 16 | 1.72 | 1.51E-03 | 4.24E-02 |
| AL117425 | TIGA1 | Hs.12082 | 5q21-q22 | TIGA1 | 1422 | 13 | 1.72 | 4.97E-04 | 2.22E-02 |
| NM_001013 | ribosomal protein S9 | Hs.139876 | 19q13.4 | RPS9 | 14007 | 16 | 1.72 | 6.93E-03 | 8.93E-02 |
| NM_006331 | C2f protein | Hs.135643 | 12p13 | C2F | 7334 | 16 | 1.72 | 5.96E-05 | 6.80E-03 |
| AL080062 | DKFZP564I122 protein | Hs.13024 | 1p34.1 | DKFZP564I122 | 1677 | 16 | 1.73 | 3.23E-04 | 1.77E-02 |
| AL136640 | polyadenylate-binding protein-interacting protein 2 | Hs.396644 | 5q31.3 | PAIP2 | 1149 | 14 | 1.73 | 5.78E-03 | 8.09E-02 |
| AK022325 | hypothetical protein FLJ10613 | Hs.438583 | Xp11.22 | FLJ10613 | 437 | 14 | 1.73 | 3.01E-03 | 5.95E-02 |
| AB046810 | chromosome 20 open reading frame 23 | Hs.101774 | 20p11.23 | C20orf23 | 152 | 12 | 1.73 | 7.18E-03 | 9.10E-02 |
| NM_003284 | transition protein 1 (during histone to protamine replacement) | Hs.3017 | 2q35-q36 | TNP1 | 218 | 16 | 1.73 | 5.00E-03 | 7.47E-02 |
| NM_018477 | actin-related protein 10 homolog (S. cerevisiae) | Hs.248569 | 14q22.3 | ACTR10 | 1333 | 16 | 1.74 | 6.44E-03 | 8.58E-02 |
| NM_016080 | CGI-150 protein | Hs.279061 | 17p13.3 | CGI-150 | 1135 | 15 | 1.74 | 5.92E-03 | 8.22E-02 |
| NM_032361 | THO complex 3 | Hs.19574 | 5q35.3 | THOC3 | 2003 | 16 | 1.74 | 4.86E-03 | 7.43E-02 |
| AF225985 | Sodium channel, voltage-gated, type I, alpha polypeptide |  |  |  | 272 | 11 | 1.74 | 6.26E-03 | 8.41E-02 |
| AK022071 | Homo sapiens cDNA FLJ12009 fis, clone HEMBB1001618 |  |  |  | 4382 | 15 | 1.74 | 4.07E-03 | 6.86E-02 |
| BC008201 | polyposis locus protein 1-like 1 | Hs.76277 | 19p13.3 | DP1L1 | 264 | 16 | 1.75 | 3.51E-04 | 1.83E-02 |
| NM_031965 | haspin | Hs.193666 | 17p13 | GSG2 | 185 | 11 | 1.75 | 3.76E-03 | 6.63E-02 |
| NM_002696 | polymerase (RNA) II (DNA directed) polypeptide G | Hs.14839 | 11q13.1 | POLR2G | 1124 | 14 | 1.75 | 6.56E-04 | 2.64E-02 |
| NM_014426 | sorting nexin 5 | Hs.13794 | 20p11 | SNX5 | 2131 | 16 | 1.75 | 1.63E-03 | 4.40E-02 |
| NM_004092 | enoyl Coenzyme A hydratase, short chain, 1, mitochondrial | Hs.76394 | 10q26.2-q26.3 | ECHS1 | 6041 | 15 | 1.75 | 2.08E-04 | 1.31E-02 |
| NM_007273 | repressor of estrogen receptor activity | Hs.444499 | 12p13 | REA | 3908 | 16 | 1.75 | 9.62E-05 | 8.68E-03 |
| NM_018121 | chromosome 10 open reading frame 6 | Hs.447458 | 10q24.32 | C10orf6 | 430 | 14 | 1.75 | 4.52E-03 | 7.20E-02 |
| NM_005083 | signal recognition particle 19kDa | Hs.2943 | 5q21-q22 | SRP19 | 477 | 16 | 1.75 | 1.54E-03 | 4.28E-02 |
| NM_004347 | caspase 5, apoptosis-related cysteine protease | Hs.213327 | 11q22.2-q22.3 | CASP5 | 12278 | 13 | 1.75 | 8.31E-04 | 3.07E-02 |
| AL389943 | Homo sapiens mRNA full length insert cDNA clone EUROIMAGE 2005779 |  |  |  | 1656 | 16 | 1.75 | 1.48E-04 | 1.11E-02 |
| NM_012338 | transmembrane 4 superfamily member 12 | Hs.16529 | 7q31.31 | TM4SF12 | 692 | 13 | 1.75 | 1.80E-03 | 4.63E-02 |
| NM_018444 | protein phosphatase 2C, magnesium-dependent, catalytic subunit | Hs.22265 | 8q22.1 | PPM2C | 939 | 16 | 1.75 | 1.56E-04 | 1.13E-02 |
| AK027745 | WD repeat domain 21 | Hs.331491 | 14q24.3 | WDR21 | 425 | 15 | 1.75 | 3.16E-03 | 6.13E-02 |
| NM_006305 | acidic (leucine-rich) nuclear phosphoprotein 32 family, member A | Hs.356089 | 15q22.3-q23 | ANP32A | 511 | 16 | 1.75 | 2.55E-03 | 5.48E-02 |
| AJ243706 | putative DNA/chromatin binding motif | Hs.143323 | 1q32.1 | PLU-1 | 239 | 14 | 1.75 | 6.60E-03 | 8.70E-02 |
| NM_004713 | serologically defined colon cancer antigen 1 | Hs.388584 | 14q22 | SDCCAG1 | 409 | 11 | 1.76 | 4.87E-03 | 7.43E-02 |
| AL110249 | chromosome 20 open reading frame 194 | Hs.119021 | 20p13 | C20orf194 | 123 | 14 | 1.76 | 3.40E-03 | 6.32E-02 |
| NM_014216 | inositol 1,3,4-triphosphate 5/6 kinase | Hs.408429 | 14q31 | ITPK1 | 567 | 13 | 1.76 | 3.26E-03 | 6.22E-02 |
| NM_002936 | ribonuclease H1 | Hs.511960 | 2p25 | RNASEH1 | 7451 | 10 | 1.77 | 7.00E-03 | 8.99E-02 |
| AK027813 | Hypothetical protein MGC10744 |  |  |  | 475 | 14 | 1.77 | 1.23E-03 | 3.78E-02 |
| NM_006109 | SKB1 homolog (S. pombe) | Hs.367854 | 14q11.2-q21 | SKB1 | 1142 | 12 | 1.77 | 6.63E-03 | 8.71E-02 |
| NM_006311 | nuclear receptor co-repressor 1 | Hs.144904 | 17p11.2 | NCOR1 | 450 | 16 | 1.77 | 5.02E-04 | 2.23E-02 |
| NM_004897 | multiple inositol polyphosphate histidine phosphatase, 1 | Hs.95907 | 10q23 | MINPP1 | 148 | 14 | 1.77 | 1.56E-03 | 4.29E-02 |
| AL445468 | Human DNA sequence from clone RP11-74P14 on chromosome 1 Contains part of a novel gene, a novel gene |  |  |  | 1029 | 16 | 1.78 | 7.47E-04 | 2.89E-02 |
| NM_001326 | cleavage stimulation factor, 3' pre-RNA, subunit 3, 77kDa | Hs.180034 | 11p13 | CSTF3 | 704 | 15 | 1.78 | 4.23E-04 | 2.03E-02 |
| AF161415 | selenoprotein K | Hs.58471 | 3p21.31 | SELK | 3213 | 13 | 1.78 | 4.30E-03 | 7.05E-02 |
| NM_002220 | inositol 1,4,5-trisphosphate 3-kinase A | Hs.2722 | 15q14-q21 | ITPKA | 601 | 16 | 1.78 | 1.94E-04 | 1.27E-02 |
| NM_000752 | Leukotriene b4 receptor (chemokine receptor-like 1) |  |  |  | 882 | 15 | 1.79 | 2.08E-03 | 4.96E-02 |
| AK055603 | zinc finger protein 84 (HPF2) | Hs.22664 | 12q24.33 | ZNF84 | 887 | 12 | 1.79 | 2.55E-03 | 5.48E-02 |
| AB067480 | G protein-regulated inducer of neurite outgrowth 1 | Hs.150549 | 5q35.3 | KIAA1893 | 277 | 12 | 1.79 | 7.57E-03 | 9.35E-02 |
| AB037783 | hypothetical protein FLJ11183 | Hs.170623 | 12q23.1 | FLJ11183 | 573 | 15 | 1.79 | 6.06E-03 | 8.32E-02 |
| NM_004483 | glycine cleavage system protein H (aminomethyl carrier) | Hs.513762 | 1q23.3 | GCSH | 1194 | 16 | 1.79 | 1.90E-03 | 4.74E-02 |
| NM_000313 | protein S (alpha) | Hs.64016 | 3p11-q11.2 | PROS1 | 1694 | 15 | 1.80 | 6.56E-04 | 2.64E-02 |
| AK027408 | TRAF4 associated factor 1 | Hs.181466 | 15q14 | FLJ14502 | 590 | 15 | 1.80 | 3.15E-03 | 6.14E-02 |
| NM_032382 | component of oligomeric golgi complex 8 | Hs.130849 | 16q22.1 | COG8 | 343 | 15 | 1.80 | 2.77E-03 | 5.73E-02 |
| NM_006397 | ribonuclease H2, large subunit | Hs.25292 | 19p13.2 | RNASEH2A | 1065 | 15 | 1.80 | 3.83E-03 | 6.67E-02 |
| AL050353 | Opa-interacting protein 2 | Hs.274170 | 13q13.1 | OIP2 | 556 | 13 | 1.80 | 1.91E-03 | 4.74E-02 |
| AK056000 | hypothetical protein FLJ31438 | Hs.24423 | 2p16.3 | FLJ31438 | 511 | 14 | 1.80 | 5.18E-03 | 7.62E-02 |
| NM_018244 | chromosome 20 open reading frame 44 | Hs.176950 | 20q11.23 | C20orf44 | 1413 | 15 | 1.80 | 7.54E-03 | 9.35E-02 |
| AK025184 | Homo sapiens cDNA: FLJ21531 fis, clone COL06036 | Hs.102941 | 3p21 | AF3P21 | 140 | 12 | 1.80 | 1.78E-03 | 4.62E-02 |
| NM_000480 | adenosine monophosphate deaminase (isoform E) | Hs.83918 | 11p15 | AMPD3 | 2015 | 15 | 1.80 | 5.76E-04 | 2.42E-02 |
| BC001646 | Calcium binding protein P22 |  |  |  | 246 | 14 | 1.80 | 2.63E-03 | 5.55E-02 |
| NM_006679 | Putative opioid receptor, neuromedin K (neurokinin B) receptor-like |  |  |  | 5107 | 15 | 1.80 | 5.70E-04 | 2.43E-02 |
| NM_032712 | MGC13170 gene | Hs.256301 | 19q13.41 | MGC13170 | 1611 | 16 | 1.81 | 4.43E-03 | 7.14E-02 |
| AB014547 | myotubularin related protein 4 | Hs.141727 | 17q22-q23 | MTMR4 | 871 | 16 | 1.81 | 1.23E-03 | 3.79E-02 |
| NM_032306 | hypothetical protein MGC10974 | Hs.111099 | 19p13.3 | MGC10974 | 2719 | 15 | 1.81 | 7.54E-03 | 9.34E-02 |
| AB011151 | BDG-29 proten | Hs.81505 | 16q24.2 | BDG29 | 475 | 16 | 1.81 | 1.11E-03 | 3.58E-02 |
| AK024174 | Homo sapiens cDNA FLJ42818 fis, clone BRCAN2015371 | Hs.370214 | 8q13.1 | WAC | 700 | 13 | 1.81 | 4.95E-03 | 7.46E-02 |
| NM_006575 | mitogen-activated protein kinase kinase kinase kinase 5 | Hs.246970 | 14q11.2-q21 | MAP4K5 | 163 | 16 | 1.81 | 7.40E-03 | 9.27E-02 |
| AK055651 | Homo sapiens cDNA FLJ31089 fis, clone IMR321000092. | Hs.22216 | 15q21 | SCG3 | 647 | 16 | 1.81 | 3.97E-05 | 5.22E-03 |
| NM_001695 | ATPase, H+ transporting, lysosomal 42kDa, V1 subunit C, isoform 1 | Hs.86905 | 8q22.3 | ATP6V1C1 | 1064 | 15 | 1.83 | 3.88E-03 | 6.73E-02 |
| NM_002742 | protein kinase C, mu | Hs.2891 | 14q11 | PRKCM | 217 | 16 | 1.83 | 8.99E-04 | 3.19E-02 |
| AK024911 | Heterogeneous nuclear ribonucleoprotein M |  |  |  | 1860 | 16 | 1.83 | 1.38E-04 | 1.08E-02 |
| NM_014520 | MYB binding protein (P160) 1a | Hs.22824 | 17p13.3 | MYBBP1A | 4981 | 16 | 1.84 | 1.40E-04 | 1.09E-02 |
| NM_018355 | zinc finger protein 415 | Hs.21838 | 19q13.42 | ZNF415 | 428 | 14 | 1.84 | 4.73E-03 | 7.34E-02 |
| NM_014669 | KIAA0095 gene product | Hs.295014 | 16q13 | KIAA0095 | 1208 | 15 | 1.84 | 8.17E-03 | 9.66E-02 |
| NM_024616 | hypothetical protein FLJ23186 | Hs.434247 | 3q13.2 | FLJ23186 | 706 | 15 | 1.84 | 4.49E-03 | 7.18E-02 |
| NM_016656 | Ras-related GTP binding B | Hs.50282 | Xp11.22 | RRAGB | 325 | 11 | 1.85 | 6.09E-03 | 8.33E-02 |
| AL110197 | tissue inhibitor of metalloproteinase 2 | Hs.6441 | 17q25 | TIMP2 | 7349 | 16 | 1.85 | 1.46E-04 | 1.11E-02 |
| AK057307 | hypothetical protein FLJ32745 | Hs.437114 | 2q12.3 | FLJ32745 | 549 | 13 | 1.85 | 1.49E-03 | 4.21E-02 |
| NM_003446 | zinc finger protein 157 (HZF22) | Hs.89897 | Xp11.2 | ZNF157 | 575 | 12 | 1.85 | 7.23E-03 | 9.13E-02 |
| AK055323 | Homo sapiens cDNA FLJ30761 fis, clone FEBRA2000538. | Hs.19339 | 1p35.3 | FLJ10349 | 164 | 14 | 1.85 | 8.64E-04 | 3.11E-02 |
| NM_005195 | KIAA0146 protein | Hs.381058 | 8q11.21 | KIAA0146 | 1233 | 15 | 1.85 | 2.98E-03 | 5.92E-02 |
| BC010682 | hypothetical protein BC010682 | Hs.4896 | 10q22.2 | LOC90550 | 2438 | 14 | 1.85 | 4.82E-05 | 6.08E-03 |
| NM_005131 | THO complex 1 | Hs.1540 | 18p11.32 | THOC1 | 636 | 15 | 1.85 | 1.67E-04 | 1.19E-02 |
| NM_024050 | hypothetical protein MGC2594 | Hs.181551 | 19p13.12 | MGC2594 | 632 | 16 | 1.85 | 4.51E-03 | 7.20E-02 |
| NM_003481 | ubiquitin specific protease 5 (isopeptidase T) | Hs.3759 | 12p13 | USP5 | 1831 | 16 | 1.85 | 4.29E-04 | 2.05E-02 |
| BC012010 | hypothetical protein BC012010 | Hs.511769 | 11p15.1 | LOC113174 | 588 | 13 | 1.86 | 2.19E-03 | 5.08E-02 |
| AB058766 | KIAA1863 protein |  |  |  | 1540 | 15 | 1.86 | 2.55E-03 | 5.47E-02 |
| NM_016098 | brain protein 44-like | Hs.117963 | 6q27 | BRP44L | 292 | 16 | 1.86 | 4.87E-05 | 6.05E-03 |
| NM_024039 | homolog of yeast Mis12 | Hs.267194 | 17p13.3 | MIS12 | 428 | 14 | 1.86 | 1.06E-03 | 3.50E-02 |
| M96739 | nescient helix loop helix 1 | Hs.30956 | 1q22 | NHLH1 | 397 | 9 | 1.86 | 3.91E-03 | 6.74E-02 |
| AK055367 | chromosome 14 open reading frame 126 | Hs.170641 | 14q12 | C14orf126 | 296 | 12 | 1.86 | 6.83E-03 | 8.84E-02 |
| BC015706 | hypothetical protein MGC:10200 | Hs.134726 | 18q21.1 | MGC10200 | 7684 | 16 | 1.86 | 7.89E-05 | 7.75E-03 |
| AY050169 | hypothetical protein FLJ10081 | Hs.337625 | 2p12-p11.2 | FLJ10081 | 1451 | 13 | 1.86 | 3.37E-03 | 6.29E-02 |
| BC004409 | hypothetical protein BC004409 | Hs.449641 | 10q26.3 | LOC92170 | 826 | 16 | 1.86 | 2.01E-03 | 4.88E-02 |
| AB020671 | Rho interacting protein 3 | Hs.430725 | 17p11.2 | RHOIP3 | 5439 | 16 | 1.86 | 7.13E-05 | 7.39E-03 |
| AK057865 | Thy-1 cell surface antigen | Hs.134643 | 11q22.3-q23 | THY1 | 166 | 13 | 1.86 | 7.16E-03 | 9.09E-02 |
| NM_005415 | solute carrier family 20 (phosphate transporter), member 1 | Hs.110855 | 2q11-q14 | SLC20A1 | 8860 | 16 | 1.86 | 5.76E-04 | 2.42E-02 |
| NM_017977 | absent in melanoma 1-like | Hs.128738 | 1p35.3 | AIM1L | 262 | 16 | 1.87 | 1.26E-03 | 3.85E-02 |
| S72422 | dihydrolipoamide S-succinyltransferase (E2 component of 2-oxo-glutarate complex) | Hs.480230 | 14q24.3 | DLST | 731 | 14 | 1.87 | 3.46E-04 | 1.83E-02 |
| BC010642 | zinc finger protein 22 (KOX 15) | Hs.108642 | 10q11 | ZNF22 | 974 | 16 | 1.87 | 1.67E-03 | 4.44E-02 |
| NM_018983 | nucleolar protein family A, member 1 (H/ACA small nucleolar RNPs) | Hs.69851 | 4q25 | NOLA1 | 184 | 12 | 1.87 | 1.39E-03 | 4.09E-02 |
| NM_024407 | NADH dehydrogenase (ubiquinone) Fe-S protein 7 (20kD) (NADH-coenzyme Q reductase) |  |  |  | 1189 | 15 | 1.87 | 4.97E-03 | 7.47E-02 |
| NM_003961 | rhomboid, veinlet-like 1 (Drosophila) | Hs.137572 | 16p13.3 | RHBDL1 | 386 | 16 | 1.87 | 1.54E-04 | 1.13E-02 |
| BC013767 | hypothetical protein BC013767 | Hs.436584 | 16p13.13 | LOC114990 | 347 | 12 | 1.88 | 4.53E-03 | 7.21E-02 |
| NM_032193 | AYP1 protein | Hs.334800 | 11q13.1 | AYP1 | 814 | 16 | 1.88 | 1.91E-03 | 4.74E-02 |
| BC001648 | WD repeat domain 18 | Hs.325321 | 19p13.3 | WDR18 | 2051 | 15 | 1.88 | 1.86E-03 | 4.66E-02 |
| NM_024884 | chromosome 14 open reading frame 160 | Hs.512741 | 14q22.1 | C14orf160 | 491 | 14 | 1.88 | 2.46E-04 | 1.45E-02 |
| NM_006532 | elongation factor RNA polymerase II | Hs.5881 | 19p13.1 | ELL | 451 | 14 | 1.89 | 2.13E-03 | 5.01E-02 |
| AK057046 | hypothetical protein FLJ35155 | Hs.299823 | 3q29 | FLJ35155 | 235 | 12 | 1.89 | 4.80E-03 | 7.38E-02 |
| NM_030570 | uroplakin 3B | Hs.284211 | 7q11.2 | UPK3B | 785 | 14 | 1.89 | 1.56E-03 | 4.28E-02 |
| AL137723 | Homo sapiens mRNA; cDNA DKFZp434D0818 (from clone DKFZp434D0818) | Hs.5855 | 17q22-q23 | MRPS23 | 326 | 14 | 1.90 | 1.07E-03 | 3.50E-02 |
| NM_002778 | prosaposin (variant Gaucher disease and variant metachromatic leukodystrophy) | Hs.406455 | 10q21-q22 | PSAP | 941 | 16 | 1.90 | 1.31E-03 | 3.92E-02 |
| NM_003655 | chromobox homolog 4 (Pc class homolog, Drosophila) | Hs.5637 | 17q25.3 | CBX4 | 470 | 12 | 1.90 | 3.24E-04 | 1.76E-02 |
| NM_014762 | 24-dehydrocholesterol reductase | Hs.75616 | 1p33-p31.1 | DHCR24 | 18358 | 16 | 1.90 | 2.89E-03 | 5.82E-02 |
| NM_032358 | hypothetical protein MGC13183 | Hs.512781 | 12p13.33 | MGC13183 | 264 | 15 | 1.90 | 3.82E-03 | 6.67E-02 |
| NM_032014 | mitochondrial ribosomal protein S24 | Hs.284286 | 7p14 | MRPS24 | 2049 | 16 | 1.90 | 2.57E-03 | 5.49E-02 |
| NM_003682 | MAP-kinase activating death domain | Hs.82548 | 11p11.2 | MADD | 402 | 15 | 1.90 | 3.66E-03 | 6.54E-02 |
| NM_003297 | nuclear receptor subfamily 2, group C, member 1 | Hs.108301 | 12q23.1 | NR2C1 | 570 | 13 | 1.91 | 1.79E-03 | 4.64E-02 |
| NM_032632 | poly(A) polymerase alpha | Hs.201085 | 14q32.31 | PAPOLA | 471 | 16 | 1.91 | 3.31E-04 | 1.76E-02 |
| AK055002 | Homo sapiens cDNA FLJ30440 fis, clone BRACE2009185. | Hs.421612 | 2q33-q34 | EEF1B2 | 145 | 6 | 1.91 | 3.76E-03 | 6.62E-02 |
| NM_005560 | laminin, alpha 5 | Hs.11669 | 20q13.2-q13.3 | LAMA5 | 2390 | 15 | 1.91 | 6.67E-04 | 2.65E-02 |
| NM_022839 | mitochondrial ribosomal protein S11 | Hs.111286 | 15q25 | MRPS11 | 320 | 14 | 1.91 | 3.12E-03 | 6.11E-02 |
| BC001619 | aldehyde dehydrogenase 1 family, member B1 | Hs.436219 | 9p11.1 | ALDH1B1 | 724 | 12 | 1.91 | 6.79E-03 | 8.82E-02 |
| NM_014583 | LIM and cysteine-rich domains 1 | Hs.279943 | 3p26-p24 | LMCD1 | 464 | 16 | 1.91 | 3.31E-03 | 6.23E-02 |
| NM_003332 | TYRO protein tyrosine kinase binding protein | Hs.9963 | 19q13.1 | TYROBP | 462 | 16 | 1.92 | 2.26E-04 | 1.38E-02 |
| BC011751 | spermidine/spermine N1-acetyltransferase 2 | Hs.10846 | 17p13.2 | SAT2 | 637 | 12 | 1.92 | 8.25E-04 | 3.06E-02 |
| BC015138 | 5'-nucleotidase, cytosolic II-like 1 | Hs.511931 | 6q22.31 | NT5C2L1 | 160 | 11 | 1.92 | 2.47E-03 | 5.39E-02 |
| BC014989 | Phospholipid scramblase 3 |  |  |  | 3121 | 15 | 1.92 | 1.60E-04 | 1.14E-02 |
| NM_017815 | chromosome 14 open reading frame 94 | Hs.442782 | 14q11.2 | C14orf94 | 401 | 14 | 1.92 | 6.77E-05 | 7.30E-03 |
| AL137733 | Homo sapiens mRNA; cDNA DKFZp434M0420 (from clone DKFZp434M0420) | Hs.406264 | 17q24.2 | LOC284021 | 466 | 16 | 1.93 | 8.86E-04 | 3.15E-02 |
| NM_014698 | KIAA0792 gene product | Hs.119387 | 1q42.13 | KIAA0792 | 326 | 14 | 1.93 | 2.45E-03 | 5.36E-02 |
| AK057419 | hypothetical protein LOC285033 | Hs.58648 | 2q11.2 | LOC285033 | 264 | 14 | 1.93 | 6.15E-04 | 2.52E-02 |
| NM_020232 | hepatocellular carcinoma susceptibility protein | Hs.386802 | 18p11.21 | HCCA3 | 3075 | 15 | 1.93 | 9.41E-05 | 8.54E-03 |
| NM_001005 | ribosomal protein S3 | Hs.387576 | 11q13.3-q13.5 | RPS3 | 3895 | 16 | 1.93 | 3.96E-03 | 6.79E-02 |
| NM_004450 | enhancer of rudimentary homolog (Drosophila) | Hs.433413 | 14q24.1 | ERH | 4562 | 16 | 1.94 | 6.07E-03 | 8.32E-02 |
| NM_005139 | annexin A3 | Hs.442733 | 4q13-q22 | ANXA3 | 3884 | 16 | 1.94 | 2.26E-03 | 5.19E-02 |
| NM_001786 | cell division cycle 2, G1 to S and G2 to M | Hs.334562 | 10q21.1 | CDC2 | 2562 | 16 | 1.94 | 1.08E-03 | 3.53E-02 |
| NM_007039 | protein tyrosine phosphatase, non-receptor type 21 | Hs.437040 | 14q31.3 | PTPN21 | 182 | 14 | 1.95 | 4.24E-03 | 7.02E-02 |
| AK055071 | phosphatidylinositol glycan, class K | Hs.293653 | 1p31.1 | PIGK | 77 | 8 | 1.95 | 8.90E-03 | 9.97E-02 |
| AK054843 | hypothetical protein LOC201191 | Hs.320522 | 17q21.33 | LOC201191 | 206 | 13 | 1.95 | 4.75E-03 | 7.35E-02 |
| NM_018192 | myxoid liposarcoma associated protein 4 | Hs.42824 | 3q29 | MLAT4 | 6926 | 14 | 1.95 | 2.23E-04 | 1.38E-02 |
| BC006132 | pygopus 2 | Hs.172084 | 1q22 | PYGO2 | 415 | 14 | 1.95 | 1.83E-03 | 4.66E-02 |
| AL109703 | hypothetical protein LOC283761 | Hs.25314 | 15q25.3 | LOC283761 | 533 | 14 | 1.95 | 2.69E-03 | 5.63E-02 |
| NM_018127 | elaC homolog 2 (E. coli) | Hs.12124 | 17p11.2 | ELAC2 | 2373 | 15 | 1.95 | 1.88E-04 | 1.26E-02 |
| NM_022117 | cutaneous T-cell lymphoma-associated tumor antigen se20-4 | Hs.136164 | Xp11.2 | SE20-4 | 223 | 12 | 1.97 | 8.68E-03 | 9.87E-02 |
| AK027620 | PHD protein Jade-1 | Hs.12420 | 4q26-q27 | Jade-1 | 115 | 13 | 1.97 | 4.47E-03 | 7.16E-02 |
| AK022842 | hypothetical protein MGC42530 | Hs.21902 | 3q13.31 | MGC42530 | 386 | 14 | 1.98 | 2.03E-04 | 1.30E-02 |
| NM_012124 | cysteine and histidine-rich domain (CHORD)-containing, zinc binding protein 1 | Hs.22857 | 11q14.3 | CHORDC1 | 2938 | 15 | 1.98 | 1.80E-03 | 4.63E-02 |
| AK055335 | Homo sapiens cDNA FLJ30773 fis, clone FEBRA2000771. | Hs.75322 | 2q36.1 | TUBA1 | 218 | 7 | 1.98 | 6.21E-03 | 8.43E-02 |
| NM_004433 | E74-like factor 3 (ets domain transcription factor, epithelial-specific ) | Hs.67928 | 1q32.2 | ELF3 | 662 | 16 | 1.98 | 6.23E-03 | 8.43E-02 |
| NM_000159 | glutaryl-Coenzyme A dehydrogenase | Hs.292233 | 19p13.2 | GCDH | 353 | 11 | 1.98 | 6.59E-04 | 2.63E-02 |
| NM_002824 | parathymosin | Hs.446525 | 12p13 | PTMS | 3144 | 16 | 1.98 | 3.55E-04 | 1.84E-02 |
| NM_025138 | hypothetical protein FLJ12661 | Hs.318526 | 13q13.3 | FLJ12661 | 148 | 10 | 1.98 | 6.62E-03 | 8.72E-02 |
| NM_001513 | glutathione transferase zeta 1 (maleylacetoacetate isomerase) | Hs.26403 | 14q24.3 | GSTZ1 | 397 | 16 | 1.98 | 5.02E-05 | 6.14E-03 |
| NM_022164 | lipocalin 7 | Hs.173508 | 1p35.1 | LCN7 | 781 | 15 | 1.98 | 4.08E-03 | 6.85E-02 |
| NM_025268 | hole gene | Hs.157527 | 14q32.33 | MGC4659 | 242 | 13 | 1.98 | 4.37E-03 | 7.11E-02 |
| NM_001383 | Diptheria toxin resistance protein required for diphthamide biosynthesis-like 1 (S. cerevisiae) |  |  |  | 390 | 15 | 1.99 | 1.65E-03 | 4.41E-02 |
| NM_014584 | ERO1-like (S. cerevisiae) | Hs.10949 | 14q22.1 | ERO1L | 779 | 13 | 1.99 | 8.44E-04 | 3.09E-02 |
| NM_006427 | CD27-binding (Siva) protein | Hs.112058 | 14q32.33 | SIVA | 2183 | 16 | 1.99 | 1.26E-03 | 3.84E-02 |
| NM_005134 | protein phosphatase 4, regulatory subunit 1 | Hs.435789 | 18p11.22 | PPP4R1 | 1964 | 16 | 1.99 | 1.17E-04 | 9.75E-03 |
| NM_032241 | Ribosomal protein L10 |  |  |  | 371 | 15 | 2.00 | 8.81E-03 | 9.96E-02 |
| AL110257 | hypothetical protein MGC26690 | Hs.161145 | 15q21.3 | MGC26690 | 202 | 13 | 2.00 | 6.37E-03 | 8.49E-02 |
| NM_007202 | A kinase (PRKA) anchor protein 10 | Hs.372446 | 17p11.1 | AKAP10 | 281 | 16 | 2.00 | 7.32E-03 | 9.20E-02 |
| NM_006084 | interferon-stimulated transcription factor 3, gamma 48kDa | Hs.1706 | 14q11.2 | ISGF3G | 261 | 14 | 2.00 | 2.46E-04 | 1.45E-02 |
| NM_018465 | uncharacterized hematopoietic stem/progenitor cells protein MDS030 | Hs.416649 | 9p24.1 | MDS030 | 529 | 11 | 2.00 | 1.10E-03 | 3.57E-02 |
| NM_014373 | putative G protein-coupled receptor | Hs.231320 | 3q26.2-q27 | GPCR1 | 1055 | 14 | 2.00 | 3.81E-04 | 1.87E-02 |
| NM_025246 | hypothetical protein MGC3295 | Hs.101257 | 3q22.3 | MGC3295 | 372 | 14 | 2.00 | 4.39E-03 | 7.11E-02 |
| NM_005710 | polyglutamine binding protein 1 | Hs.30570 | Xp11.23 | PQBP1 | 1227 | 13 | 2.01 | 1.34E-04 | 1.07E-02 |
| NM_003105 | sortilin-related receptor, L(DLR class) A repeats-containing | Hs.438159 | 11q23.2-q24.2 | SORL1 | 110 | 10 | 2.01 | 8.79E-04 | 3.14E-02 |
| NM_004275 | ubiquitin specific protease 49 | Hs.278434 | 6p21.1 | USP49 | 258 | 14 | 2.01 | 4.21E-03 | 7.01E-02 |
| BC014984 | small nuclear RNA activating complex, polypeptide 1, 43kDa | Hs.179312 | 14q22 | SNAPC1 | 427 | 14 | 2.01 | 3.75E-03 | 6.62E-02 |
| AF116653 | FYN binding protein (FYB-120/130) | Hs.276506 | 5p13.1 | FYB | 202 | 15 | 2.01 | 8.22E-05 | 7.93E-03 |
| NM_004240 | thyroid hormone receptor interactor 10 | Hs.445226 | 19p13.3 | TRIP10 | 896 | 10 | 2.01 | 7.83E-03 | 9.48E-02 |
| BC012077 | ring finger protein 31 | Hs.375217 | 14q11.2 | RNF31 | 403 | 8 | 2.01 | 5.03E-03 | 7.50E-02 |
| NM_003287 | tumor protein D52-like 1 | Hs.16611 | 6q22-q23 | TPD52L1 | 874 | 16 | 2.01 | 1.26E-04 | 1.03E-02 |
| NM_001919 | dodecenoyl-Coenzyme A delta isomerase (3,2 trans-enoyl-Coenzyme A isomerase) | Hs.403436 | 16p13.3 | DCI | 1137 | 16 | 2.02 | 5.32E-04 | 2.32E-02 |
| NM_052811 | ret finger protein 2 | Hs.436922 | 13q14 | RFP2 | 169 | 14 | 2.02 | 5.43E-03 | 7.84E-02 |
| NM_032813 | hypothetical protein FLJ14624 | Hs.172963 | 13q32.3 | FLJ14624 | 149 | 10 | 2.02 | 3.45E-03 | 6.37E-02 |
| NM_002431 | menage a trois 1 (CAK assembly factor) | Hs.72870 | 14q23 | MNAT1 | 866 | 14 | 2.02 | 1.34E-03 | 3.99E-02 |
| NM_016106 | chromosome 14 open reading frame 163 | Hs.27023 | 14q12 | C14orf163 | 713 | 13 | 2.02 | 2.98E-03 | 5.93E-02 |
| NM_005231 | ems1 sequence (mammary tumor and squamous cell carcinoma-associated (p80/85 src substrate) | Hs.301348 | 11q13 | EMS1 | 2371 | 16 | 2.02 | 8.18E-05 | 7.94E-03 |
| NM_012110 | cysteine-rich hydrophobic domain 2 | Hs.259209 | 4q11 | CHIC2 | 3762 | 14 | 2.02 | 4.80E-03 | 7.38E-02 |
| NM_024294 | hypothetical protein MGC4614 | Hs.300691 | 6p21.31 | MGC4614 | 174 | 13 | 2.02 | 6.04E-03 | 8.31E-02 |
| NM_016445 | pleckstrin 2 | Hs.170473 | 14q24.1 | PLEK2 | 1052 | 16 | 2.03 | 1.37E-04 | 1.08E-02 |
| M60502 | filaggrin | Hs.73995 | 1q21 | FLG | 1550 | 16 | 2.03 | 2.50E-03 | 5.41E-02 |
| NM_017784 | oxysterol binding protein-like 10 | Hs.368238 | 3p22.3 | OSBPL10 | 408 | 15 | 2.04 | 5.81E-04 | 2.43E-02 |
| NM_001977 | glutamyl aminopeptidase (aminopeptidase A) | Hs.435765 | 4q25 | ENPEP | 270 | 15 | 2.04 | 2.85E-03 | 5.80E-02 |
| X69699 | paired box gene 8 | Hs.308061 | 2q12-q14 | PAX8 | 809 | 15 | 2.04 | 1.34E-03 | 3.99E-02 |
| NM_000687 | S-adenosylhomocysteine hydrolase | Hs.388004 | 20cen-q13.1 | AHCY | 1465 | 15 | 2.04 | 1.10E-03 | 3.57E-02 |
| NM_018224 | hypothetical protein FLJ10803 | Hs.289007 | 7p13 | FLJ10803 | 175 | 11 | 2.04 | 3.30E-03 | 6.22E-02 |
| NM_003258 | thymidine kinase 1, soluble | Hs.164457 | 17q23.2-q25.3 | TK1 | 1968 | 16 | 2.04 | 2.50E-04 | 1.46E-02 |
| NM_000724 | calcium channel, voltage-dependent, beta 2 subunit | Hs.435786 | 10p12 | CACNB2 | 108 | 6 | 2.04 | 5.87E-03 | 8.16E-02 |
| NM_032731 | hypothetical protein MGC14353 | Hs.408236 | 17p13.2 | MGC14353 | 11437 | 16 | 2.04 | 7.77E-06 | 1.86E-03 |
| NM_006101 | highly expressed in cancer, rich in leucine heptad repeats | Hs.414407 | 18p11.31 | HEC | 3233 | 16 | 2.05 | 5.75E-05 | 6.71E-03 |
| NM_003819 | poly(A) binding protein, cytoplasmic 4 (inducible form) | Hs.169900 | 1p32-p36 | PABPC4 | 1442 | 15 | 2.05 | 6.26E-03 | 8.42E-02 |
| NM_002988 | chemokine (C-C motif) ligand 18 (pulmonary and activation-regulated) | Hs.16530 | 17q11.2 | CCL18 | 99 | 10 | 2.05 | 1.19E-03 | 3.75E-02 |
| NM_033546 | myosin regulatory light chain MRLC2 | Hs.511744 | 18p11.31 | MRLC2 | 3940 | 15 | 2.06 | 2.48E-03 | 5.40E-02 |
| NM_016619 | placenta-specific 8 | Hs.371003 | 4q21.3 | PLAC8 | 470 | 12 | 2.06 | 7.93E-03 | 9.57E-02 |
| AK055976 | thymosin, beta 4, X-linked | Hs.75968 | Xq21.3-q22 | TMSB4X | 15801 | 15 | 2.07 | 1.95E-03 | 4.80E-02 |
| Z85986 | Human DNA sequence from clone 108K11 on chromosome 6p21 Contains SRP20 (SR protein family member), N |  |  |  | 170 | 14 | 2.08 | 2.43E-03 | 5.34E-02 |
| AB007859 | KIAA0399 protein | Hs.172179 | 17p13.3 | KIAA0399 | 743 | 15 | 2.08 | 3.49E-04 | 1.83E-02 |
| NM_016039 | chromosome 14 open reading frame 166 | Hs.369840 | 14q22.1 | C14orf166 | 3894 | 15 | 2.08 | 1.26E-03 | 3.84E-02 |
| AK057575 | solute carrier family 25 (mitochondrial carrier; phosphate carrier), member 3 | Hs.290404 | 12q23 | SLC25A3 | 4453 | 14 | 2.08 | 1.77E-03 | 4.62E-02 |
| NM_023930 | hypothetical protein MGC2376 | Hs.17296 | 11q13.4 | MGC2376 | 252 | 15 | 2.09 | 3.25E-04 | 1.76E-02 |
| NM_000600 | interleukin 6 (interferon, beta 2) | Hs.512234 | 7p21 | IL6 | 468 | 16 | 2.09 | 2.83E-03 | 5.80E-02 |
| NM_031216 | sec13-like protein | Hs.301048 | 18p11.21 | SEC13L | 709 | 16 | 2.09 | 3.30E-03 | 6.22E-02 |
| NM_016213 | thyroid hormone receptor interactor 4 | Hs.116784 | 15q22.1 | TRIP4 | 239 | 14 | 2.09 | 3.25E-03 | 6.22E-02 |
| NM_000121 | erythropoietin receptor | Hs.127826 | 19p13.3-p13.2 | EPOR | 672 | 14 | 2.09 | 9.50E-04 | 3.27E-02 |
| NM_004140 | lethal giant larvae homolog 1 (Drosophila) | Hs.95659 | 17p11.2 | LLGL1 | 373 | 8 | 2.09 | 4.99E-03 | 7.48E-02 |
| AB028962 | KIAA1039 protein | Hs.499659 | 17p13.3 | KIAA1039 | 701 | 16 | 2.10 | 5.44E-05 | 6.49E-03 |
| AK021425 | hypothetical protein DJ667H12.2 | Hs.445835 | 1q32.1-q41 | DJ667H12.2 | 96 | 12 | 2.10 | 4.05E-03 | 6.85E-02 |
| NM_016835 | microtubule-associated protein tau | Hs.101174 | 17q21.1 | MAPT | 216 | 14 | 2.10 | 4.78E-04 | 2.16E-02 |
| NM_006821 | peroxisomal long-chain acyl-coA thioesterase | Hs.446685 | 14q24.3 | ZAP128 | 282 | 15 | 2.10 | 7.14E-05 | 7.34E-03 |
| NM_023940 | hypothetical protein MGC2827 | Hs.8035 | 4q12 | MGC2827 | 365 | 16 | 2.11 | 2.10E-03 | 4.97E-02 |
| NM_005776 | cornichon homolog (Drosophila) | Hs.294603 | 14q22.2 | CNIH | 420 | 10 | 2.12 | 7.09E-03 | 9.04E-02 |
| BC009732 | hypothetical protein BC009732 | Hs.445192 | 4q24 | LOC133308 | 140 | 8 | 2.12 | 5.67E-04 | 2.43E-02 |
| BC014072 | hypothetical protein BC014072 | Hs.348504 | 17q11.2 | LOC116238 | 158 | 11 | 2.13 | 3.72E-03 | 6.58E-02 |
| NM_021210 | trafficking protein particle complex 1 | Hs.24379 | 17p13.2 | TRAPPC1 | 1899 | 16 | 2.13 | 1.86E-04 | 1.26E-02 |
| AK056212 | Homo sapiens cDNA FLJ31678 fis, clone NT2RI2005130. | Hs.525322 | 12p13.1 | na | 218 | 5 | 2.13 | 2.84E-03 | 5.81E-02 |
| BC014280 | zinc finger protein 71 (Cos26) | Hs.301431 | 19q13.4 | ZNF71 | 266 | 6 | 2.13 | 7.13E-03 | 9.06E-02 |
| AL390163 | Homo sapiens hypothetical gene supported by AL390163; NM_153030 (LOC347803), mRNA | Hs.138430 | Xq13.1 | LOC158863 | 130 | 12 | 2.14 | 1.12E-03 | 3.60E-02 |
| NM_006445 | PRP8 pre-mRNA processing factor 8 homolog (yeast) | Hs.181368 | 17p13.3 | PRPF8 | 1424 | 15 | 2.14 | 4.59E-04 | 2.12E-02 |
| NM_005766 | FERM, RhoGEF (ARHGEF) and pleckstrin domain protein 1 (chondrocyte-derived) | Hs.207428 | 13q32.2-q32.3 | FARP1 | 1498 | 16 | 2.14 | 2.91E-04 | 1.64E-02 |
| AK000757 | sortilin 1 | Hs.394609 | 1p21.3-p13.1 | SORT1 | 318 | 14 | 2.14 | 3.27E-03 | 6.22E-02 |
| NM_001380 | dedicator of cyto-kinesis 1 | Hs.437620 | 10q26.13-q26.3 | DOCK1 | 602 | 12 | 2.15 | 2.10E-03 | 4.97E-02 |
| NM_000177 | gelsolin (amyloidosis, Finnish type) | Hs.446537 | 9q33 | GSN | 206 | 12 | 2.15 | 6.54E-04 | 2.65E-02 |
| NM_012062 | dynamin 1-like | Hs.180628 | 12p11.21 | DNM1L | 1211 | 14 | 2.15 | 3.79E-04 | 1.88E-02 |
| NM_002929 | rhodopsin kinase | Hs.103501 | 13q34 | RHOK | 446 | 10 | 2.15 | 3.26E-03 | 6.23E-02 |
| NM_032717 | hypothetical protein MGC11324 | Hs.99196 | 4q21.3 | MGC11324 | 321 | 14 | 2.15 | 7.32E-05 | 7.43E-03 |
| AB028986 | ubiquitin specific protease 22 | Hs.12064 | 17p11.2 | USP22 | 3772 | 16 | 2.16 | 9.47E-06 | 2.10E-03 |
| NM_014646 | lipin 2 | Hs.437425 | 18p11.31 | LPIN2 | 416 | 14 | 2.16 | 5.90E-04 | 2.46E-02 |
| NM_024551 | adiponectin receptor 2 | Hs.334854 | 12p13.31 | ADIPOR2 | 638 | 13 | 2.16 | 2.25E-03 | 5.18E-02 |
| NM_000036 | adenosine monophosphate deaminase 1 (isoform M) | Hs.89570 | 1p13 | AMPD1 | 156 | 13 | 2.16 | 2.81E-03 | 5.79E-02 |
| BC007429 | syntaxin 3A | Hs.82240 | 11q12.2 | STX3A | 365 | 11 | 2.16 | 6.99E-03 | 8.97E-02 |
| NM_006587 | corin | Hs.340634 | 4p13-p12 | CRN | 352 | 15 | 2.17 | 7.32E-04 | 2.85E-02 |
| NM_001400 | endothelial differentiation, sphingolipid G-protein-coupled receptor, 1 | Hs.154210 | 1p21 | EDG1 | 408 | 16 | 2.17 | 1.23E-04 | 1.01E-02 |
| NM_007169 | phosphatidylethanolamine N-methyltransferase | Hs.15192 | 17p11.2 | PEMT | 623 | 13 | 2.17 | 7.45E-04 | 2.89E-02 |
| NM_006029 | paraneoplastic antigen MA1 | Hs.194709 | 14q24.2 | PNMA1 | 972 | 15 | 2.17 | 1.92E-03 | 4.74E-02 |
| NM_003929 | RAB7, member RAS oncogene family-like 1 | Hs.115325 | 1q32 | RAB7L1 | 840 | 14 | 2.17 | 3.57E-04 | 1.84E-02 |
| NM_000923 | phosphodiesterase 4C, cAMP-specific (phosphodiesterase E1 dunce homolog, Drosophila) | Hs.437211 | 19p13.11 | PDE4C | 215 | 13 | 2.18 | 2.60E-03 | 5.51E-02 |
| NM_006197 | pericentriolar material 1 | Hs.348501 | 8p22-p21.3 | PCM1 | 9773 | 6 | 2.18 | 5.92E-04 | 2.46E-02 |
| Z24725 | pleckstrin homology domain containing, family C (with FERM domain) member 1 | Hs.270411 | 14q22.1 | PLEKHC1 | 3803 | 16 | 2.18 | 6.20E-05 | 6.93E-03 |
| NM_032856 | hypothetical protein FLJ14888 | Hs.29863 | 15q25.2 | FLJ14888 | 486 | 11 | 2.18 | 5.84E-03 | 8.15E-02 |
| NM_019067 | hypothetical protein FLJ10613 | Hs.438583 | Xp11.22 | FLJ10613 | 189 | 14 | 2.18 | 2.29E-03 | 5.18E-02 |
| NM_005619 | reticulon 2 | Hs.47517 | 19q13.32 | RTN2 | 526 | 16 | 2.18 | 5.89E-05 | 6.77E-03 |
| NM_020982 | claudin 9 | Hs.296949 | 16p13.3 | CLDN9 | 544 | 15 | 2.18 | 7.43E-04 | 2.89E-02 |
| NM_004364 | CCAAT/enhancer binding protein (C/EBP), alpha | Hs.76171 | 19q13.1 | CEBPA | 721 | 12 | 2.19 | 4.02E-03 | 6.84E-02 |
| AK025078 | Homo sapiens MSTP157 (MST157) mRNA, complete cds | Hs.302738 | 16p12 | XYLT1 | 291 | 16 | 2.19 | 5.95E-03 | 8.23E-02 |
| BC007758 | hypothetical protein FKSG28 | Hs.512762 | 10q24.32 | FKSG28 | 183 | 11 | 2.19 | 3.76E-03 | 6.61E-02 |
| AK057715 | myosin regulatory light chain MRCL3 | Hs.233936 | 18p11.31 | MRCL3 | 249 | 12 | 2.20 | 1.06E-03 | 3.50E-02 |
| NM_001844 | collagen, type II, alpha 1 (primary osteoarthritis, spondyloepiphyseal dysplasia, congenital) | Hs.408182 | 12q13.11-q13.2 | COL2A1 | 119 | 9 | 2.20 | 8.57E-03 | 9.85E-02 |
| AK024763 | small nuclear RNA activating complex, polypeptide 5, 19kDa | Hs.30174 | 15q22.2 | SNAPC5 | 232 | 12 | 2.21 | 2.09E-03 | 4.97E-02 |
| NM_022126 | phospholysine phosphohistidine inorganic pyrophosphate phosphatase | Hs.20950 | 10q26.2 | LHPP | 332 | 16 | 2.21 | 6.27E-05 | 6.90E-03 |
| NM_016147 | protein phosphatase methylesterase-1 | Hs.63304 | 11q13.3 | PME-1 | 1709 | 15 | 2.21 | 8.13E-05 | 7.94E-03 |
| NM_003702 | regulator of G-protein signalling 20 | Hs.141492 | 8q12.1 | RGS20 | 583 | 15 | 2.21 | 1.02E-03 | 3.44E-02 |
| NM_017622 | hypothetical protein FLJ20014 | Hs.129563 | 17p13.1 | FLJ20014 | 202 | 14 | 2.21 | 6.58E-04 | 2.63E-02 |
| NM_000304 | peripheral myelin protein 22 | Hs.372031 | 17p12-p11.2 | PMP22 | 666 | 16 | 2.21 | 3.76E-04 | 1.87E-02 |
| AK022936 | Homo sapiens cDNA FLJ12874 fis, clone NT2RP2003769. | Hs.56847 | 10p15 | GDI2 | 515 | 15 | 2.22 | 4.77E-04 | 2.17E-02 |
| AB051530 | DAB2 interacting protein | Hs.238465 | 6q25.1 | DAB2IP | 207 | 14 | 2.22 | 4.52E-03 | 7.20E-02 |
| NM_018995 | Mov10l1, Moloney leukemia virus 10-like 1, homolog (mouse) | Hs.62880 | 22q13.33 | MOV10L1 | 120 | 5 | 2.23 | 8.41E-03 | 9.74E-02 |
| AY052405 | peptidylprolyl isomerase (cyclophilin) like 5 | Hs.451090 | 14q21.3 | PPIL5 | 325 | 14 | 2.23 | 2.60E-03 | 5.52E-02 |
| NM_022751 | hypothetical protein FLJ21610 | Hs.12727 | 18q12.1 | FLJ21610 | 76 | 7 | 2.23 | 5.86E-03 | 8.16E-02 |
| NM_014632 | flavoprotein oxidoreductase MICAL2 | Hs.309674 | 11p15.3 | MICAL2 | 108 | 5 | 2.24 | 8.72E-03 | 9.88E-02 |
| NM_001628 | aldo-keto reductase family 1, member B1 (aldose reductase) | Hs.75313 | 7q35 | AKR1B1 | 625 | 14 | 2.24 | 5.12E-03 | 7.58E-02 |
| NM_016531 | Kruppel-like factor 3 (basic) | Hs.145754 | 4p14 | KLF3 | 1391 | 14 | 2.25 | 8.89E-04 | 3.16E-02 |
| AK026565 | hypothetical protein FLJ10534 | Hs.388170 | 17p13.3 | FLJ10534 | 440 | 14 | 2.25 | 3.60E-03 | 6.47E-02 |
| NM_004346 | caspase 3, apoptosis-related cysteine protease | Hs.141125 | 4q34 | CASP3 | 870 | 13 | 2.25 | 1.57E-04 | 1.13E-02 |
| NM_003255 | tissue inhibitor of metalloproteinase 2 | Hs.6441 | 17q25 | TIMP2 | 1776 | 15 | 2.26 | 1.11E-03 | 3.59E-02 |
| NM_019013 | hypothetical protein FLJ10156 | Hs.404323 | 17p13.2 | FLJ10156 | 762 | 16 | 2.26 | 1.71E-04 | 1.20E-02 |
| NM_022140 | erythrocyte membrane protein band 4.1 like 4A | Hs.104746 | 5q22.2 | EPB41L4A | 95 | 12 | 2.27 | 3.21E-04 | 1.77E-02 |
| BC006441 | similar to RNA polymerase I transcription factor RRN3 | Hs.348979 | 16p12.3 | LOC94431 | 195 | 11 | 2.28 | 6.52E-03 | 8.62E-02 |
| AK058138 | hypothetical protein FLJ25409 | Hs.289399 | 8p11.22 | FLJ25409 | 302 | 12 | 2.28 | 7.13E-03 | 9.07E-02 |
| NM_001562 | interleukin 18 (interferon-gamma-inducing factor) | Hs.83077 | 11q22.2-q22.3 | IL18 | 802 | 14 | 2.29 | 7.88E-04 | 2.98E-02 |
| NM_024897 | hypothetical protein FLJ22672 | Hs.235873 | 1q23.1 | FLJ22672 | 119 | 11 | 2.29 | 3.98E-03 | 6.80E-02 |
| NM_000937 | polymerase (RNA) II (DNA directed) polypeptide A, 220kDa | Hs.171880 | 17p13.1 | POLR2A | 4461 | 15 | 2.29 | 9.25E-05 | 8.45E-03 |
| AK026977 | myosin, heavy polypeptide 10, non-muscle | Hs.280311 | 17p13 | MYH10 | 1962 | 16 | 2.29 | 1.44E-03 | 4.16E-02 |
| AK024946 | Homo sapiens cDNA: FLJ21293 fis, clone COL01972 | Hs.257043 | 19q13.2 | PLD3 | 678 | 14 | 2.30 | 5.26E-04 | 2.31E-02 |
| AK055655 | Homo sapiens cDNA FLJ31093 fis, clone IMR321000161. | Hs.32347 | 2q32 | INPP1 | 94 | 9 | 2.31 | 1.31E-03 | 3.92E-02 |
| NM_002284 | keratin, hair, basic, 6 (monilethrix) | Hs.278658 | 12q13 | KRTHB6 | 74 | 5 | 2.31 | 1.53E-03 | 4.27E-02 |
| NM_018845 | stromal cell protein | Hs.292154 | 1q22 | LOC55974 | 235 | 16 | 2.31 | 2.10E-04 | 1.32E-02 |
| AK024506 | chromosome 14 open reading frame 80 | Hs.72363 | 14q32.33 | C14orf80 | 1407 | 15 | 2.31 | 2.26E-04 | 1.38E-02 |
| BF698884 | DC2 protein | Hs.103180 | 4q25 | DC2 | 906 | 8 | 2.31 | 2.21E-03 | 5.10E-02 |
| AK002042 | hypothetical protein LOC221300 | Hs.134795 | 6q22.31 | LOC221300 | 1794 | 13 | 2.31 | 8.64E-06 | 2.00E-03 |
| BC001356 | interferon-induced protein 35 | Hs.50842 | 17q21 | IFI35 | 390 | 15 | 2.33 | 1.96E-04 | 1.28E-02 |
| NM_020979 | adaptor protein with pleckstrin homology and src homology 2 domains | Hs.371366 | 7q22 | APS | 215 | 13 | 2.34 | 3.44E-03 | 6.36E-02 |
| NM_015654 | DKFZP564C103 protein | Hs.144058 | 17q25.2 | DKFZP564C103 | 1207 | 16 | 2.35 | 1.46E-03 | 4.19E-02 |
| NM_003295 | tumor protein, translationally-controlled 1 | Hs.374596 | 13q12-q14 | TPT1 | 3269 | 16 | 2.35 | 1.86E-03 | 4.66E-02 |
| NM_057161 | kelch domain containing 3 | Hs.412468 | 6p21.1 | KLHDC3 | 765 | 16 | 2.35 | 2.83E-05 | 4.18E-03 |
| NM_014964 | epsin 2 | Hs.7407 | 17p11.2 | EPN2 | 264 | 15 | 2.35 | 3.66E-04 | 1.85E-02 |
| AK024475 | DKFZP434I216 protein | Hs.188781 | 16q22.1 | DKFZP434I216 | 723 | 13 | 2.36 | 2.58E-04 | 1.49E-02 |
| X98507 | myosin IC | Hs.286226 | 17p13 | MYO1C | 807 | 16 | 2.36 | 9.69E-07 | 5.38E-04 |
| AK025201 | nuclear transcription factor Y, alpha | Hs.10441 | 6p21.3 | NFYA | 345 | 14 | 2.37 | 1.64E-03 | 4.41E-02 |
| AB051555 | KIAA1768 protein | Hs.439680 | 10q26.3 | KIAA1768 | 390 | 14 | 2.37 | 1.66E-04 | 1.18E-02 |
| NM_007183 | plakophilin 3 | Hs.26557 | 11p15 | PKP3 | 338 | 15 | 2.37 | 1.58E-05 | 2.85E-03 |
| NM_000199 | N-sulfoglucosamine sulfohydrolase (sulfamidase) | Hs.31074 | 17q25.3 | SGSH | 282 | 8 | 2.38 | 3.67E-03 | 6.53E-02 |
| NM_004472 | forkhead box D1 | Hs.96028 | 5q12-q13 | FOXD1 | 585 | 15 | 2.39 | 1.28E-03 | 3.87E-02 |
| NM_020666 | CDC-like kinase 4 | Hs.406557 | 5q35 | CLK4 | 536 | 14 | 2.39 | 4.94E-04 | 2.22E-02 |
| NM_006796 | AFG3 ATPase family gene 3-like 2 (yeast) | Hs.436683 | 18p11 | AFG3L2 | 577 | 14 | 2.39 | 2.74E-03 | 5.68E-02 |
| NM_000299 | plakophilin 1 (ectodermal dysplasia/skin fragility syndrome) | Hs.313068 | 1q32 | PKP1 | 90 | 7 | 2.40 | 6.51E-03 | 8.61E-02 |
| BC016048 | hypothetical protein LOC126755 | Hs.263561 | 1p36.21 | LOC126755 | 128 | 7 | 2.40 | 8.00E-03 | 9.61E-02 |
| NM_001918 | dihydrolipoamide branched chain transacylase (E2 component of branched chain keto acid dehydrogenase complex; maple syrup urine disease) | Hs.139410 | 1p31 | DBT | 182 | 11 | 2.40 | 4.49E-03 | 7.18E-02 |
| NM_016432 | Synoretin |  |  |  | 160 | 5 | 2.41 | 4.01E-03 | 6.84E-02 |
| AF225987 | sodium channel, voltage-gated, type III, alpha | Hs.300717 | 2q24 | SCN3A | 94 | 5 | 2.42 | 1.87E-03 | 4.67E-02 |
| NM_032309 | chromosome 2 open reading frame 9 | Hs.375707 | 2q13 | C2orf9 | 223 | 16 | 2.43 | 5.25E-04 | 2.31E-02 |
| BC013393 | adducin 1 (alpha) | Hs.183706 | 4p16.3 | ADD1 | 5818 | 16 | 2.43 | 2.22E-03 | 5.12E-02 |
| AK027537 | fidgetin-like 1 | Hs.137516 | 7p12.2 | FIGNL1 | 113 | 9 | 2.43 | 3.41E-03 | 6.33E-02 |
| NM_032297 | hypothetical protein DKFZp761D112 | Hs.103849 | 8q21.3 | DKFZp761D112 | 101 | 8 | 2.43 | 4.02E-03 | 6.84E-02 |
| BC009725 | hypothetical protein FLJ38944 | Hs.135181 | 19q13.2 | FLJ38944 | 125 | 5 | 2.43 | 3.27E-03 | 6.22E-02 |
| AK027555 | Homo sapiens cDNA FLJ43665 fis, clone SYNOV4006327 | Hs.420262 | 3p21.2-p21.1 | ITIH1 | 143 | 12 | 2.44 | 2.38E-03 | 5.30E-02 |
| NM_004217 | aurora kinase B | Hs.442658 | 17p13.1 | AURKB | 6364 | 16 | 2.45 | 7.13E-05 | 7.43E-03 |
| NM_002178 | insulin-like growth factor binding protein 6 | Hs.274313 | 12q13 | IGFBP6 | 502 | 15 | 2.45 | 1.41E-05 | 2.66E-03 |
| NM_032356 | hypothetical protein MGC14151 | Hs.333414 | 17p13.2 | MGC14151 | 6364 | 16 | 2.45 | 8.36E-05 | 7.92E-03 |
| NM_015716 | misshapen/NIK-related kinase | Hs.112028 | 17p13.3 | MINK | 142 | 7 | 2.46 | 1.52E-03 | 4.26E-02 |
| NM_017775 | hypothetical protein FLJ20343 | Hs.171044 | 17p11.2 | FLJ20343 | 231 | 11 | 2.46 | 7.31E-03 | 9.19E-02 |
| NM_032365 | Hypothetical protein MGC5254 |  |  |  | 605 | 15 | 2.47 | 1.80E-03 | 4.64E-02 |
| NM_001212 | complement component 1, q subcomponent binding protein | Hs.78614 | 17p13.3 | C1QBP | 6583 | 15 | 2.48 | 1.17E-06 | 5.17E-04 |
| NM_019605 | hypothetical protein DJ667H12.2 | Hs.445835 | 1q32.1-q41 | DJ667H12.2 | 356 | 6 | 2.49 | 9.22E-04 | 3.26E-02 |
| NM_005022 | profilin 1 | Hs.408943 | 17p13.3 | PFN1 | 19578 | 16 | 2.53 | 4.15E-08 | 1.07E-04 |
| NM_002018 | flightless I homolog (Drosophila) | Hs.445182 | 17p11.2 | FLII | 3736 | 16 | 2.53 | 4.85E-06 | 1.42E-03 |
| AK056593 | hypothetical protein DKFZp313N0621 | Hs.55131 | 3p21.32 | DKFZp313N0621 | 65 | 5 | 2.54 | 3.07E-03 | 6.02E-02 |
| D50918 | septin 6 | Hs.90998 | Xq25 | SEPT6 | 370 | 11 | 2.54 | 3.66E-04 | 1.85E-02 |
| AB051439 | thioredoxin reductase 2 | Hs.443430 | 22q11.21 | TXNRD2 | 180 | 12 | 2.54 | 3.38E-05 | 4.73E-03 |
| AK027204 | mitochondrial ribosomal protein S22 | Hs.512649 | 3q23 | MRPS22 | 172 | 5 | 2.55 | 1.18E-03 | 3.73E-02 |
| NM_002013 | FK506 binding protein 3, 25kDa | Hs.379557 | 14q21.3 | FKBP3 | 179 | 12 | 2.57 | 1.82E-04 | 1.24E-02 |
| NM_014185 | RAN guanine nucleotide release factor | Hs.408233 | 17p13 | RANGNRF | 926 | 16 | 2.60 | 6.75E-05 | 7.33E-03 |
| AB011136 | KIAA0564 protein | Hs.405457 | 13q13.3 | KIAA0564 | 114 | 12 | 2.60 | 1.43E-03 | 4.15E-02 |
| NM_020904 | pleckstrin homology domain containing, family A (phosphoinositide binding specific) member 4 | Hs.9469 | 19q13.33 | PLEKHA4 | 175 | 14 | 2.61 | 1.84E-03 | 4.66E-02 |
| NM_014750 | discs, large homolog 7 (Drosophila) | Hs.77695 | 14q22.2 | DLG7 | 561 | 10 | 2.62 | 1.01E-04 | 8.85E-03 |
| NM_024658 | importin 4 | Hs.411865 | 14q11.2 | IPO4 | 169 | 12 | 2.63 | 3.53E-03 | 6.45E-02 |
| NM_000846 | glutathione S-transferase A2 | Hs.378199 | 6p12.1 | GSTA2 | 99 | 16 | 2.63 | 3.05E-03 | 6.01E-02 |
| AK000877 | Homo sapiens cDNA FLJ10015 fis, clone HEMBA1000392. | Hs.130661 | 8p23.1 | TDH | 328 | 14 | 2.64 | 1.58E-05 | 2.88E-03 |
| NM_007368 | RAS p21 protein activator 3 | Hs.119274 | 13q34 | RASA3 | 215 | 8 | 2.64 | 1.60E-03 | 4.34E-02 |
| NM_023004 | reticulon 4 receptor | Hs.30868 | 22q11 | RTN4R | 177 | 14 | 2.65 | 1.23E-04 | 1.01E-02 |
| X73617 | T cell receptor delta locus | Hs.2014 | 14q11.2 | TRD@ | 417 | 12 | 2.65 | 2.72E-04 | 1.56E-02 |
| AL117590 | hypothetical protein FLJ90406 | Hs.161394 | 5q21.1 | FLJ90406 | 222 | 14 | 2.67 | 1.44E-03 | 4.15E-02 |
| NM_003616 | survival of motor neuron protein interacting protein 1 | Hs.102456 | 14q13 | SIP1 | 858 | 15 | 2.67 | 3.22E-07 | 2.27E-04 |
| NM_020648 | twisted gastrulation homolog 1 (Drosophila) | Hs.247302 | 18p11.3 | TWSG1 | 310 | 15 | 2.67 | 3.59E-04 | 1.84E-02 |
| NM_001058 | tachykinin receptor 1 | Hs.1080 | 2p13.1-p12 | TACR1 | 89 | 5 | 2.71 | 4.07E-03 | 6.85E-02 |
| NM_000018 | acyl-Coenzyme A dehydrogenase, very long chain | Hs.437178 | 17p13-p11 | ACADVL | 2720 | 16 | 2.71 | 5.43E-05 | 6.54E-03 |
| NM_005367 | melanoma antigen, family A, 12 | Hs.169246 | Xq28 | MAGEA12 | 1063 | 15 | 2.73 | 8.98E-06 | 2.05E-03 |
| NM_001566 | inositol polyphosphate-4-phosphatase, type I, 107kDa | Hs.334575 | 2q11.2 | INPP4A | 210 | 11 | 2.74 | 9.38E-04 | 3.25E-02 |
| AL157425 | Homo sapiens mRNA; cDNA DKFZp761J1324 (from clone DKFZp761J1324) | Hs.133315 | 1q23.1 | IQGAP3 | 122 | 6 | 2.74 | 6.26E-03 | 8.41E-02 |
| AK024395 | Hypothetical protein FLJ11362 |  |  |  | 733 | 16 | 2.75 | 4.01E-07 | 2.59E-04 |
| AF290475 | Homo sapiens clone 124-1V1, mRNA sequence | Hs.72451 | 3q13.2-q13.31 | LOC152185 | 220 | 5 | 2.78 | 7.97E-04 | 3.00E-02 |
| NM_004176 | sterol regulatory element binding transcription factor 1 | Hs.426528 | 17p11.2 | SREBF1 | 1412 | 14 | 2.80 | 3.81E-04 | 1.88E-02 |
| NM_005576 | lysyl oxidase-like 1 | Hs.65436 | 15q24-q25 | LOXL1 | 347 | 16 | 2.81 | 6.46E-06 | 1.73E-03 |
| NM_001730 | Kruppel-like factor 5 (intestinal) | Hs.84728 | 13q21.33 | KLF5 | 263 | 13 | 2.83 | 7.28E-06 | 1.77E-03 |
| NM_021074 | NADH dehydrogenase (ubiquinone) flavoprotein 2, 24kDa | Hs.51299 | 18p11.31-p11.2 | NDUFV2 | 4695 | 15 | 2.84 | 1.04E-06 | 5.40E-04 |
| NM_002561 | purinergic receptor P2X, ligand-gated ion channel, 5 | Hs.408615 | 17p13.3 | P2RX5 | 144 | 8 | 2.86 | 1.09E-03 | 3.56E-02 |
| AK058159 | dehydrogenase/reductase (SDR family) member 1 | Hs.348350 | 14q11.2 | DHRS1 | 104 | 7 | 2.87 | 7.82E-03 | 9.48E-02 |
| NM_032751 | chromosome 14 open reading frame 128 | Hs.13810 | 14q13.1 | C14orf128 | 414 | 14 | 2.87 | 3.24E-04 | 1.76E-02 |
| NM_004480 | fucosyltransferase 8 (alpha (1,6) fucosyltransferase) | Hs.118722 | 14q24.3 | FUT8 | 449 | 14 | 2.88 | 6.56E-06 | 1.70E-03 |
| NM_032308 | hypothetical protein MGC4189 | Hs.355584 | 17p13.3 | MGC4189 | 557 | 16 | 2.88 | 1.09E-06 | 4.99E-04 |
| NM_017493 | Hin-1 | Hs.252722 | 4q28.1-q28.3 | HSHIN1 | 158 | 5 | 2.88 | 2.88E-03 | 5.82E-02 |
| NM_002428 | matrix metalloproteinase 15 (membrane-inserted) | Hs.80343 | 16q13-q21 | MMP15 | 312 | 13 | 2.88 | 6.86E-05 | 7.35E-03 |
| NM_001190 | branched chain aminotransferase 2, mitochondrial | Hs.512670 | 19q13 | BCAT2 | 608 | 16 | 2.89 | 9.59E-07 | 5.51E-04 |
| NM_000764 | cytochrome P450, family 2, subfamily A, polypeptide 7 | Hs.250615 | 19q13.2 | CYP2A7 | 97 | 15 | 2.89 | 5.12E-03 | 7.58E-02 |
| NM_032709 | hypothetical protein MGC13047 | Hs.118210 | 10q24.2 | MGC13047 | 90 | 7 | 2.91 | 5.46E-05 | 6.48E-03 |
| AK025390 | DKFZP586L0724 protein | Hs.26761 | 17q24.3 | DKFZP586L0724 | 111 | 10 | 2.91 | 7.80E-04 | 2.97E-02 |
| X77824 | Chromobox homolog 2 (Pc class homolog, Drosophila) |  |  |  | 104 | 8 | 2.91 | 8.50E-04 | 3.10E-02 |
| NM_003364 | uridine phosphorylase 1 | Hs.314828 | 7p12.3 | UPP1 | 2120 | 16 | 2.93 | 4.14E-05 | 5.36E-03 |
| NM_033380 | collagen, type IV, alpha 5 (Alport syndrome) | Hs.169825 | Xq22 | COL4A5 | 288 | 11 | 2.93 | 1.97E-03 | 4.82E-02 |
| BC008839 | chromosome 22 open reading frame 2 | Hs.334911 | 22q12 | C22orf2 | 67 | 6 | 2.95 | 3.34E-04 | 1.77E-02 |
| NM_001946 | dual specificity phosphatase 6 | Hs.298654 | 12q22-q23 | DUSP6 | 402 | 10 | 2.95 | 8.08E-03 | 9.63E-02 |
| NM_002961 | S100 calcium binding protein A4 (calcium protein, calvasculin, metastasin, murine placental homolog) | Hs.81256 | 1q21 | S100A4 | 117 | 8 | 2.96 | 1.66E-03 | 4.43E-02 |
| AK055545 | chromosome 14 open reading frame 31 | Hs.439190 | 14q22.1 | C14orf31 | 813 | 16 | 2.97 | 1.31E-05 | 2.58E-03 |
| NM_002945 | replication protein A1, 70kDa | Hs.84318 | 17p13.3 | RPA1 | 1988 | 16 | 2.97 | 3.92E-04 | 1.92E-02 |
| AF304052 | likely ortholog of mouse zinc finger protein 385 | Hs.278422 | 12q13.13 | ZFP385 | 155 | 10 | 2.99 | 1.21E-03 | 3.78E-02 |
| NM_002663 | phospholipase D2 | Hs.104519 | 17p13.1 | PLD2 | 269 | 14 | 3.00 | 5.08E-06 | 1.43E-03 |
| NM_006597 | heat shock 70kDa protein 8 | Hs.180414 | 11q24.1 | HSPA8 | 7820 | 16 | 3.01 | 1.53E-03 | 4.26E-02 |
| NM_001845 | collagen, type IV, alpha 1 | Hs.437173 | 13q34 | COL4A1 | 2916 | 14 | 3.01 | 1.44E-04 | 1.10E-02 |
| NM_025149 | hypothetical protein FLJ20920 | Hs.288959 | 17q21.33 | FLJ20920 | 131 | 12 | 3.02 | 7.31E-05 | 7.46E-03 |
| NM_005498 | adaptor-related protein complex 1, mu 2 subunit | Hs.18894 | 19p13.2 | AP1M2 | 1328 | 14 | 3.03 | 2.94E-06 | 9.72E-04 |
| NM_052966 | chromosome 1 open reading frame 24 | Hs.48778 | 1q25 | C1orf24 | 1245 | 16 | 3.04 | 6.16E-06 | 1.68E-03 |
| NM_018478 | chromosome 20 open reading frame 35 | Hs.256086 | 20q13.12 | C20orf35 | 213 | 15 | 3.06 | 2.05E-04 | 1.30E-02 |
| NM_002959 | sortilin 1 | Hs.394609 | 1p21.3-p13.1 | SORT1 | 83 | 12 | 3.07 | 2.57E-04 | 1.49E-02 |
| NM_004703 | rabaptin, RAB GTPase binding effector protein 1 | Hs.390163 | 17p13.3 | RABEP1 | 356 | 15 | 3.08 | 3.11E-04 | 1.72E-02 |
| NM_014214 | inositol(myo)-1(or 4)-monophosphatase 2 | Hs.5753 | 18p11.2 | IMPA2 | 632 | 16 | 3.09 | 5.25E-08 | 1.02E-04 |
| NM_007052 | NADPH oxidase 1 | Hs.132370 | Xq22 | NOX1 | 95 | 7 | 3.09 | 8.04E-03 | 9.61E-02 |
| NM_012137 | dimethylarginine dimethylaminohydrolase 1 | Hs.380870 | 1p22 | DDAH1 | 283 | 15 | 3.10 | 2.41E-03 | 5.33E-02 |
| NM_003958 | ring finger protein (C3HC4 type) 8 | Hs.24439 | 6p21.3 | RNF8 | 234 | 14 | 3.12 | 1.22E-03 | 3.78E-02 |
| AL157499 | rabaptin, RAB GTPase binding effector protein 1 | Hs.390163 | 17p13.3 | RABEP1 | 239 | 14 | 3.15 | 1.76E-05 | 3.01E-03 |
| NM_013342 | TCF3 (E2A) fusion partner (in childhood Leukemia) | Hs.233765 | 19q13 | TFPT | 124 | 6 | 3.17 | 4.19E-03 | 6.99E-02 |
| NM_018360 | chromosome X open reading frame 15 | Hs.201624 | Xp22.22 | CXorf15 | 205 | 10 | 3.17 | 1.42E-03 | 4.12E-02 |
| AK055564 | retinoic acid induced 3 | Hs.194691 | 12p13-p12.3 | RAI3 | 1044 | 16 | 3.20 | 2.85E-07 | 2.10E-04 |
| NM_000064 | complement component 3 | Hs.284394 | 19p13.3-p13.2 | C3 | 98 | 8 | 3.21 | 8.25E-04 | 3.06E-02 |
| NM_007361 | nidogen 2 (osteonidogen) | Hs.147697 | 14q21-q22 | NID2 | 142 | 11 | 3.26 | 3.70E-05 | 5.04E-03 |
| NM_001976 | enolase 3, (beta, muscle) | Hs.224171 | 17pter-p11 | ENO3 | 196 | 8 | 3.26 | 1.61E-03 | 4.36E-02 |
| NM_004554 | nuclear factor of activated T-cells, cytoplasmic, calcineurin-dependent 4 | Hs.77810 | 14q11.2 | NFATC4 | 146 | 5 | 3.31 | 6.59E-04 | 2.63E-02 |
| NM_001908 | cathepsin B | Hs.135226 | 8p22 | CTSB | 101 | 7 | 3.35 | 6.61E-03 | 8.71E-02 |
| NM_002131 | high mobility group AT-hook 1 | Hs.57301 | 6p21 | HMGA1 | 329 | 11 | 3.35 | 3.88E-05 | 5.15E-03 |
| NM_003355 | uncoupling protein 2 (mitochondrial, proton carrier) | Hs.80658 | 11q13 | UCP2 | 820 | 16 | 3.36 | 2.91E-04 | 1.62E-02 |
| NM_001416 | eukaryotic translation initiation factor 4A, isoform 1 | Hs.129673 | 17p13 | EIF4A1 | 3804 | 15 | 3.41 | 3.43E-05 | 4.71E-03 |
| AF174394 | Homo sapiens apoptotic-related protein PCAR mRNA, partial cds | Hs.395779 | 11p13 | CAT | 270 | 5 | 3.42 | 4.39E-04 | 2.06E-02 |
| NM_001878 | cellular retinoic acid binding protein 2 | Hs.183650 | 1q21.3 | CRABP2 | 140 | 8 | 3.47 | 7.50E-05 | 7.51E-03 |
| NM_024043 | hypothetical protein MGC3101 | Hs.301394 | 16q24.3 | MGC3101 | 246 | 8 | 3.52 | 3.26E-04 | 1.75E-02 |
| NM_002346 | lymphocyte antigen 6 complex, locus E | Hs.77667 | 8q24.3 | LY6E | 1632 | 15 | 3.52 | 3.43E-06 | 1.07E-03 |
| D87011 | Similar to Ovis aries Y chromosome repeat region OY11.1 |  |  |  | 132 | 11 | 3.54 | 3.23E-04 | 1.77E-02 |
| NM_014921 | latrophilin 1 | Hs.107054 | 19p13.2 | LPHN1 | 673 | 16 | 3.58 | 5.93E-08 | 1.02E-04 |
| BC017340 | serine/threonine kinase 35 | Hs.144794 | 20p13 | STK35 | 153 | 6 | 3.58 | 1.55E-03 | 4.28E-02 |
| NM_004169 | serine hydroxymethyltransferase 1 (soluble) | Hs.293636 | 17p11.2 | SHMT1 | 1770 | 16 | 3.59 | 3.94E-04 | 1.92E-02 |
| AL137326 | hypothetical protein FLJ37478 | Hs.318529 | 4p16.3 | FLJ37478 | 188 | 13 | 3.60 | 1.59E-04 | 1.14E-02 |
| NM_006271 | S100 calcium binding protein A1 | Hs.433503 | 1q21 | S100A1 | 324 | 11 | 3.60 | 1.93E-05 | 3.21E-03 |
| AK054976 | histidine triad nucleotide binding protein 1 | Hs.256697 | 5q31.2 | HINT1 | 78 | 6 | 3.62 | 6.02E-03 | 8.29E-02 |
| NM_000071 | cystathionine-beta-synthase | Hs.171003 | 21q22.3 | CBS | 362 | 16 | 3.63 | 1.72E-05 | 2.96E-03 |
| AK056446 | heat shock 90kDa protein 1, alpha | Hs.446579 | 14q32.33 | HSPCA | 2368 | 16 | 3.67 | 6.37E-03 | 8.49E-02 |
| AK022853 | homolog of rat orphan transporter v7-3 | Hs.44424 | 12q21.3 | NTT73 | 481 | 11 | 3.69 | 2.84E-06 | 9.58E-04 |
| NM_031422 | carbohydrate (N-acetylgalactosamine 4-0) sulfotransferase 9 | Hs.231943 | 18q11.2 | CHST9 | 107 | 7 | 3.70 | 1.92E-04 | 1.27E-02 |
| NM_001302 | cortistatin | Hs.412311 | 1p36 | CORT | 149 | 7 | 3.71 | 4.38E-03 | 7.11E-02 |
| NM_001235 | serine (or cysteine) proteinase inhibitor, clade H (heat shock protein 47), member 1, (collagen binding protein 1) | Hs.241579 | 11q13.5 | SERPINH1 | 1577 | 16 | 3.77 | 1.59E-05 | 2.83E-03 |
| NM_001885 | crystallin, alpha B | Hs.408767 | 11q22.3-q23.1 | CRYAB | 915 | 16 | 3.84 | 2.26E-06 | 8.17E-04 |
| NM_052886 | mal, T-cell differentiation protein 2 | Hs.76550 | Xp22.3 | MAL2 | 3388 | 14 | 3.89 | 1.26E-06 | 5.29E-04 |
| NM_012385 | p8 protein (candidate of metastasis 1) | Hs.418692 | 16p11.2 | P8 | 441 | 16 | 3.89 | 2.72E-05 | 4.14E-03 |
| NM_025099 | hypothetical protein FLJ22170 | Hs.156055 | 17p13.1 | FLJ22170 | 79 | 7 | 3.94 | 8.76E-03 | 9.92E-02 |
| U10689 | Melanoma antigen, family A, 5 |  |  |  | 215 | 10 | 3.95 | 1.06E-03 | 3.50E-02 |
| NM_002611 | pyruvate dehydrogenase kinase, isoenzyme 2 | Hs.92261 | 17q21.33 | PDK2 | 165 | 5 | 3.97 | 1.15E-04 | 9.62E-03 |
| NM_006892 | DNA (cytosine-5-)-methyltransferase 3 beta | Hs.251673 | 20q11.2 | DNMT3B | 434 | 16 | 3.99 | 7.17E-04 | 2.81E-02 |
| NM_006332 | interferon, gamma-inducible protein 30 | Hs.14623 | 19p13.1 | IFI30 | 417 | 13 | 4.17 | 8.08E-06 | 1.90E-03 |
| NM_000546 | tumor protein p53 (Li-Fraumeni syndrome) | Hs.426890 | 17p13.1 | TP53 | 1286 | 16 | 4.26 | 6.48E-06 | 1.71E-03 |
| NM_004827 | ATP-binding cassette, sub-family G (WHITE), member 2 | Hs.194720 | 4q22 | ABCG2 | 76 | 10 | 4.33 | 5.95E-03 | 8.24E-02 |
| NM_004657 | serum deprivation response (phosphatidylserine binding protein) | Hs.26530 | 2q32-q33 | SDPR | 1942 | 16 | 4.35 | 3.38E-06 | 1.07E-03 |
| AK024434 | hypothetical protein FLJ00024 | Hs.143878 | 9q34 | FLJ00024 | 102 | 8 | 4.39 | 5.03E-06 | 1.45E-03 |
| U79275 | hypothetical protein HSU79275 | Hs.27414 | 12q13.1 | HSU79275 | 542 | 15 | 4.63 | 7.08E-06 | 1.75E-03 |
| NM_003808 | tumor necrosis factor (ligand) superfamily, member 13 | Hs.54673 | 17p13.1 | TNFSF13 | 166 | 13 | 4.84 | 1.17E-05 | 2.42E-03 |
| AK056234 | Homo sapiens cDNA FLJ31672 fis, clone NT2RI2005048 |  |  |  | 124 | 6 | 4.90 | 1.98E-04 | 1.28E-02 |
| AF070632 | Homo sapiens clone 24405 mRNA sequence | Hs.23729 | 12q24.33 | PUS1 | 172 | 5 | 4.99 | 3.13E-03 | 6.12E-02 |
| NM_054012 | argininosuccinate synthetase | Hs.160786 | 9q34.1 | ASS | 646 | 14 | 5.32 | 9.42E-11 | 1.46E-06 |
| NM_002083 | glutathione peroxidase 2 (gastrointestinal) | Hs.2704 | 14q24.1 | GPX2 | 114 | 16 | 5.53 | 3.70E-07 | 2.50E-04 |
| NM_006843 | serine dehydratase | Hs.511764 | 12q24.21 | SDS | 416 | 12 | 5.59 | 6.35E-08 | 8.22E-05 |
| NM_000425 | L1 cell adhesion molecule (hydrocephalus, stenosis of aqueduct of Sylvius 1, MASA (mental retardation, aphasia, shuffling gait and adducted thumbs) syndrome, spastic paraplegia 1) | Hs.445201 | Xq28 | L1CAM | 253 | 11 | 5.61 | 2.80E-04 | 1.59E-02 |
| AK056857 | Sp8 transcription factor | Hs.195922 | 7p21.2 | SP8 | 113 | 10 | 5.84 | 8.26E-04 | 3.05E-02 |
| AB037730 | KIAA1309 protein |  |  |  | 511 | 15 | 5.96 | 9.10E-08 | 9.42E-05 |
| NM_032943 | synaptotagmin-like 2 | Hs.390463 | 11q14 | SYTL2 | 222 | 12 | 6.03 | 9.33E-07 | 5.57E-04 |
| AK056535 | Homo sapiens cDNA FLJ31973 fis, clone NT2RP7008144, highly similar to DUAL SPECIFICITY MITOGEN-ACTIVATED PROTEIN KINASE KINASE 4. | Hs.434117 | 6q23.1 | TAR3 | 92 | 5 | 6.16 | 3.23E-06 | 1.05E-03 |
| NM_003186 | transgelin | Hs.410977 | 11q23.2 | TAGLN | 2703 | 15 | 6.86 | 1.47E-05 | 2.76E-03 |
| BC012850 | calsenilin, presenilin binding protein, EF hand transcription factor | Hs.306828 | 2q21.1 | CSEN | 3055 | 16 | 7.61 | 1.40E-09 | 1.08E-05 |
| AF218006 | likely ortholog of neuronally expressed calcium binding protein | Hs.289242 | 2q37.1 | FLJ13612 | 426 | 14 | 9.10 | 5.95E-08 | 9.24E-05 |
| BE551792 | hypothetical protein LOC339400 | Hs.23783 | 1q21.3 | LOC339400 | 282 | 15 | 9.36 | 1.55E-07 | 1.27E-04 |
| NM_032385 | chromosome 5 open reading frame 4 | Hs.10235 | 5q31-q32 | C5orf4 | 170 | 5 | 9.46 | 5.44E-04 | 2.34E-02 |
| M95585 | hepatic leukemia factor | Hs.250692 | 17q22 | HLF | 68 | 5 | 10.00 | 1.53E-03 | 4.27E-02 |
| AF183421 | RAB31, member RAS oncogene family | Hs.223025 | 18p11.3 | RAB31 | 178 | 8 | 11.14 | 2.30E-07 | 1.79E-04 |
| NM_005532 | interferon, alpha-inducible protein 27 | Hs.278613 | 14q32 | IFI27 | 388 | 14 | 11.51 | 9.85E-06 | 2.15E-03 |
| AK022259 | hypothetical protein MGC13047 | Hs.118210 | 10q24.2 | MGC13047 | 110 | 7 | 12.82 | 1.20E-06 | 5.18E-04 |
| NM_015863 | Surfactant protein B |  |  |  | 237 | 13 | 14.72 | 2.35E-06 | 8.28E-04 |
| NM_004370 | collagen, type XII, alpha 1 | Hs.101302 | 6q12-q13 | COL12A1 | 82 | 7 | 25.78 | 9.23E-06 | 2.08E-03 |
| BC001787 | chromosome 14 open reading frame 143 | Hs.123232 | 14q32.11 | C14orf143 | 235 | 13 | 26.95 | 8.85E-08 | 9.82E-05 |
| NM_022783 | hypothetical protein FLJ12428 | Hs.87729 | 8q24.12 | FLJ12428 | 219 | 12 | 34.79 | 3.68E-08 | 1.14E-04 |
