# Supplementary material for: Folliculin Contributes to VHL Tumor Suppressing Activity in Renal Cancer through Regulation of Autophagy
Source: PLoS One. 2013 Jul 29;8(7):e70030. doi: 10.1371/journal.pone.0070030 (PMC3726479; doi:10.1371/journal.pone.0070030)
Supplement: Table S3 — Genes significantly reduced by VHL and enriched for specific chromosome or cytoband. (DOCX) [file pone.0070030.s004.docx]

**Table S3: Genes Significantly Induced by VHL (top) and Significantly Reduced by VHL (bottom) Enriched for Specific Chromosome or Cytoband.**

| **Up in VHL(-)** |  |  |  |  |  |  |  |  |  |  |  |
| --- | --- | --- | --- | --- | --- | --- | --- | --- | --- | --- | --- |
| **Category** | **Term** | **Count** | **%** | **PValue** | **Rank** | **FDR** | **List Total** | **Pop Hits** | **Pop Total** | **Fold Change** | **Genes** |
| CHROMOSOME | 7 | 63 | 9.75% | 9.90E-09 | 1 | 0.0000 | 638 | 591 | 12985 | 2.170 | NM_018641, NM_005746, NM_006193, AL137660, BC003517, NM_032158, NM_004126, NM_001505, NM_024653, NM_032831, AK026747, AL133568, AK025582, AL080209, AB040899, NM_017954, AK023427, AK055460, NM_017528, NM_001233, NM_001458, AB037823, NM_018295, AK021793, NM_005273, AF055019, NM_022740, NM_000596, NM_015379, BC011406, NM_004935, NM_000238, NM_021807, AB046845, NM_014399, AL137349, NM_007233, NM_031946, AK055660, AK027380, AK055922, NM_001259, NM_005720, NM_016265, AB047362, NM_002889, NM_013316, NM_001753, AL136883, NM_001305, AK055508, AK001887, NM_012447, NM_012395, AK000208, NM_016352, NM_003596, AF038190, NM_006754, NM_006528, AK056624, NM_001742, BC003517, NM_013389, NM_032842, |
| CHROMOSOME | 9 | 42 | 6.50% | 8.37E-04 | 2 | 0.0100 | 638 | 499 | 12985 | 1.713 | NM_006180, AK023330, NM_014279, NM_006195, NM_002957, AB037858, NM_004669, BC012155, AK024167, AK055437, NM_032318, NM_033334, AB058779, NM_003408, AK054606, NM_004059, NM_017617, AK023413, AK000939, M81635, NM_032928, NM_000689, AL137659, NM_024635, NM_002829, NM_004817, NM_004479, AF216077, NM_032012, AL117478, AK000637, AB002308, AF170307, NM_006808, AB014599, AK000144, BC011620, AB046844, BC015649, NM_032799, NM_001701, AB011145, |
| CYTOBAND | 9q34.3 | 11 | 1.70% | 1.35E-04 | 1 | 0.0170 | 638 | 50 | 12929 | 4.458 | NM_017617, NM_004479, NM_014279, NM_032928, BC015649, NM_002957, AL117478, NM_004669, AL137659, AB058779, AB002308, |
| CHROMOSOME | Y | 8 | 1.24% | 0.00258 | 3 | 0.0206 | 638 | 39 | 12985 | 4.175 | NM_002414, NM_004681, NM_002760, NM_001008, AF332224, AL049280, AL080135, AK026667, NM_004676, |
| CHROMOSOME | 4 | Not significant |  |  |  |  |  |  |  |  |  |
| CYTOBAND | 4p16.3 | 9 | 1.39% | 5.26E-04 | 2 | 0.0331 | 638 | 39 | 12929 | 4.677 | NM_007100, NM_002938, NM_001120, NM_000203, NM_003704, AL390128, NM_005663, NM_032326, NM_002337, |
