# Supplementary material for: Folliculin Contributes to VHL Tumor Suppressing Activity in Renal Cancer through Regulation of Autophagy
Source: PLoS One. 2013 Jul 29;8(7):e70030. doi: 10.1371/journal.pone.0070030 (PMC3726479; doi:10.1371/journal.pone.0070030)
Supplement: Table S4 — Sequence of primers for the indicated genes used in qRT-PCR. (DOCX) [file pone.0070030.s005.docx]

**Table S3: Sequence of Primers for the Indicated Genes Used in qRT-PCR**

| **Gene** | **Abbreviation** | **Access #** | **Primer forward** | **Primer reverse** |
| --- | --- | --- | --- | --- |
| elaC homolog 2 (E. coli) | ELAC2 | NM_018127 | TGGTTTGGAAGAGGAAGCAGTGGA | GTGGTCAAAGGCAACTCCCACTTT |
| Phosphatidylethanolamine-N-methyl transferase | PEMT | NM_148172 | CGGAGGCCTCGGCAATATTGATTT | GTGTTCCCATCGTGCAACCACATT |
| Serine hydroxymethyl transferase | SHMT1 | NM_004169 | TGGTGGTGTAGGGCAAAGCTGTTA | TGGACATGGGCATTTCCTCTCTGT |
| Sterol-regulatory element binding transcription factor1 variant 1 longer | SREBF1 | NM_001005291 | TAGAGCGAGCACTGAACTGTGTGA | TGCTGGAACTGATGGAGAAGCTGT |
| Mediator of RNA polymerase II transcription, subunit 9 | MED9 | NM_018019 | TCAAATGCATGGACAAGGACAGCC | GCTCATTCTTGGTCCTGACTTGCT |
| MYB binding protein (P160) 1a | MYBBP1A | NM_014520 | AGAAGCGCAAGAAACGCAAGTCAG | TGGGACCTTAGCCTTTGTCCTGTT |
| Replication protein A1, 70kDa | RPA1 | NM_002945 | TCGGGCAGAAGTTTGCAAATAGGC | TTCCTTTGCCTACCGATGGGTTCT |
| Retinoic acid induced | RAI1 | NM-030665 | TGATGTGTTCCAGCTGCCAAGAAG | TACGGCAGCCTCTTATGTTTGGGA |
| Epsin 2 variant 2, longest isoform | EPN2 | NM_014964 | TTCTCTGATGACCGAGATTGCCGA | GCCAGTTCTTGCCATGGTCATTCA |
| Flightless I homolog | FLII | NM_002018 | TGAGAACTTCTTCTGGGTGGGCAT | TCACTGCAAAGTAGCCCTTCTCGT |
| Tumor necrosis factor receptor superfamily member 13B variant 1 | TNFSF13 | NM_003808 | AGGTGATGTGGCAACCAGCTCTTA | TGACCCATGGTGAAAGTCACGTCT |
| COP9 constitutive photomorphogenic homolog subunit3 | COPS3 | NM_003653 | ACCAGAAGGACGGTATGGTCAGTT | TGTGATCTCCTGGTCCATGGCTTT |
| Lethal giant larvae homolog 1 | LLGL1 | NM_004140 | AAGAACCTGAGGAACCTGGCAGAA | AGGTTAGCAGGCGTGGTTCTATGT |
| Tripartite motif-containing 16 | TRIM16 | NM_006470 | TCTCGGGCATCCGCAAAGTTATCA | TATTTGCGCTGAACAACGGCAGAC |
| Serine hydroxymethyl transferase | SHMT1 | NM_004169 | TGGTGGTGTAGGGCAAAGCTGTTA | TGGACATGGGCATTTCCTCTCTGT |
| Folliculin | FLCN1 | NM_144997 | TCGCAATAACATGTCTGCTCCC | TTGCGGAGCCCTAACTCAATCACT |
| p53 | TP53 | NM_000546 | TTGCACCTACCTCACAGAGTGCAT | AGAAACTACCAACCCACCGACCAA |
